# Functional LTCC-β_2_AR Complex Needs Caveolin-3 and Is Disrupted in Heart Failure

**DOI:** 10.1161/CIRCRESAHA.123.322508

**Published:** 2023-06-14

**Authors:** Jose L. Sanchez-Alonso, Laura Fedele, Jaël S. Copier, Carla Lucarelli, Catherine Mansfield, Aleksandra Judina, Steven R. Houser, Thomas Brand, Julia Gorelik

**Affiliations:** 1 National Heart and Lung Institute, Imperial College London, United Kingdom (J.L.S.-A., L.F., J.S.C., C.L., C.M., A.J., T.B., J.G.).; 2 Department of Physiology, Cardiovascular Research Center, Lewis Katz Temple University School of Medicine, Philadelphia, PA (S.R.H.).

**Keywords:** calcium channels, L-type, cardiac electrophysiology, caveolin-3, heart failure, receptors, adrenergic, beta

## Abstract

Beta-2 adrenergic receptors (β_2_ARs) but not beta-2 adrenergic receptors (β_1_ARs) form a functional complex with L-type Ca^2+^ channels (LTCCs) on the cardiomyocyte membrane. However, how microdomain localization in the plasma membrane affects the function of these complexes is unknown. We aim to study the coupling between LTCC and β adrenergic receptors in different cardiomyocyte microdomains, the distinct involvement of PKA and CAMKII (Ca^2+^/calmodulin-dependent protein kinase II) and explore how this functional complex is disrupted in heart failure.

**Methods:**

Global signaling between LTCCs and β adrenergic receptors was assessed with whole-cell current recordings and western blot analysis. Super-resolution scanning patch-clamp was used to explore the local coupling between single LTCCs and β_1_AR or β_2_AR in different membrane microdomains in control and failing cardiomyocytes.

**Results:**

LTCC open probability (Po) showed an increase from 0.054±0.003 to 0.092±0.008 when β_2_AR was locally stimulated in the proximity of the channel (<350 nm) in the transverse tubule microdomain. In failing cardiomyocytes, from both rodents and humans, this transverse tubule coupling between LTCC and β_2_AR was lost. Interestingly, local stimulation of β_1_AR did not elicit any change in the Po of LTCCs, indicating a lack of proximal functional interaction between the two, but we confirmed a general activation of LTCC via β_1_AR. By using blockers of PKA and CaMKII and a Caveolin-3-knockout mouse model, we conclude that the β_2_AR-LTCC regulation requires the presence of caveolin-3 and the activation of the CaMKII pathway. By contrast, at a cellular “global” level PKA plays a major role downstream β_1_AR and results in an increase in LTCC current.

**Conclusions:**

Regulation of the LTCC activity by proximity coupling mechanisms occurs only via β_2_AR, but not β_1_AR. This may explain how β_2_ARs tune the response of LTCCs to adrenergic stimulation in healthy conditions. This coupling is lost in heart failure; restoring it could improve the adrenergic response of failing cardiomyocytes.

**Novelty and Significance**

**What Is Known?**

- Beta-1 adrenergic and beta-2 adrenergic receptors (β_1_AR and β_2_AR) modulate the activity of L-type calcium channels.
- Heart failure alters cardiac function in several ways, including dysregulation of βAR signaling and ion channels.
- L-type calcium channels are considered an important component of alterations seen in heart failure cells.

**What New Information Does This Article Contribute?**

- When βAR are stimulated in the proximity of a LTCC, only β_2_AR can modulate the channel, not β_1_AR.
- This β_2_AR-LTCC interaction requires the presence of caveolin-3 and the activation of the CaMKII (Ca^2+^/calmodulin-dependent protein kinase-II) pathway.
- This local coupling is not present in heart failure.

LTCCs are distributed in the plasma membrane of cardiomyocytes in different microdomains. In some locations LTCC function can be modulated by other signaling partners, including βAR. In HF, this modulation can be altered, leading to the dysregulation of LTCC.

Using electrophysiological techniques, including SICM smart patch, and molecular biology we describe how the local stimulation of βAR leads to a modulation of LTCC by β_2_AR but not β_1_AR, indicating a functional β_2_AR-LTCC complex that is present in rat, mouse, and human cardiomyocytes. By using pharmacological treatments to block protein kinase A and CaMKII, and a Cav3KO mouse model, we conclude that this local functional β_2_AR-LTCC complex requires the presence of caveolin-3 and the activation of CaMKII pathway. Interestingly, on cardiomyocytes from an animal model of heart failure, this β_2_AR-LTCC coupling is not present, neither it was found on human cardiomyocytes from dilated cardiomyopathies patients.

This local β_2_AR-LTCC complex may have a fundamental role in maintaining the balance of the βAR modulation of LTCC. In failing myocytes, the disruption of the complex may be part of maladaptive changes leading to heart failure. Aiming to restore the proper organization of the β_2_AR-LTCC complex could be beneficial for restoring the function of failing cardiomyocytes.

**In This Issue, see p 105**

**Meet the First Author, see p 106**

In cardiomyocytes, L-type calcium channels (LTCCs) are crucially involved in excitation-contraction coupling, action potential duration, and regulation of gene expression. Among the different LTCC isoforms, Cav1.2 channels have been extensively characterized in cardiomyocytes. Dysregulation of their activity and location has been shown to be crucially important in the failing heart and has been associated with arrhythmogenesis and sudden cardiac death.^1,2^

LTCCs are distributed on the surface of cardiomyocytes in different microdomains.^3^ One of the key contributors in the enhancement of the LTCC current (I_Ca,L_) in cardiomyocytes occurs downstream the stimulation of β_1_ and β_2_ adrenergic receptors (βARs).^3^ Notably, the downstream increase of I_Ca,L_ upon β_2_AR stimulation seems to arise only in the proximity of the channel, suggesting a coupling between LTCC and β_2_AR.^4^ Consistent with these results, LTCCs can form a complex with β_2_AR and other proteins, but not with beta-1 adrenergic receptor (β_1_AR).^5,6^ The functional complex between LTCC and beta-2 adrenergic receptor (β_2_AR) is exclusive to specific locations, β_2_AR signaling has been shown to be confined in the transverse tubules (TT) in the plasma membrane.^7^ The existence of distinct functional subpopulation of β_2_ARs regulating LTCC activity, playing different roles depending on the kinases involved, has been shown in other cell types. For example, in hippocampal neurons,^8^ PKA-phosphorylated β_2_AR are in the dendrites, while GRK (G protein receptor kinase)-phosphorylated β_2_AR are mainly localized in the cell soma, with PKA playing a key role in increasing I_Ca,L_. Notably, the stimulation of β_2_AR can induce the compartmentalization of β_1_AR signaling,^9^ providing another level of complexity in the pathways involved in the regulation of LTCCs.

It is largely unknown how the β_2_AR-LTCC functional coupling is impaired in failing cardiomyocytes. Heart failure (HF) is characterized by impaired βAR signaling, which manifests in different ways, for example: an increase in β_2_AR signaling due to the decrease in β_1_AR expression^10^; an attenuated augmentation of the LTCC current by beta-adrenergic stimulation^11^; and the restrictive β_2_AR cAMP signaling in the TT become global and less dependent on phosphodiesterase inhibition.^7^

The main regulatory pathway that increase the LTCC current^12^ upon βAR stimulation, has been proposed to be PKA-dependent phosphorylation downstream the increase of cAMP.^13^ Accordingly, the C terminus of the LTCC alpha subunit presents several phosphorylation sites for PKA.^14^ However, cAMP-independent mechanisms of calcium current enhancement have also been described.^15,16^ More recently it was shown that Cav1.2 can be stimulated by βARs without the need of PKA phosphorylation of the alpha subunit,^17^ and that the noncanonical G-protein Rad was involved in this mechanism.^18^ Cav1.2 can also be phosphorylated by other proteins, including, CaMKII (Ca^2+^/calmodulin-dependent protein kinase II).^19^ CaMKII, in fact, is also activated downstream βARs^20^ and has been suggested to be a promising therapeutic target in HF treatment.^21^

In this work, we aim to better understand the mechanism underlying the functional coupling between LTCC and βARs in different cardiomyocyte microdomains and explore if they are disrupted in HF. We studied the effect of βARs stimulation on LTTCs as well as the selective stimulation of β_1_ARs or β_2_ARs both at the single channel and whole-cell I_Ca,L_ level. We also assessed the involvement of CaMKII (Ca^2+^/calmodulin-dependent protein kinase II) and PKA, using a combination of Western blotting and electrophysiology, that is, employing selective blockers for PKA and CaMKII in the pipette solution to assess their contribution upon βARs stimulation both at whole-cell and at microdomain levels. Moreover, we examined how the β_2_ARs-LTCC functional complex is perturbed in a rat myocardial infarct model and in human dilated cardiomyopathy. Finally, given the importance of caveolae in the β_2_AR and LTCC complex,^5,22^ we employed different methods to disrupt the structure of caveolae (chemical disruption, specific blockers, and Caveolin-3-knockout [Cav3KO] mice) to study potential impairments in LTCC modulation upon stimulation of β_2_AR.

Our findings elucidate the distinct role of CaMKII and PKA in the functional β_2_ARs-LTCC microdomains and their involvement at a global/cellular level in control cardiomyocytes. We also contribute to a deeper understanding of the impairment of βARs in diseased states opening up potential targets that could be further explored in future research on HF.

## Methods

### Data Availability

The authors declare that all supporting data are available within the article (and its online Supplemental Material). For full details of methods, please see the Supplemental Material.

### Study Approval

Animal experiments were carried out under the approval of the Animal Welfare and Ethics Review Board (AWERB) of Imperial College London, in accordance with the United Kingdom Home Office Guide on the Operation of the Animals (Scientific Procedures) Act 1986 and EU Directive 2010/83.

Experiments on isolated human cardiomyocytes were approved by Imperial College Institutional Review Board, with informed consent taken from each patient. End-stage HF samples were used with the approval from Brompton Harefield & NHLI Ethics Committee under Biobank REC approval reference 09/H0504/104+5. Donor hearts samples were used with the approval of NHS BT with REC approval reference: 16/LO/1568.

## Results

### Whole-Cell Stimulation of LTCC by βAR Activation

**Figure 1.**
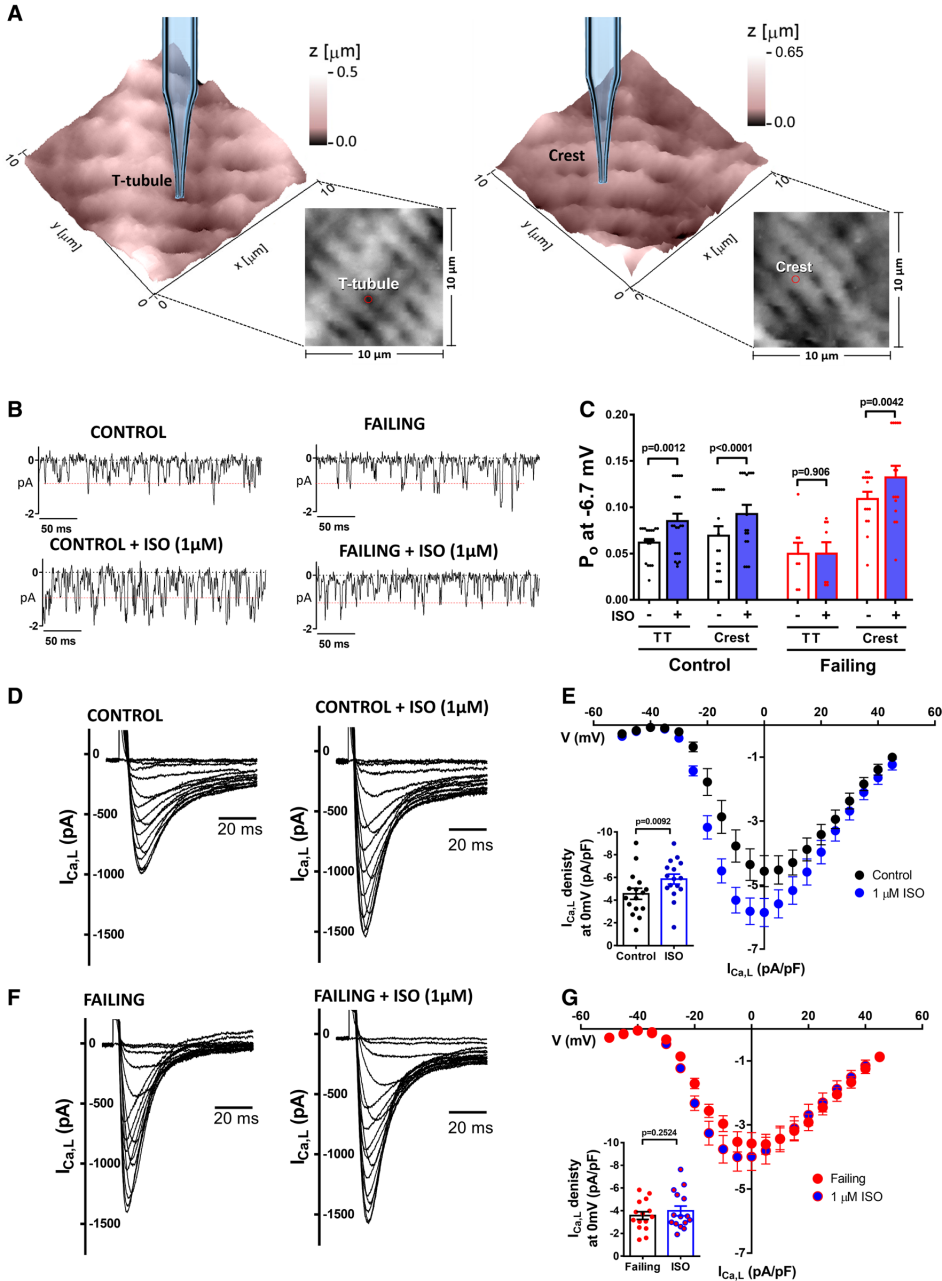
**L-type Ca^2+^ channels (LTCCs) response to isoproterenol on control and failing cardiomyocytes. A**, 10×10 µm representative scanning ion conductance microscopy topographical images of control cardiomyocytes showing the position of the pipette on the TT or Crest microdomain. **B**, Representative single LTCC traces at −6.7 mV. **C**, Summary graph of the Po from control and failing cardiomyocytes in TT and Crest before and after 1 µM ISO application (n=channels/cells/animals TT control 21/8/6, Crest control 17/7/5, TT failing 8/5/4, Crest failing 15/7/7, *P* values by Wilcoxon matched-pairs signed rank test). **D**, Representative I_Ca,L_ traces of a control cell before and after 1 µM ISO application. **E**, I/V graph of control cells, inset showing the differences at 0 mV (n=16/5, *P<0.05; *P* value by Wilcoxon matched-pairs signed rank test). **F**, Representative I_Ca,L_ traces of a failing cell before and after 0.1 µM ISO application. **G**, I/V graph of failing cells, inset showing the differences at 0 mV (n=15/4, *P* value by Wilcoxon matched-pairs signed rank test).

The relationship between LTCC and βARs has been studied for decades and it is well established that the inotropic effect upon βARs stimulation also includes an increase in the activity of LTCC.^23^ Using smart patch-clamp, we investigated if the LTCCs from different microdomains could respond differently to βARs activation by patching in the TT or Crest domain after producing a topographical map (Figure 1A). Following each successful recording of a single LTCC, 1 μM of isoproterenol (ISO) was applied in the bath. Representative traces are shown in Figure 1B. ISO resulted in a significant increase in the LTCC open probability (Po) in control cells (Figure 1C; *P*=0.0012), with a comparable change in both TT and Crest (TT:40% increase versus Crest:50% increase, Figure S1A). The amplitude of LTCC only increased in the TT, but the effect was small (3.26±0.86%; Figure S1B and S1C). In failing rat cardiomyocytes isolated from animals 16-week post myocardial infarction (MI), ISO did not elicit a Po increment on the TTs (Figure 1C). Interestingly, LTTCs in the crest from failing cells showed a reduced response to ISO compared to control cells. Notably, according to previously published reports failing Crest LTCCs presented a higher Po at baseline.^2^ Whole-cell I_Ca,L_ was also recorded before and after ISO (1 μM) in control and failing cells (Figure S1D). As expected, ISO produced an increase in the peak density of I_Ca,L_ in control cells (*P*=0.0092, Figure 1D and 1E), but not on failing cells (Figure 1F and 1G). Time constant of inactivation at baseline were measured in both control and failing cells, showing that times of decay of I_Ca,L_ from failing cells are slower (Figure S1E and S1F), in agreement with previous publication.^2^ Capacitance analysis of these cells confirmed the hypertrophic phenotype of failing cells (control: 237.7±9.24 pF, n=42/11, versus failing: 361.4±24.48 pF, n=21/4, *P*<0.0001, by unpaired *t* test).

**Figure 2.**
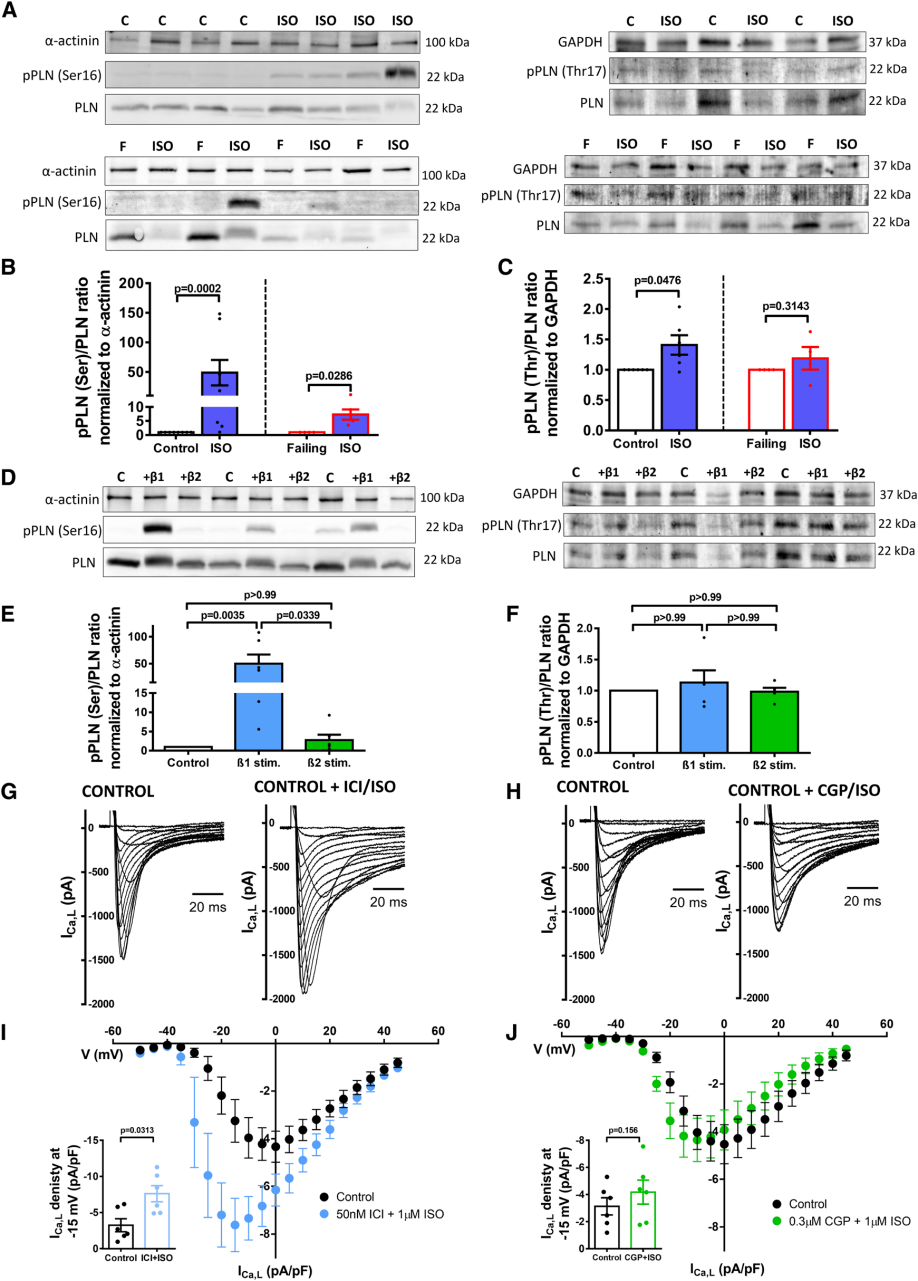
**Protein phosphorylation analysis by WB and ICa,L response to beta-2 adrenergic receptor (β_1_AR) or beta-2 adrenergic receptor (β_2_AR) stimulation. A**, Representative WBs of control and failing samples. **B**, Summary graph of densitometry analysis of pPLN for the PKA site (Ser16) vs total PLN, normalized to α-actinin from control (n=8) and failing (n=4) rat cardiomyocytes isolations (*P* values by Mann-Whitney *U* test). **C**, Summary graph of densitometry analysis of pPLN for CaMKII (Ca^2+^/calmodulin-dependent protein kinase II) site (Thr17) vs total PLN, normalized to GAPDH from control (n=6) and failing (n=4) rat cardiomyocytes isolations (*P* values by Mann-Whitney *U* test). **D**, Representative WBs of control cells under no stimulation, under β_1_AR stimulation, or under β_2_AR stimulation. **E**, Summary graph of densitometry analysis of pPLN (Ser16) vs total PLN, normalized to α-actinin from control cardiomyocytes after β_1_AR or β_2_AR stimulation (n=6, *P* values by Kruskal-Wallis followed by Dunn multiple comparisons test). **F**, Summary graph of densitometry analysis of pPLN (Thr17) vs total PLN, normalized to GAPDH from control cardiomyocytes after β_1_AR or β_2_AR stimulation (n=5, *P* values by Kruskal-Wallis followed by Dunn’s multiple comparisons test). **G**, Representative I_Ca,L_ traces of a control cell before and after 0.05 µM ICI and 1 µM ISO application. **H**, Representative I_Ca,L_ traces of a control cell before and after 0.3 µM CGP and 1 µM ISO application. **I**, I/V graph of control cells before and after β_1_AR stimulation, inset showing the differences at −15 mV (n=6/3, *P* value by Wilcoxon matched-pairs signed rank test). **J**, I/V graph of control cells before and after β_2_AR stimulation, inset showing the differences at −15 mV (n=6/3, *P* value by Wilcoxon matched-pairs signed rank test). PLN indicates phospholamban; pPLN, phosphorylated phospholamban; and WB, western blot.

The effect of βARs on LTCC is mediated through the activation of protein kinases, mainly PKA and CaMKII. To investigate the contribution of these two kinases, control and failing cardiomyocytes were incubated for 15 minutes with or without ISO (0.1 μM) and the activity of these kinases was analyzed by Western blotting (WB). Specific antibodies to detect PKA-dependent Ser-16 phosphorylation of phospholamban (pPLNSer16), CaMKII-dependent Thr-17 phosphorylation of phospholamban (pPLNThr17), and phosphorylated CaMKII (pCaMKII) were employed (Figure 2A; Figure S2). In control cells, ISO stimulation elicited a strong 50-fold increase of pPLNSer16 (PKA) expression (*P*=0.0002) and a 1.4-fold increase of pPLNThr17 (CaMKII) expression (*P*<0.0286, Figure 2B). Consistent with our electrophysiological results, we observed a smaller, yet significant, 5-fold increase of pPLNSer16 expression (*P*=0.0476) upon ISO incubation, and no differences of pPLNThr17 expression (Figure 2C) on failing cells. The limited changes of CaMKII activity observed by pPLNThr17 were also confirmed by using an antibody to directly measure the expression of pCaMKII (Figure S2).

These results suggest that the phosphorylation of the channels through global βAR stimulation is independent of their localization (TT or Crest) and mediated by PKA, with a minor contribution of CaMKII. Furthermore, failing cardiomyocytes present a reduced response to ISO evident by our data on single channel LTCC, whole-cell I_Ca,L_, and WB.

### The Increase of PKA Upon βAR Stimulation Occurs via β_1_AR

Since no differences between TT and Crest were found after ISO stimulation, we aimed to assess the contribution of β_1_AR and β_2_AR, given the close physical communication between LTCC and β_2_AR^5,6^ and their characteristic distribution in the cardiomyocyte membrane.^7^ We therefore investigated which kinase was activated upon β_1_AR or β_2_AR stimulation, as this would underlie the pathway involved in the modulation of LTCCs.

Cells were incubated for 15 minutes with 1 µM ISO+0.05 µM ICI (β_1_AR_stimulation_) or 1 µM ISO+0.3 µM CGP (β_2_AR_stimulation_), or with the same solution without any additional drug (control). WB was performed to detect PKA and CaMKII activity as before (Figure 2D). β_1_AR stimulation resulted in a strong increase of pPLNSer16 (50-fold increase, *P*=0.0035, Figure 2E) without a statistically significant effect on pPLNThr17 (1.2-fold, *P*=0.0339, Figure 2E). In contrast, β_2_AR stimulation did not lead to any statistically significant effect (Figure 2F).

Whole-cell I_Ca,L_ was recorded at baseline and following β_1_AR or β_2_AR stimulation. In agreement with the WB data, β_1_AR stimulation produced a significant increase of the I_Ca,L_ peak density (*P*=0.0313, Figure 2G and 2I), while β_2_AR stimulation did not produce a significant change of the peak density (Figure 2H and 2J). Confirming the lack of effect of β_2_AR on whole I_Ca,L_ showed by previous work from Harding’s group.^24^

**Figure 3.**
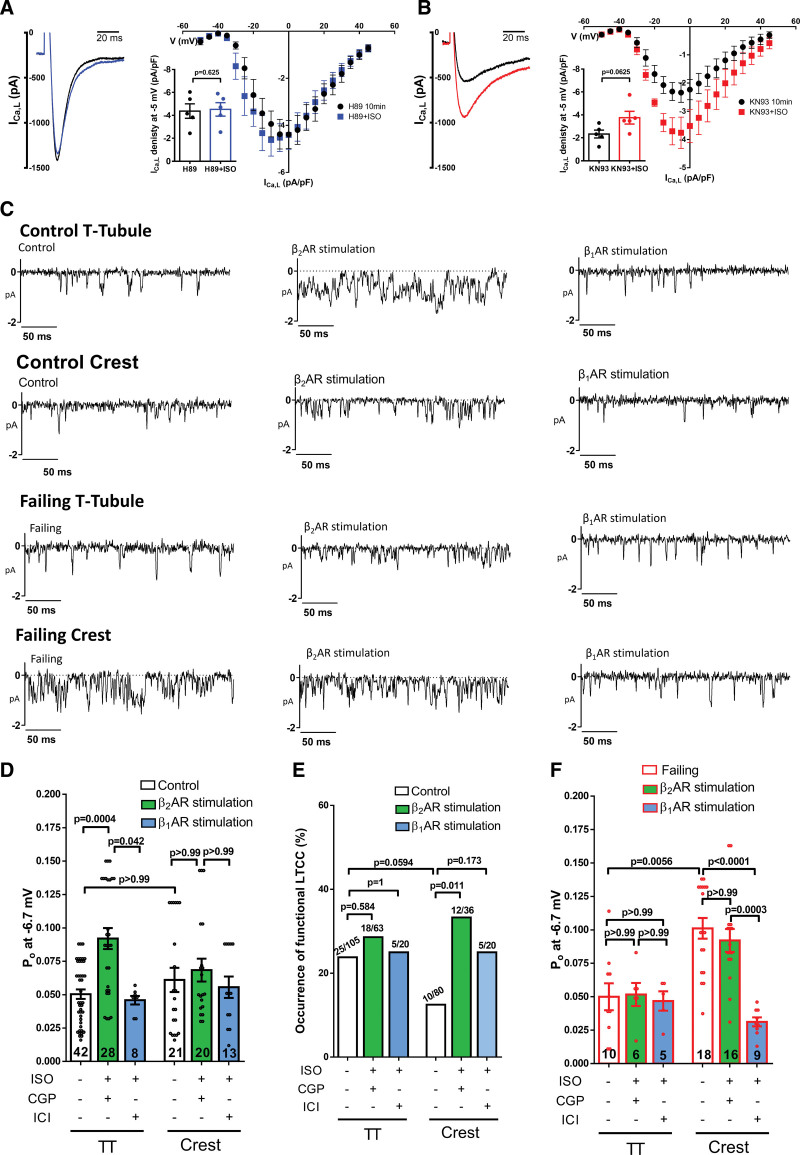
**L-type Ca^2+^ channels (LTCCs) changes between control and failing cardiomyocytes under local βAR stimulation. A**, Left, representative traces of I_Ca,L_ currents before and after ISO application (1 µM) in the presence of PKA inhibitor (H89 10 µM). **Right**, summary I/V graph, inset showing the differences at −5 mV (n=5/3, *P* value by Wilcoxon matched-pairs signed rank test). **B**, **Left**, representative traces of I_Ca,L_ currents before and after ISO application (1 µM) in the presence of CaMKII (Ca^2+^/calmodulin-dependent protein kinase II) inhibitor (KN-93 10 µM). Right, Summary I/V graph, inset showing the differences at 0 mV (n=5/2, *P* value by Wilcoxon matched-pairs signed rank test). **C**, Representative traces of single LTCC recordings at −6.7 mV. **D**, Summary graph of the Po from control cardiomyocytes in TT and crest under local beta-2 adrenergic receptors (β_1_AR) or beta-2 adrenergic receptor (β_2_AR) stimulation (n=channels/cells/animals; 42/21/12, 28/15/9, 8/5/4, 21/10/8, 20/13/7, 13/5/2, *P<0.05,**P<0.01, by Kruskal-Wallis followed by Dunn multiple comparisons test). **E**, Summary graph of the chance of obtaining a LTCC current (% of occurrence). It represents the number of recordings with LTCC activity (left number in the bar) vs the total number of recordings done in a specific microdomain and group (right number in the bar; *P* values by Fisher exact test). **F**, Summary graph of the Po from failing cardiomyocytes in TT and Crest under local β_1_AR or β_2_AR stimulation (n=channels/cells/animals; 10/7/5, 6/4/3, 5/4/3, 18/9/7, 16/9/4, 9/8/3, **P<0.01, ***P<0.001, by Kruskal-Wallis followed by Dunn multiple comparisons test). PKA indicates protein kinase A.

We further explored the involvement of PKA and CaMKII downstream the global stimulation of βARs on I_Ca,L_ with the employment of PKA and CAMKII blockers, respectively H-89 (10 μM) and KN-93 (10 μM). Consistent with a major role of PKA upon βARs stimulation, whole-cell currents with H-89 in the pipette solution abolished any effect of ISO on I_Ca,L_ (Figure 3A). By contrast, ISO still increased I_Ca,L_ in cells recorded with KN-93 in the pipette solution (Figure 3B), suggesting a minor contribution of CaMKII in this pathway.

### Local Stimulation of LTCC Through β_1_AR or β_2_AR

It was elegantly shown by Lakatta’s group that LTCCs can be stimulated remotely by β_1_AR, far from the channel subcellular localization.^4^ By contrast, β_2_AR stimulation requires proximity of the receptor to the channel. In classic single-channel cell-attached recording, the standard pipette resistance is around 3 to 5 MΩ, which could be estimated to be higher than 1 to 2 µm diameter. In our experimental conditions, to observe differences between TT and Crest, the diameter of the pipette is kept in the range of 300 to 450 nm (25–40 MΩ). Although the chance to find active channels in the seal is reduced, when the channels are recorded, we can restrict the area of stimulation to just the area surrounding the channel by adding an agonist or antagonist to the pipette solution.

After obtaining a topographical map, the pipette was positioned in the TT or Crest microdomain. Channels were recorded under β_1_AR (1 µM ISO+0.05 µM ICI), or β_2_AR (1 µM ISO+0.3 µM CGP) local stimulation and compared to channel recordings without stimulation (Figure 3C). We observed an increase of LTCC Po by local β_2_AR stimulation in the TT (control: 0.0504±0.004 versus β_2_AR_stimulation_: 0.092±0.008, *P*=0.0004; Figure 3D). However, this did not happen in the crest, where the LTCC Po remained unchanged (control: 0.061±0.009 versus β_2_AR_stimulation_: 0.068±0.009, Figure 3D). Interestingly, we did not observe any changes under local β_1_AR stimulation. The conductance of the channels was also analyzed confirming the same effect: an increase of approximately 30% under local β_2_AR stimulation but no effect under local β_1_AR stimulation (Figure S3). In most of the groups a slight increase in LTCC occurrence under local β_2_AR stimulation was noted. We also compared the occurrence of functional LTCC channels: statistically significant changes were observed only for local β_2_AR stimulation in the Crest by Fisher’s exact test (*P*=0.011, Figure 3E).

To further investigate the reduced response of failing cell to ISO, we recorded LTCC under local β_1_AR and β_2_AR stimulation from different microdomains. No changes in Po or conductance were observed in failing cardiomyocytes after β_2_AR local stimulation, neither in the TT (MI-TT:0.05±0.01 versus MI-β_2_AR_stimulation_-TT:0.052±0.009) nor in the Crest (MI-Crest:0.101±0.008 versus MI-β_2_AR-_stimulation_-Crest:0.092±0.009; Figure 3F). Interestingly, in contrast to the control crest channels where β_1_AR stimulation was ineffective, the failing cells under β_1_AR stimulation presented a 3-fold reduction in Po (MI- β_1_AR_stimulation_-Crest:0.031±0.003 versus MI-Crest:0.101±0.008, *P*<0.0001, Figure 3F).

These results suggest that the coupling between LTCC and β_2_AR in the TT microdomain is impaired in failing cells, possibly lost or the complex is displaced to the crest domain.

### Mechanism Underlying the Coupling Between LTCC and β_2_AR

**Figure 4.**
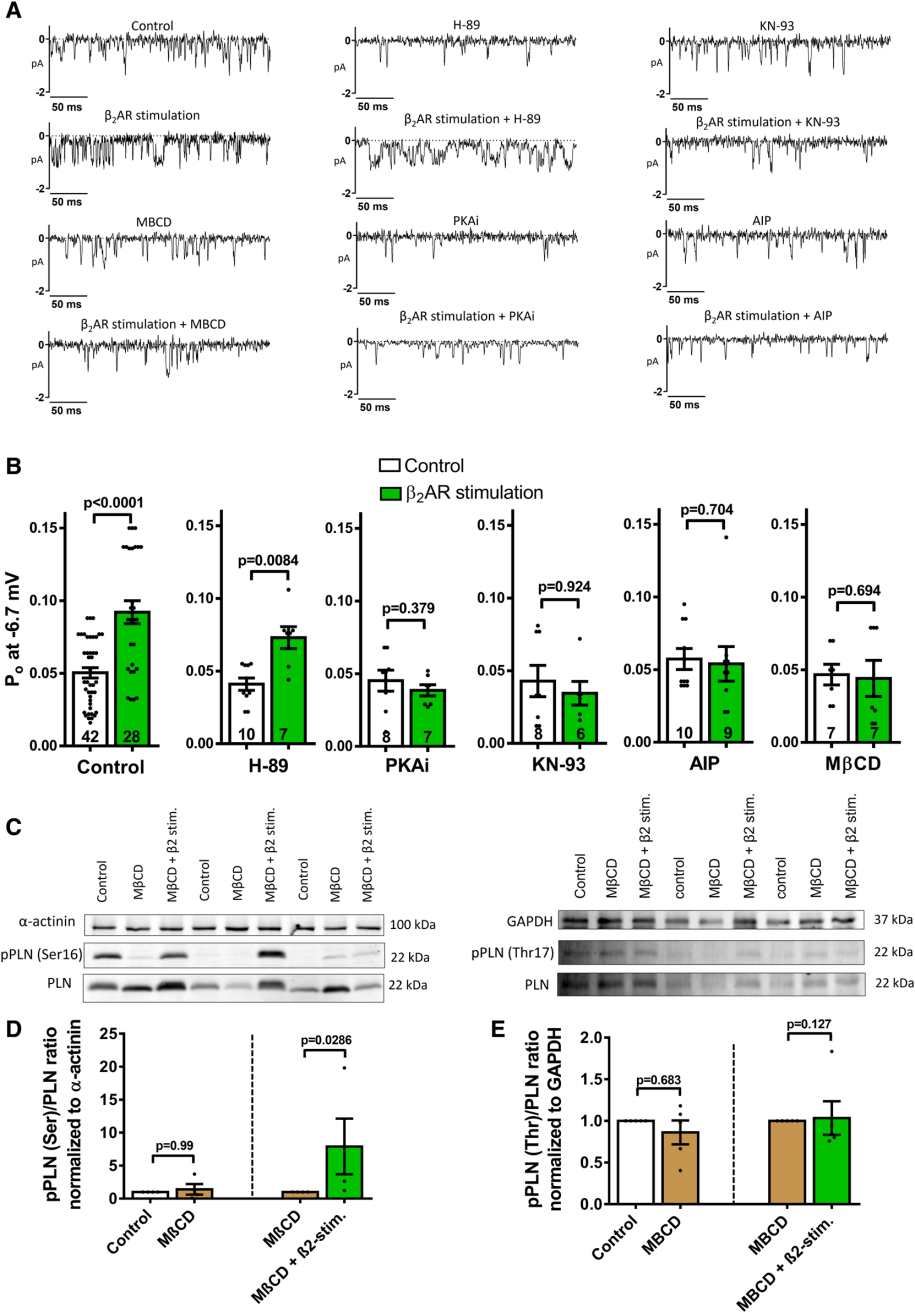
**Mechanism behind the local interaction between L-type Ca^2+^ channel (LTCC) and β_2_AR on the TT. A**, Representative traces of single LTCC recordings at −6.7 mV. **B**, Summary graph of the Po from control cardiomyocytes after different preincubation treatments, for PKA inhibition (with H-89 10 µM or PKAi 3 µM), for CaMKII (Ca^2+^/calmodulin-dependent protein kinase II) inhibition (with KN-93 10 µM or AIP 5 µM), and for cholesterol removal (MβCD; n=channels/cells/animals; 42/21/12, 28/15/9, 10/7/4, 7/5/3, 8/6/4, 7/5/4, 8/5/5, 6/6/4, 10/5/2, 9/6/3, 7/4/2, 7/4/2; *P* values by Mann-Whitney *U* test). **C**, Representative WBs of control cardiomyocytes treated with MβCD with or without β_2_AR stimulation. **D**, Summary graph of densitometry analysis of pPLN normalized to α-actinin from control cardiomyocytes after MβCD with or without β_2_AR stimulation (n=4, *P* values by Mann-Whitney *U* test). **E**, Summary graph of densitometry analysis of CaMKII normalized to GAPDH from control cardiomyocytes after MβCD with or without β_2_AR stimulation (n=5, *P* values by Mann-Whitney *U* test). MβCD indicates methyl-β-cyclodextrin; and PKAi, inhibitory protein kinase peptide.

To investigate the mechanism of β_2_AR-LTCC coupling, several pharmacological treatments were performed on TT LTCCs in control cells (Figure 4A).

We first assessed the contribution of PKA on β_2_AR local stimulation. When PKA was inhibited by H-89 (10 μM), the local stimulatory effect of β_2_AR on LTCC was still present when compared with LTCCs from cardiomyocytes treated with H-89 alone (H-89 Po: 0.046±0.005 versus β_2_AR stimulation H-89 Po: 0.066±0.004, *P*=0.0084, Figure 4B). However, when PKA was inhibited by the inhibitory peptide (PKAi, 3 µM) β_2_AR stimulation was unable to elicit a response (PKAi Po: 0.045±0.007 versus β_2_-PKAi Po: 0.039±0.004, Figure 4B).

Secondly, we tested the involvement of CaMKII. When CaMKII was inhibited by KN-93 (10 µM), the β_2_AR stimulatory effect was blocked (KN-93 Po: 0.043±0.011 versus β_2_AR-_stimulation_-KN-93 Po: 0.035±0.008, Figure 4B) and a similar effect was seen when using the inhibitory peptide AIP (5 µM; AIP Po: 0.057±0.007 versus β_2_AR_stimulation_-AIP Po: 0.054±0.012, Figure 4B).

The lack of effect of β_2_AR stimulation of LTCC localized to the TT of failing cells suggested that this coupling was lost. However, as we previously published, crest LTCC from failing cells could be constitutively phosphorylated,^2^ suggesting an overactivation of this pathway. The reduction of the Po exclusively on the Crest channels upon β_1_AR stimulation in failing cells (Figure 3D) might be due to an inhibitory effect of the β_2_AR blocker ICI in these cells. Consistent with this hypothesis, ICI used alone in the pipette produced the same inhibitory effect (MI-ICI Po: 0.031±0.003, *P*=0.0007, Figure S4). This result indicates that the reduction of Po in the failing Crest LTCCs is mediated via the ICI inhibition of β_2_AR, not through the stimulation of β_1_AR by ISO in the pipette. CaMKII has also been linked to the increased Po activity of crest LTCC from failing cells. As previously shown,^2^ KN-93 can reduce the Po of failing crest LTCC from 0.101±0.008 to 0.029±0.003 (*P*=0.0049). The presence of KN-93 also prevented any effect of β_2_AR stimulation on these channels (0.023±0.004, Figure S4).

In summary, these results suggest that coupling between β_2_AR and LTCC requires CaMKII as an essential element, without excluding PKA involvement. All elements are forming part of a complex that lose its function under pathological conditions.

### The Involvement of Cholesterol and Cav3 Downstream β_2_AR

Cav3 (caveolin-3), the key structural protein of caveolae, is a key factor for the compartmentalisation of β_2_AR signaling in the T-tubules microdomains.^25^ We, therefore, removed caveolae from the surface of cardiomyocytes with methyl-β-cyclodextrin (MβCD)^26,27^ and observed a loss of coupling between LTCC and β_2_AR (Methyl-β-cyclodextrin [MβCD] Po: 0.047±0.007 versus β_2_AR_stimulation_-MβCD Po: 0.044±0.012, Figure 4B).

We then investigated how the removal of cholesterol affects β_2_AR signaling at the global level and understand how the coupling with LTCC was lost. Cells were pretreated with MβCD for 30 minutes and then incubated for an extra 15 minutes with no drugs or with 1 µM ISO+0.3 µM CGP to stimulate β_2_AR. Protein expression of pPLNSer (PKA) and pPLNThr (CaMKII) were detected as explained above (Figure 4C). First of all, we confirmed that MβCD by itself did not produce a change of their expression (Figure 4D and 4E). Interestingly, MβCD treated cells under β_2_AR stimulation produced a 8-fold increase of PKA activity (*P*=0.0286, Figure 4D), a significant increase compared with the lack of response observed in control cells (Figure 2F). MβCD-treated cells did not result in any significant changes on CaMKII under β_2_AR stimulation (Figure 4E).

We further investigated the distribution of βARs and its relationship with Cav3 using immunostaining in control and failing cardiomyocytes (Figure S5A), assessing their degree of co-staining with Mander’s coefficients (M1/M2).^28^ M1 (percentage of total Cav3 that show co-staining with βARs) revealed a higher signal of Cav3 co-stained with β_2_AR than with β_1_AR in control cells (Figure S5B). By contrast, M2 (percentage of total βARs that show co-staining with Cav3) displayed a comparable co-staining of β_1_ARs and β_2_ARs with Cav3 in control group. In failing cells, M1 did not show any significant increase for Cav3 and β_2_AR co-staining and M2 presented a higher β_1_AR co-staining with Cav3 (Figure S5C). Notably, failing cells also presented an increase in signal intensities for β_1_AR (Figure S5E and S5F).

The increased area of the failing cardiomyocytes (Figure S5D), in agreement with our capacitance data (Figure S1), further confirmed the hypertrophic phenotypes of the failing cells.

### Only LTCC Forming a Complex With Cav3 Are Susceptible to the Local β_2_AR Stimulation

**Figure 5.**
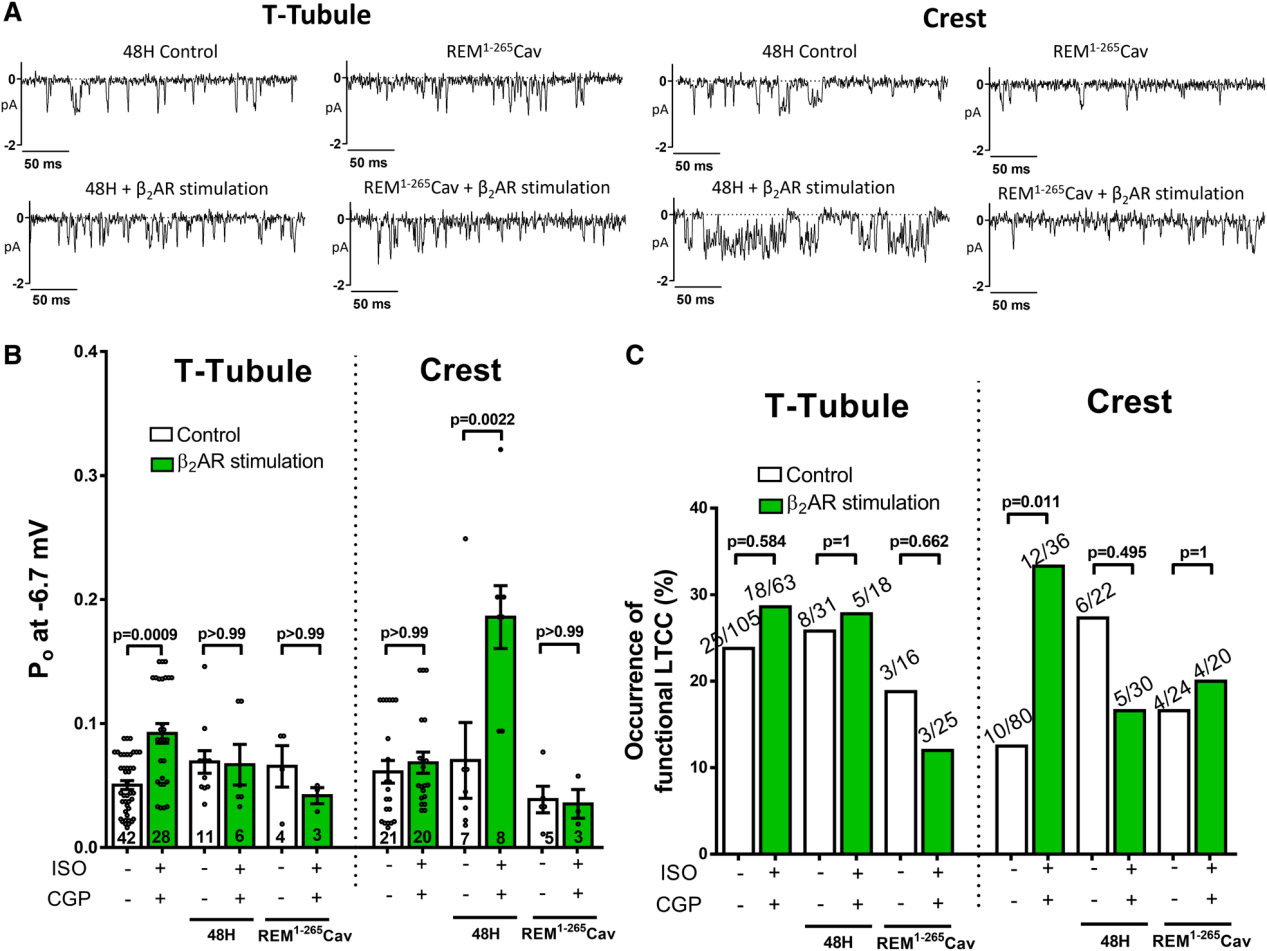
**REM^1-265^Cav blocks the local interaction between L-type Ca^2+^ channel (LTCC) and β_2_AR located in caveolae domains after 48 hours in culture. A**, Representative single LTCC traces at −6.7 mV. **B**, Summary graph of the Po in TT and Crest from control cells, 48 hours culture cells with and without local β_2_AR stimulation, and 48-hour REM^1-265^Cav transfected cells with and without β_2_AR local stimulation (n=channels/cells/animals 42/21/12, 28/15/9, 11/8/4, 6/4/3, 4/3/3, 3/3/3, 21/10/8, 20/13/7, 7/6/3, 8/4/3, 5/4/4, 3/3/1, *P* values by Kruskal-Wallis followed by Dunn multiple comparisons test). **C**, Summary graph of the chance of obtaining a LTCC current (% of occurrence; *P* values by Fisher exact test).

To further investigate the relationship between caveolae domains and the activation of LTCCs by β_2_AR, REM^1-265^Cav peptide^22,27^ was overexpressed in control cardiomyocytes and local β_2_AR stimulation was tested. REM^1-265^Cav is a Cav3-targeted LTCC-blocking agent that blocks exclusively LTCCs located in the caveolae. Since adenoviral overexpression of the peptide required culturing the cells for 48 hours, we tested the effect of local β_2_AR stimulation in these cells. We found that due to subcellular remodeling the local β_2_AR stimulatory effect on LTCC in the TTs was lost (TT_48-hrs_ Po: 0.069±0.009 versus TT_48-hrs_ β_2_AR_stimulation_ Po: 0.067±0.016; Figure 5A and 5B). Similarly, in REM peptide expressing cells, there was no increase in TT LTCC activity after β_2_AR stimulation either (TT-REM Po: 0.066±0.017 versus TT-β_2_AR-_stimulation_-REM Po: 0.042±0.007; Figure 5B). However, in cardiomyocyte after 48-hrs culture, Crest LTCC became susceptible to β_2_AR stimulation (Crest_48-hrs_ Po: 0.07±0.03 versus Crest_48-hrs_ β_2_AR_stimulation_ Po: 0.186±0.025, *P*=0.0022; Figure 5B), and REM^1-265^Cav was clearly blocking this effect on the Crest LTCC (Crest-REM Po: 0.039±0.011 versus Crest-β_2_AR_stimulation_-REM Po: 0.035±0.012; Figure 5B). In general, channels that are noncaveolae located (either in TT or Crest regions), the only channels in the expressing REM^1-265^Cav cells available, did not show any increase after β_2_AR stimulation. We also observed a trend in a reduced chance to record channels in the REM^1-265^Cav cells (Figure 5C). Interestingly, when REM^1-265^Cav was overexpressed in failing cells the occurrence of LTCC in the crest was halved (Figure S6A), and the Po from crest LTCC was reduced to control values (Figure S6B). These data suggest that in failing cells the pathological active channels in the Crest domains are in the caveolae, and they could be linked to β_2_AR.

### Cav3KO Mice Lost the Microdomain Coupling of β_2_AR With LTCC

**Figure 6.**
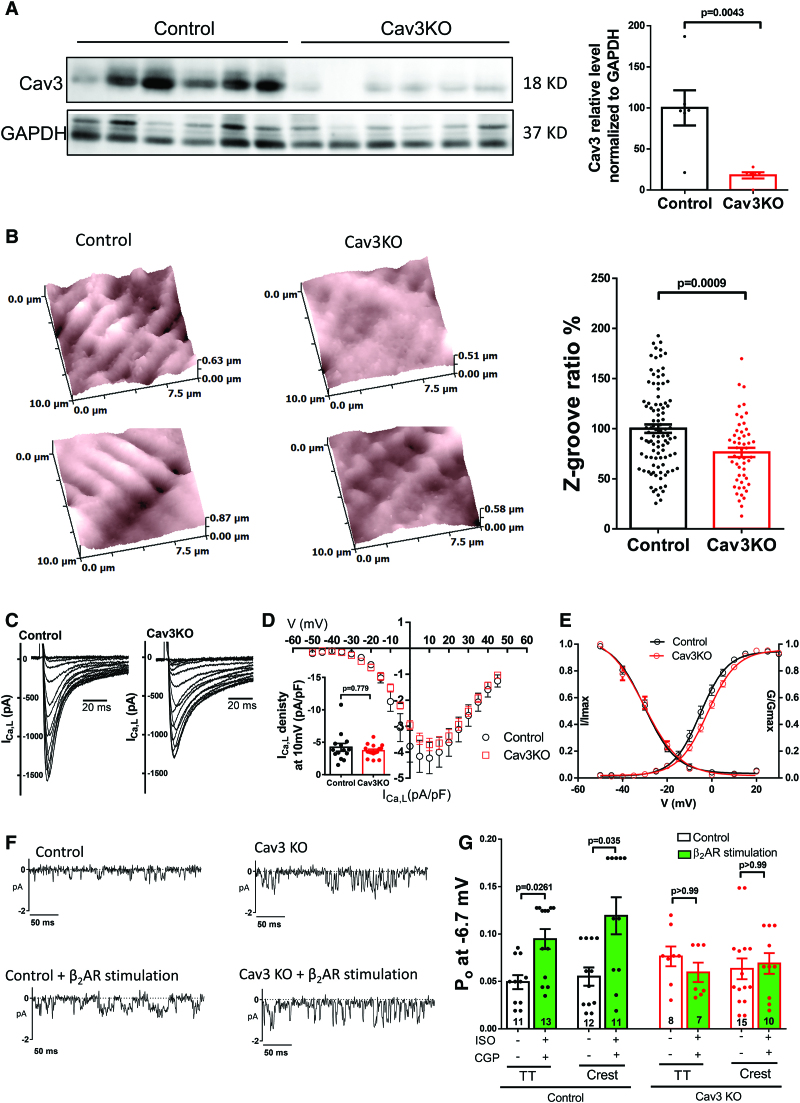
**The local interaction between L-type Ca^2+^ channel (LTCC) and β_2_AR is lost in the tamoxifen-induced Cav3KO mouse model. A**, **Left**, WB of control and Cav3KO mouse cardiomyocytes. **Right**, summary graph of densitometry analysis of cav3 normalized to GAPDH from control (n=6) and Cav3KO (n=6) cardiomyocytes (*P* value by Mann-Whitney *U* test). **B**, **Left**, 10×10 µm representative scanning ion conductance microscopy (SICM) images of control and Cav3KO cardiomyocytes. Right, summary graph showing the Z-groove index as a measure of surface regularity in control (n=99) and Cav3KO (n=52) cardiomyocytes (*P* value by Mann-Whitney *U* test). **C**, Representative traces of I_Ca,L_ currents in control and Cav3KO cardiomyocytes. **D**, I/V summary graph of I_Ca,L_ (Control n=14/4, Cav3KO n=16/4). **E**, I_Ca,L_ activation and inactivation curves in control and Cav3KO cardiomyocytes (Control n=14, Cav3KO n=16). **F**, Representative traces of single LTCC recordings at −6.7 mV. **G**, Summary graph of the Po in TT and Crest from control and Cav3KO cardiomyocytes, with and without local β_2_AR stimulation (n=channels/cells/animals 11/6/4, 13/6/3, 12/6/2, 11/5/3, 8/6/3, 7/5/3, 15/9/4, 10/6/3, *P<0.05, by Kruskal-Wallis followed by Dunn multiple comparisons test). Cav3KO indicates caveolin-3-knockout.

To elucidate the role of caveolae in the complex involving β_2_AR and LTCC, a more direct approach was used: isolating cardiomyocytes from a tamoxifen-treated Cav3KO. The tamoxifen-induced knockout of Cav3 was confirmed with Western blotting of cardiomyocytes pellets: Cav3KO cardiomyocytes expressed 5 times less Cav3 than controls (Cav3KO 17.9±3.8% versus control, *P*=0.0043, Figure 6A). We assessed whether the lack of Cav3 affected the cardiomyocyte morphology, the surface topography was analyzed by scanning ion conductance microscopy calculating the Z-groove index, a measure of surface regularity. It represents a ratio of the measure Z-groove length to the total extrapolated Z-groove length (as if they were present throughout the entire surface).^29^ Z-groove index significantly dropped by 24% in Cav3KO when compared with control (*P*=0.0009, Figure 6B). Whole-cell I_Ca,L_ was recorded in this model, but no difference were observed under baseline conditions between control and Cav3KO cells (Figure 6C through 6E). Finally, single LTCCs were recorded in the TT or Crest microdomain with control solutions or under local β_2_AR stimulation (Figure 6F). In control mouse cells LTCC activity in response to β_2_AR stimulation significantly increased both in TT from 0.049±0.007 to 0.095±0.011 (*P*=0.0261, Figure 6G), and in the Crest 0.055±0.01 to 0.119±0.02 (*P*=0.035, Figure 6G). However, this effect was lost in the Cav3KO group, in which β_2_AR stimulation was unable to increase the Po in both TT and Crest regions (KO Po: 0.077±0.01 versus KO-β_2_AR_stimulation_ Po: 0.06±0.011 in the TT; and KO Po: 0.063±0.011 versus KO-β_2_AR_stimulation_ Po: 0.069±0.0011 in the Crest, Figure 6G). These results confirm similar LTCC behavior in both rats and mouse cardiomyocytes and that caveolae are indeed mediating the local β_2_AR stimulation.

### The LTCC- β_2_AR Coupling Is Present in Healthy Human Cardiomyocytes and Lost in Cardiomyocytes From Dilated Cardiomyopathies

**Figure 7.**
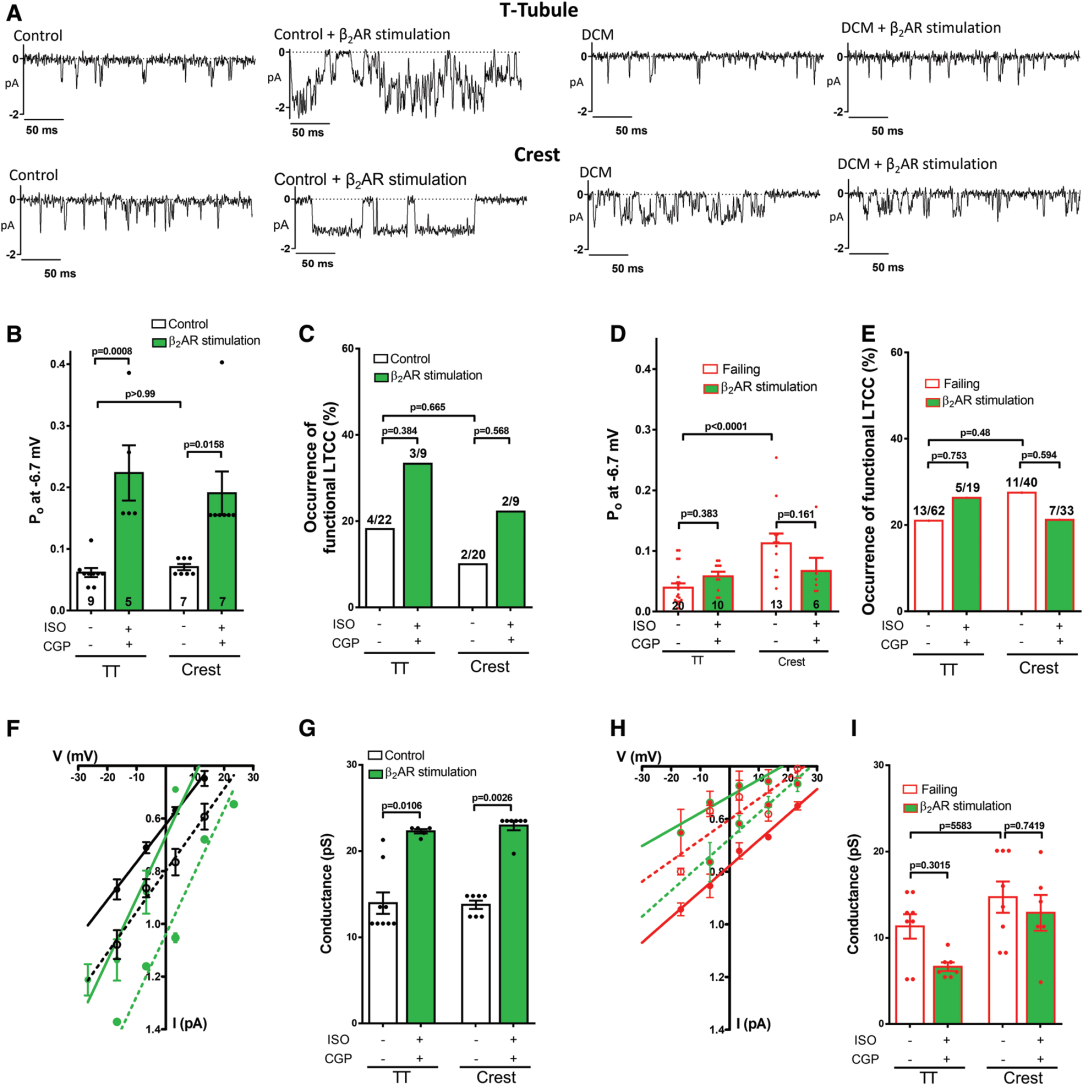
**Local β_2_AR stimulation can increase the Po of L-type Ca^2+^ channel (LTCC) in control human cardiomyocytes, but not in failing cardiomyocytes from patients with dilated cardiomyopathy (DCM). A**, Representative traces of single LTCC recordings at −6.7 mV. **B**, Summary graph of the Po in TT and Crest from human control cardiomyocytes, with and without local β_2_AR stimulation (n=channels/cells/patients 9/4/3, 5/3/1, 7/2/1, 7/2/1, *P<0.05,***P<0.001 by Kruskal-Wallis followed by Dunn multiple comparisons test). **C**, Representation of the chance of obtaining a LTCC current (% of occurrence) in human control cardiomyocytes. **D**, Summary graph of the Po in TT and Crest from patients with DCM, with and without local β_2_AR stimulation (n=channels/cells/patients; 20/13/7, 10/5/2, 13/9/7, 6/5/2, *P<0.05, ***P<0.001 by Kruskal-Wallis followed by Dunn multiple comparisons test). **E**, Percentage of occurrence in failing cardiomyocytes from patients with DCM. **F**, I/V plots of single channel recordings from human control cardiomyocytes. **G**, Summary graph of the conductance analysis obtained from the slope in F (n=channels/cells/patients; 9/4/3, 5/3/1, 7/2/1, 7/2/1, *P<0.05, **P<0.01 by Kruskal-Wallis followed by Dunn multiple comparisons test). **H**, I/V plots of single channel recordings from human failing cardiomyocytes. **I**, Summary graph of the conductance analysis obtained from the slope in H (n=channels/cells/patients; 8/4/4, 7/4/1, 8/5/3, 6/5/2, *P* values by Kruskal-Wallis followed by Dunn multiple comparisons test).

To further validate our rodent models as a preclinical model, we tested if the effect of local β_2_AR stimulation observed in rats and mice cardiomyocytes is also present in human cardiomyocytes from control and dilated cardiomyopathy patients (Figure 7). Although the number of cells recorded was low due to the limited number of human samples, we can confirm that the local β_2_AR stimulation produced a significant increase in both the Po and conductance of the LTCC in cardiomyocytes from control (Figure 7A). The LTCC Po in the TT increased from 0.055±0.006 to 0.223±0.045 (*P*=0.0008) and in the Crest from 0.058±0.005 to 0.190±0.035 (*P*=0.0158, Figure 7B). A modest nonstatistically significant increase of the LTCC occurrence was also observed (Figure 7C). In dilated cardiomyopathy cardiomyocytes, the local β_2_AR stimulation did not elicit a change in either LTCC Po in TT or crest microdomains (Figure 7D and 7E) and did not increase the LTCC conductance (Figure 7F through 7I).

These results confirm that the close functional coupling between β_2_AR and LTCC described in this work seems to be preserved across species, including human cardiomyocytes, and potentially impaired in pathological conditions, illustrating the importance that this coupling might have for a physiological adrenergic response and as a potential target for rescuing therapeutics.

## Discussion

LTCCs play a key role in cardiomyocytes physiology; βARs are among their main regulators. One of the treatments of choice for the management of HF are β-blockers (blockers of βARs),^30^ hence the necessity to fully understand the underlying pathways both in control conditions and in HF and investigate putative selective targets. Here, we examined the modulation of LTCCs upon βARs stimulation, the distinct contribution of β_1_ARs and β_2_ARs, and the kinases involved both at global as well as microdomain levels. Consistent with several publications,^4,31,32^ we observed an increase of I_Ca,L_ upon βARs stimulation, both at whole-cell and microdomain levels. We elucidated 2 distinct contributions. β_1_ARs is involved in the global increase of I_Ca,L_ with a major role of a PKA-dependent pathway. By contrast, a CaMKII-dependent signaling downstream β_2_ARs is involved in the local increase of LTCC current at specific microdomains, for example, in the T-tubular regions of cardiomyocytes.

**Figure 8.**
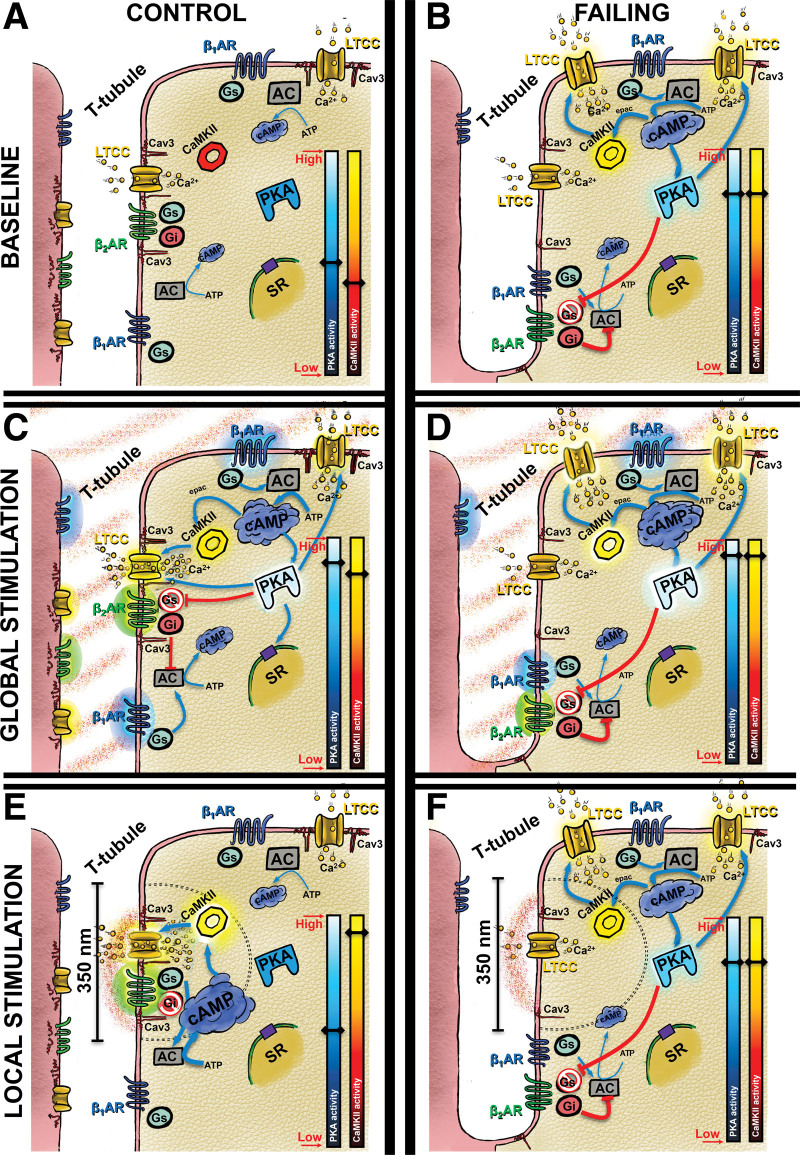
**Schematic representation of the proposed signaling mechanism between L-type Ca^2+^ channel (LTCC) and β_2_AR. A**, Normal distribution of LTCC and βAR on the T-tubule and Crest microdomain. **B**, On failing cells, the loss of TTs redistributed LTCCs, increasing their presence on the crest. Multiple targets proteins are phosphorylated at baseline, due to the higher activity of PKA and CaMKII (Ca^2+^/calmodulin-dependent protein kinase II) as a compensatory mechanism for the reduction in function. Including LTCC on the crest and β_2_AR, which switch to the inhibitory Gi subunit. **C**, General catecholamine stimulation will preferably affect control cardiomyocytes via beta-1 adrenergic receptors (β_1_ARs), because of the higher expression than β_2_ARs. This will cause a global cytosolic increase of cAMP, and consequently a global activation of PKA and CaMKII. Increasing the activity of LTCCs. PKA will also phosphorylated β_2_AR, switching to Gi, working as negative feedback. **D**, On failing cells, a global stimulation will cause a small response, as PKA and CaMKII activity cannot be increased much further. Observed as a blunted response of LTCC activity. **E**, In healthy conditions, β_2_ARs signaling is restricted to the local environment., When the stimulation happens close to a LTCC in a very specific area (<350 nm), only the local β_2_ARs will be activated. Activating a restricted signaling. A specific pool of cAMP that activated CaMKII, phosphorylating LTCC only on that local microdomain. **F**, However, due to the lack of LTCC-β_2_AR complexes on failing cells, local catecholamine stimulation will not have any effect on these cells. βAR indicates beta-adrenergic receptor; cAMP, cyclic adenosine monophosphate; PKA, protein kinase A; and TT, T-tubule.

We found that the microdomain coupling between β_2_AR and LTCC is conserved across rodents (rats and mice) and between rodents and humans; in both cases blunted in failing cells, further validating our rat model to study heart failure. Notably, consistent with previous reports studying disease models,^1,2,33^ the reduced response in failing cells could probably be explained by the higher levels of β_2_ARs phosphorylation on those cells. Moreover, this conserved coupling between LTCC and β_2_AR requires the presence of Cav3, and intact caveolae to take place. A diagram summarizing the mechanism that we suggest in this work, is represented in Figure 8.

### Global β_1_AR Stimulation Increases PKA Activity and LTCC Po, a Response That Is Blunted in Failing Cardiomyocytes

We aimed to investigate the regulation of single LTCC in TT and Crest microdomains upon βARs stimulation. Using scanning ion conductance microscopy smart patch-clamp,^34^ we showed that βARs stimulation increased the Po of LTCC from control rat cardiomyocytes, independently of the microdomain (Figure 1). By WBs, we also confirmed that the treatment with ISO led to a significant increase on pPLNSer and pPLNThr expression levels, which indicated increased PKA and CaMKII activity, respectively (Figure 2). Global β_1_AR stimulation alone resulted in a significant increase of pPLNSer expression, but not of pPLNThr and a significant increase in whole-cell I_Ca,L_ current. By contrast, global β_2_AR stimulation did not produce any significant changes of PKA or CaMKII activity nor any significant effects on whole-cell I_Ca,L_ currents. This is consistent with some previous report demonstrating a lack of stimulatory effect upon global β_2_AR stimulation in mouse ventricular cardiomyocytes.^24^ This pathway, studied for decades, represents the classic response of cardiomyocytes to adrenergic stimulation from sympathetic neurons, in which the release of norepinephrine will produce an ionotropic and inotropic effect on cardiomyocytes.^35,36^ We demonstrated that the PKA-dependent pathway acts downstream β_1_AR, and results in the increase of I_Ca,L_.

Interestingly, we found that in our 16-week MI rat model the effect of βAR on LTCC was blunted. In failing cells, ISO did not increase LTCC Po in the microdomains and resulted in a dramatically reduced response in both whole-cell ICaL and PKA activity (Figures 1 and 2). Notably, βAR dysfunction is closely related to the development of HF and disease:, for example, cytoarchitectural changes that occur in HF affect the distribution and function of βARs.^7^ LTCCs have also been proposed as a key element in the development of HF,^37–39^ and we previously showed how their activity and distribution could lead to whole heart arrhythmias.^2,40^

### LTCCs Are Coupled With β_2_AR but Not With β_1_AR, an Interaction That Is Disrupted in the TT of Failing Cardiomyocytes

Local stimulation of LTCC by βARs was described by Chen-Izu et al^4^ under classic cell-attached recordings. They showed how β_1_AR activates LTCC independently of the distance to the channel, but the action of β_2_AR requires vicinity to LTCCs. In accordance with their study, we observed a similar response from LTCCs by local β_2_AR stimulation. However, in contrast to the results from Chen-Izu and co-workers, we found a lack of response following β_1_AR stimulation at the single channel level. These different results might be explained by the differences in the experimental protocol: the seal is significantly smaller using our smart patch-clamp setup compared to the classic cell-attached configuration. Two hypotheses can support our results. The first is that the lack of β_1_AR response may be due to the absence of this receptor under the area of recording. β_1_ARs are evenly distributed on the surface of the cell membrane,^7^ and they are not associated with LTCC,^5,6^ as a result, it is possible that there is a low chance that β_1_AR close enough to LTCC in our conditions, an area of seal of <0.160 µm^2^. The second hypothesis indicates that the local β_1_AR stimulation is not sufficient to generate enough cAMP to elicit a PKA-dependent pathway in the nearby LTCCs, perhaps the number of receptors under the area recorded is too low.

Interestingly, we observed an increase in LTCC occurrence after β_2_AR stimulation (Figures 3 and 7) or in other words, the number of channels that are active in the cardiomyocyte membrane. This could be explained by the enhancement of Po itself which will increase the chance to record the channels. Another explanation could be related to the mobilization of a subsarcolemma pool of LTCC to the area of βAR-stimulation as shown by Dixon group^41,42^ with our data suggesting that β_2_AR are responsible for the insertions of LTCC.

On the other hand, experiments on failing cells confirmed previously published observations: the response to adrenergic stimulation is reduced in HF^11^ with a decrease of both CaMKII and PKA response downstream βARs (Figure 1). The local coupling between β_2_AR and LTCC observed in control cells is lost in failing cells, probably due to a disruption of the complex between the 2 proteins. The impaired compartmentalization of β_2_AR and a redistribution of its action from local to global β_2_AR signaling,^7,43^ could explain the disruption of the this functional complex in failing cells. This is in agreement with previous studies: β_2_ARs and their signaling are no longer confined in the TT and redistributed in the plasma membrane, including the crest regions.^7^ Moreover, our experiments blocking β_2_AR in the crest with ICI (Figure S4B) resulted in a reduction of LTCC Po, suggesting the presence of constitutive active β_2_AR, possibly leading to a pathological increase in LTCC activity. Furthermore, in HF, the raise in circulating catecholamines results in hyperstimulation of β_1_ARs, leading to their downregulation and β-arrestin–dependent internalization. β-arrestin binding to β_1_ARs results into the uncoupling of G_s_ from β_2_ARs, which together with the GRK2-phosphorylation of β_2_ARs results into enhanced G_i_-biased signaling downstream β_2_ARs, the activation of CaMKII pathways and the further uncoupling of G_s_ from β_1_ARs.^30^ In accordance with the G_i_-biased signaling in HF, there are some promising data of a combinational therapy for HF employing β_1_ARs selective blockers and a G_s_-biased β_2_ARs agonist. The block of β_1_ARs overstimulation by catecholamines, and the stimulation of G_s_-β_2_ARs signaling could restore the contractility of the failing heart.^30,44,45^

We would like to highlight the novelty of our approach to βAR activation by locally applying agonist only to a small area of the membrane in which only a small number of LTCC/ β_2_AR complexes could be located.

Our study provides functional evidence of the specific coupling of β_2_AR with LTCCs that is dependent on the location of the channels and is disrupted in failing cardiomyocytes.

### The Mechanism of β_2_AR-LTCC Coupling Requires CaMKII Activity in Their Local Environment

From a classical perspective, the inotropic effect of β_2_AR activation, and the subsequent increase of LTCC activity, has been widely related to an increase in cytosolic cAMP concentration and PKA activity.^46,47^ However, a cAMP-independent effect of βAR on LTCC activity had been also suggested,^48,49^ although it has never been extensively corroborated. Here, we illustrate that the stimulation of βARs results into a general cAMP-PKA pathway, which can be seen in the whole-cell experiments under ISO stimulation (Figures 1 and 2), and a distinct secondary pathway at the microdomain level. It has been shown in neurons that β_2_AR can be stimulated by a broad distribution of cAMP-PKA after ISO and that β_2_AR can be activated in the immediate vicinity of the receptor by carvadiol or alprenolol.^50^ In our case, we show that β_2_AR and LTCC are part of the same functional complex, expressed in caveolae domains. This complex requires the activity of CaMKII in the local region for its function to take place. In fact, when CaMKII is blocked, the interaction between β_2_AR and LTCC can no longer take place (Figure 4). CaMKII is involved in a variety of cellular processes, including cell growth or hypertrophy,^51^ and it has been proposed as a good candidate for HF treatments.^21^ We demonstrate that CaMKII in the close environment of the β_2_AR-LTCC complex plays a crucial role, which could be exacerbated in the Crest domains of MI cells, in which the pathologically active LTCC can be controlled by blocking CaMKII (Figure S4). Nevertheless, the involvement of PKA-dependent signaling cannot be excluded from this complex. We did not observe an increase in PKA after β_2_AR stimulation by WB, but this does not imply that PKA could not be locally active near the microdomain, as our data showed a potential contribution (Figure 4B). In fact, we have previously shown how disruption in the β_2_AR-cAMP signaling and LTCC activity could be linked.^52^ Further studies, for example, by using a transgenic approach,^18^ could reveal with more precision the mechanism of this interaction.

### Cav3 Plays a Fundamental Role in the β_2_AR-LTCC Complex

Caveolae are small invaginations of the plasma membrane (50–100 nm in diameter) that can be found along the surface of cardiomyocytes. They work as signalosomes of specific proteins, that use this enriched cholesterol domains to interact between them and produce local effects on the cells.^53,54^ Caveolae play a fundamental role in the regulation of βAR signaling, from neonatal^55^ to adult cardiomyocytes.^56^ We demonstrated that when cholesterol is removed by MβCD, and consequently caveolae domains are disrupted,^57^ the local coupling between β_2_AR and LTCC is lost (Figure 4). Interestingly, our WB results suggest that β_2_AR stimulation after cholesterol depletion reveal a β_1_AR-like phenotype. We propose that the disruption of the caveolae complex is affecting the β_2_AR signaling pathway, like the effect that can be observed in HF, and probably is linked to changes in the G_s_/G_i_ ratio. We also blocked the LTCC pool specifically associated with caveolae, by overexpressing REM^1-265^Cav in healthy cells.^22,27^ As expected, this treatment blocked the interaction of β_2_AR with LTCC, as the remaining nonblocked LTCC did not respond to β_2_AR stimulation (Figure 5).

The main protein required to form caveolae in cardiomyocytes is Cav3,^54^ which has been widely associated with the regulation of β_2_AR.^3,25,55,58^ Cav3 plays a key role in the compartmentalisation of cAMP signals in the TT domains upon β_2_AR activation. Accordingly, if Cav3 is disrupted in healthy cardiomyocytes, stimulation of β_2_ARs results into a similar effect to the one observed in HF.^25^ Even more importantly, reintroduction of Cav3 in failing rabbit cardiomyocytes can normalize the β_2_AR signal and restore the contractile response to adrenergic stimulation.^59^ Co-immunostaining of control cardiomyocytes confirm that the total percentage of Cav3 that is co-stained with β_2_AR is higher (47%) than with β_1_AR (39%; Figure S5B). These results demonstrate a remarkable preference of Cav3 toward β_2_AR, it is in fact, important to note the higher abundancy of β_1_ARs in cardiomyocytes, (4:1. β_1_Ars:β_2_ARs expression ratio).^30^

In Cav3^−/−^ mice, the T-tubules and I_Ca,L_ density is decreased.^60^ Here, by using a conditional Cav3-KO mouse, we can confirm that removing Cav3 causes changes in the topographical surface, decreasing the Z-groove index (Figure 6). The lack of Cav3 also disrupts the functional complex between LTCC and β_2_AR. This demonstrates the importance that Cav3 have in the compartmentalization and regulation of β_2_AR signaling, and its close relationship with LTCC.

The same local functional complex between β_2_AR and LTCC is observed in isolated human cardiomyocytes (Figure 7). Under local stimulation, LTCC can be activated by β_2_AR too, and cardiomyocytes isolated from dilated cardiomyopathy samples did not present this effect, similarly to the results obtained with the 16-week MI rat model. Although caution needs to be exercised in the interpretation of human results, due to the low number of samples. That this complex is preserved in different species suggests how critical it can be for the physiological response to adrenergic stimulation. β_2_AR modulation of LTCC is considered to be a contributing factor of the cardiac pathological phenotype.^23^

## Conclusions

β_2_AR, but not β_1_AR, can regulate the local environment of LTCCs by forming a complex that requires Cav3. This precise mechanism could tune the LTCC response to adrenergic stimulation. This work helps to understand the strict control that occurs at the microdomain structures of healthy cardiomyocytes, and the importance of therapies searching to recover this local mechanism that is lost in heart failure.

## Article Information

### Acknowledgments

The authors thank Mr Peter O’Gara for isolation of rat cardiomyocytes. We are thankful to the Facility for Imaging by Light Microscopy (FILM) at Imperial College London. Human HF tissue provided with the support of Cardiovascular Biomedical Research Unit at Royal Brompton and Harefield NHS Trust. Donor samples were provided with the support of National Health Service Blood and Transplant (NHSBT).

### Sources of Funding

This work was supported by British Heart Foundation (grant RG/17/13/33173 to Dr Gorelik, grant PG/19/13/34247C to Dr Brand/Dr Gorelik, and grant RG/F/22/110081 to Dr Gorelik/Dr Sanchez-Alonso).

### Disclosures

None.

### Supplemental Material

Expanded Material and Methods

References 61,62

Figures S1–S6

Tables S1–S5

Data set

Unedited WB

Mayor Resource Table

## Supplementary Material
