# Supplementary material for: Functional LTCC-β2AR Complex Needs Caveolin-3 and Is Disrupted in Heart Failure
Source: Circ Res. 2023 Jun 14;133(2):120–37. doi: 10.1161/CIRCRESAHA.123.322508 (PMC10321517; doi:10.1161/CIRCRESAHA.123.322508)
Supplement: Supplementary file 1 [file res-133-120-s001.pdf]

FIGURE 1C

	**FIGURE 1C**										
	Control						Failing				
	TT			Crest			TT			Crest	
	Baseline	ISO		Baseline	ISO		Baseline	ISO		Baseline	ISO
	0.039	0.039		0.039	0.072		0.05	0.073		0.0374	0.043
	0.064	0.078		0.039	0.072		0.067	0.084		0.138	0.14
	0.064	0.078		0.031	0.063		0.067	0.084		0.138	0.14
	0.037	0.05		0.031	0.063		0.039	0.019		0.068	0.107
	0.037	0.05		0.071	0.135		0.039	0.019		0.085	0.084
	0.037	0.05		0.119	0.137		0.114	0.088		0.085	0.084
	0.075	0.08		0.119	0.137		0.011	0.016		0.132	0.191
	0.075	0.08		0.119	0.137		0.011	0.016		0.132	0.191
	0.075	0.08		0.119	0.137					0.132	0.191
	0.075	0.08		0.119	0.137					0.132	0.191
	0.021	0.036		0.119	0.137					0.132	0.191
	0.061	0.041		0.053	0.075					0.127	0.096
	0.061	0.041		0.053	0.075					0.098	0.111
	0.077	0.134		0.0197	0.0356					0.098	0.111
	0.077	0.134		0.0197	0.0356					0.098	0.111
	0.077	0.134		0.0197	0.0356						
	0.077	0.134		0.0879	0.092						
	0.077	0.134									
	0.064	0.111									
	0.064	0.111									
	0.064	0.111									
No of Channels	21			17			8			15	
No of Cells	8			7			5			7	
No of Animals	6			5			4			7	
**Statistical test on No of Channels**											
**Wilcoxon matched-pairs signed rank test**											
P value	0.0012			< 0.0001			0.9063			0.0042	
Exact or approximate P value?	Exact			Exact			Exact			Exact	
P value summary	**			****			ns			**	
Significantly different? (P < 0.05)	Yes			Yes			No			Yes	
One- or two-tailed P value?	Two-tailed			Two-tailed			Two-tailed			Two-tailed	
Sum of positive, negative ranks	187.0 , -23.00			153.0 , 0.0			17.00 , -19.00			108.0 , -12.00	
Sum of signed ranks (W)	164			153			-2			96	
Median of differences											
Median	0.014			0.018			0.005			0.013	
How effective was the pairing?											
rs (Spearman)	0.8864			0.9974			1			0.8127	
P value (one tailed)	< 0.0001			< 0.0001			0.0002			0.0002	
P value summary	****			****			***			***	
Significant correlation? (P > 0.05)	No			No			No			No	

FIGURE 1E

FIGURE 1E							
I/V Curve					Inset Control Vs ISO at 0mV		
mV	ctrl baseline	SEM	ctrl ISO	SEM		Baseline	ISO
-50	-0.2747101	0.050192	-0.35157	0.046662		-2.43964	-6.71892
-45	-0.1698834	0.024831	-0.19727	0.022595		-3.64191	-5.87525
-40	-0.07448895	0.007257	-0.07202	0.007084		-3.24926	-6.86758
-35	-0.0940027	0.010741	-0.12376	0.013641		-3.26583	-5.07925
-30	-0.2087533	0.031258	-0.408	0.044972		-4.65916	-6.14457
-25	-0.6823123	0.150052	-1.43021	0.153664		-4.1733	-3.80276
-20	-1.778064	0.418785	-3.19266	0.363868		-4.94923	-7.76298
-15	-2.866523	0.513211	-4.55467	0.374778		-9.04172	-7.47669
-10	-3.777212	0.502658	-5.46916	0.400741		-4.18982	-4.56805
-5	-4.357305	0.49208	-5.81525	0.412921		-7.68395	-8.98958
0	-4.562407	0.483487	-5.85349	0.436654		-2.73622	-5.9401
5	-4.523961	0.443576	-5.58446	0.436976		-6.05981	-7.25705
10	-4.293352	0.389539	-5.16793	0.428242		-1.37599	-1.61833
15	-3.88441	0.356252	-4.58983	0.406684		-4.94703	-5.31708
20	-3.419447	0.317587	-3.96877	0.365339		-5.02789	-4.97238
25	-2.943043	0.289699	-3.29951	0.324175		-5.55776	-5.26526
30	-2.371463	0.245281	-2.6863	0.276477			
35	-1.842243	0.204742	-2.10267	0.226959			
40	-1.391627	0.168365	-1.64402	0.207381			
45	-1.003839	0.135197	-1.2344	0.166641			
					No of Cells	16	
					No of Animals	5	
		**Statistical test on No of cells**					
		**Wilcoxon matched-pairs signed rank test**					
			P value			0.0092	
			Exact or approximate P value?			Exact	
			P value summary			**	
			Significantly different? (P < 0.05)			Yes	
			One- or two-tailed P value?			Two-tailed	
			Sum of positive, negative ranks			19.00 , -117.0	
			Sum of signed ranks (W)			-98	
			Median of differences				
			Median			-1.251	
			How effective was the pairing?				
			rs (Spearman)			0.4412	
			P value (one tailed)			0.0445	
			P value summary			*	
			Significant correlation? (P > 0.05)			No	

FIGURE 1G

FIGURE 1G

I/V Curve					Inset Failing Vs ISO at 0mV		
mV	Failing baseline	SEM	Failing ISO	SEM		Baseline	ISO
-50	-0.2749481	0.026143	-0.2781242	0.027724		-3.75433	-5.40513
-45	-0.1526616	0.013968	-0.1633572	0.015513		-5.83698	-5.0695
-40	-0.04856964	0.002987	-0.05207797	0.004387		-3.99294	-3.49385
-35	-0.1033532	0.00599	-0.1313518	0.012975		-4.79179	-6.29965
-30	-0.3296319	0.02375	-0.472468	0.053512		-1.45699	-2.95209
-25	-0.8599956	0.068565	-1.228106	0.120322		-5.0578	-7.63753
-20	-1.705498	0.161241	-2.320845	0.226066		-3.0697	-3.52296
-15	-2.558802	0.205531	-3.255645	0.371		-5.53698	-5.83678
-10	-2.959006	0.24813	-3.756595	0.430729		-3.89646	-3.62293
-5	-3.526261	0.315247	-4.000794	0.439371		-1.61329	-1.92111
0	-3.578933	0.340558	-3.98983	0.427767		-3.18315	-2.99379
5	-3.6026	0.309655	-3.799424	0.41717		-2.44197	-2.60699
10	-3.430686	0.339226	-3.435995	0.400127		-3.46842	-3.19706
15	-3.183694	0.313375	-3.101941	0.361658		-2.53184	-2.86263
20	-2.917711	0.258992	-2.684051	0.329565		-3.05136	-2.42544
25	-2.455226	0.229149	-2.298103	0.295531			
30	-2.046779	0.215689	-1.875588	0.254016			
35	-1.665534	0.182006	-1.503484	0.206789			
40	-1.240756	0.151666	-1.147328	0.171554			
45	-0.8817681	0.108962	-0.8660513	0.134944			
					No of Cells	15	
					No of Anim	4	

**Statistical test on No of cells**	
**Wilcoxon matched-pairs signed rank test**	
P value	0.2524
Exact or approximate P value?	Exact
P value summary	ns
Significantly different? (P < 0.05)	No
One- or two-tailed P value?	Two-tailed
Sum of positive, negative ranks	39.00 , -81.00
Sum of signed ranks (W)	-42
Median of differences	
Median	-0.2998
How effective was the pairing?	
rs (Spearman)	0.8571
P value (one tailed)	< 0.0001
P value summary	****
Significant correlation? (P > 0.05)	No

FIGURE 2B & 2C

	**FIGURE 2B**					**FIGURE 2C**			
	Control	ISO	Failing	ISO		Control	ISO	Failing	ISO
	1	45.94388	1	3.545267		1	0.964002	1	0.74142
	1	29.14866	1	11.49895		1	1.118182	1	1.088638
	1	1.042858	1	9.074969		1	1.447164	1	1.628182
	1	148.3239	1	5.07422		1	1.235442	1	1.299065
	1	3.109713				1	2.03884		
	1	4.523015				1	1.652568		
	1	18.22593							
	1	140.0594							
No of animals	8		4			6		4	
**Statistical test on No of animals**									
**Mann Whitney test**									
P value	0.0002		0.0286			0.0476		0.3143	
Exact or approximate P value?	Exact		Exact			Exact		Exact	
P value summary	***		*			*		ns	
Significantly different? (P < 0.05)	Yes		Yes			Yes		No	
One- or two-tailed P value?	Two-tailed		Two-tailed			Two-tailed		Two-tailed	
Sum of ranks in column A,B	36.00 , 100.0		10.00 , 26.00			27.00 , 51.00		14.00 , 22.00	
Mann-Whitney U	0		0			6		4	
Difference between medians									
Median of column A	1		1			1		1	
Median of column B	23.69		7.075			1.341		1.194	
Difference: Actual	22.69		6.075			0.3413		0.1939	
Difference: Hodges-Lehmann	22.69		6.075			0.3413		0.1939	

FIGURE 2E

FIGURE 2E					
Control	ß1 stim.	ß2 stim.			
1	93.04408	3.744152			
1	12.78966	0.4746077			
1	5.578064	0.1670716			
1	37.73075	1.690904			
1	108.0022	9.251334			
1	42.90412	1.275383			
No of animals	6				
**Statistical test on No of animals**					
**Kruskal-Wallis test**					
P value	0.0004				
Exact or approximate P value?	Exact				
P value summary	***				
Do the medians vary signif. (P < 0.05)	Yes				
Number of groups	3				
Kruskal-Wallis statistic	11.66				
Data summary					
Number of treatments (columns)	3				
Number of values (total)	18				
Number of families	1				
Number of comparisons per family	3				
Alpha	0.05				
Dunn's multiple comparisons test	Mean rank diff.	Significant?	P value		
Control vs. ß1 stim.	-9.833	Yes	0.0035		
Control vs. ß2 stim.	-2.167	No	>0.9999		
ß1 stim. vs. ß2 stim.	7.667	Yes	0.0339		
Test details	Mean rank 1	Mean rank 2	Mean rank diff.	n1	n2
Control vs. ß1 stim.	5.5	15.33	-9.833	6	6
Control vs. ß2 stim.	5.5	7.667	-2.167	6	6
ß1 stim. vs. ß2 stim.	15.33	7.667	7.667	6	6

FIGURE 2F

FIGURE 2F					
Control	ß1 stim.	ß2 stim.			
1	1.102436	1.164475			
1	0.824727	0.993373			
1	0.747529	1.021431			
1	1.131388	0.959095			
1	1.853011	0.781836			
No of animals	5				
**Statistical test on No of animals**					
**Kruskal-Wallis test**					
P value	0.8675				
Exact or approximate P value?	Exact				
P value summary	ns				
Do the medians vary signif. (P < 0.05)	No				
Number of groups	3				
Kruskal-Wallis statistic	0.3319				
Data summary					
Number of treatments (columns)	3				
Number of values (total)	15				
Number of families	1				
Number of comparisons per family	3				
Alpha	0.05				
Dunn's multiple comparisons test	Mean rank diff.	Significant?	P value		
Control vs. ß1 stim.	-0.8	No	>0.9999		
Control vs. ß2 stim.	0.8	No	>0.9999		
ß1 stim. vs. ß2 stim.	1.6	No	>0.9999		
Test details	Mean rank 1	Mean rank 2	Mean rank diff.	n1	n2
Control vs. ß1 stim.	8	8.8	-0.8	5	5
Control vs. ß2 stim.	8	7.2	0.8	5	5
ß1 stim. vs. ß2 stim.	8.8	7.2	1.6	5	5

FIGURE 2I

FIGURE 2I							
I/V Curve					Inset Control Vs ISO at -15 mV		
mV	ctrl baseline	SEM	ctrl ICI+ ISO	SEM		Baseline	ICI+ISO
-50	-0.282068	0.051825	-0.4099069	0.069387		-1.79079	-4.964934
-45	-0.1734715	0.027694	-0.2259625	0.023279		-2.69987	-5.994572
-40	-0.0639108	0.006328	-0.1240098	0.045144		-6.17246	-8.44744
-35	-0.1466426	0.045948	-0.5640848	0.336556		-2.06722	-10.11793
-30	-0.3918008	0.190046	-3.31546	1.895778		-5.80747	-11.29762
-25	-1.049523	0.50115	-5.540638	1.763849		-0.96735	-4.897374
-20	-2.189764	0.776922	-7.199727	1.34599			
-15	-3.250859	0.896713	-7.619977	1.118828			
-10	-3.963252	0.84867	-7.340165	0.911578			
-5	-4.223255	0.68408	-6.8243	0.858853			
0	-4.336108	0.652216	-6.156107	0.671045			
5	-4.028463	0.554429	-5.660551	0.616258			
10	-3.632648	0.442673	-4.901641	0.491941			
15	-3.195111	0.421489	-4.252635	0.457504			
20	-2.735794	0.346	-3.610434	0.384109			
25	-2.330969	0.362254	-2.814187	0.293951			
30	-1.850662	0.295749	-2.270727	0.270589			
35	-1.482445	0.289027	-1.806246	0.224372			
40	-1.09878	0.228792	-1.338227	0.16687			
45	-0.8118026	0.198836	-1.0275	0.15691			
				No of Cells		6	
				No of Animals		3	
		**Statistical test on No of cells**					
		**Wilcoxon matched-pairs signed rank test**					
		P value				0.0313	
		Exact or approximate P value?				Exact	
		P value summary				*	
		Significantly different? (P < 0.05)				Yes	
		One- or two-tailed P value?				Two-tailed	
		Sum of positive, negative ranks				0.0 , -21.00	
		Sum of signed ranks (W)				-21	
		Median of differences					
		Median				-3.612	
		How effective was the pairing?					
		rs (Spearman)				0.7143	
		P value (one tailed)				0.0681	
		P value summary				ns	
		Significant correlation? (P > 0.05)				Yes	

FIGURE 2J

FIGURE 2J							
I/V Curve					Inset Control Vs ISO at -15 mV		
	ctrl baseli	SEM	ctrl CGP+I	SEM		Baseline	CGP+ISO
-50	-0.13697	0.024494	-0.36868	0.086747		-1.18777	-1.76637
-45	-0.09108	0.016488	-0.21396	0.054343		-1.89905	-1.92022
-40	-0.07722	0.007979	-0.09642	0.0066		-4.19114	-4.17593
-35	-0.10991	0.011204	-0.2008	0.033072		-2.46801	-7.548
-30	-0.29482	0.060681	-0.62145	0.087917		-3.75056	-4.3845
-25	-0.88539	0.215907	-1.99351	0.309547		-5.30805	-5.29616
-20	-1.92556	0.436791	-3.54392	0.746763			
-15	-3.13409	0.631945	-4.18186	0.886252			
-10	-4.03394	0.713965	-4.34593	0.909169			
-5	-4.40045	0.832396	-4.22161	0.882587			
0	-4.52812	0.820458	-3.92647	0.841337			
5	-4.28284	0.807491	-3.4842	0.766146			
10	-3.91306	0.791369	-3.01976	0.683592			
15	-3.48619	0.707794	-2.50693	0.583429			
20	-2.93834	0.637817	-2.01299	0.486127			
25	-2.39556	0.536406	-1.60245	0.399914			
30	-1.95928	0.43733	-1.2365	0.326487			
35	-1.5149	0.345111	-0.95212	0.262432			
40	-1.14069	0.276122	-0.72168	0.201639			
45	-0.80313	0.223952	-0.53009	0.154206			

No of Cells	6
No of Animals	3
**Statistical test on No of cells**	
**Wilcoxon matched-pairs signed rank test**	
P value	0.1563
Exact or approximate P value?	Exact
P value summary	ns
Significantly different? (P < 0.05)	No
One- or two-tailed P value?	Two-tailed
Sum of positive, negative ranks	3.000 , -18.00
Sum of signed ranks (W)	-15
Median of differences	
Median	-0.2999
How effective was the pairing?	
rs (Spearman)	0.6
P value (one tailed)	0.1208
P value summary	ns
Significant correlation? (P > 0.05)	Yes

FIGURE 3A

FIGURE 3A							
I/V Curve					Inset H89 Vs ISO at -5 mV		
mV	H89	SEM	H89+ISO	SEM		H89	H89+ISO
-50	-0.44294	0.131844	-0.36846	0.06305		-5.41826	-5.89898
-45	-0.2397	0.064144	-0.20027	0.030235		-2.44024	-2.54066
-40	-0.08882	0.016276	-0.09932	0.009415		-4.15117	-5.44305
-35	-0.18133	0.03726	-0.29295	0.06273		-3.82623	-4.02004
-30	-0.60536	0.150585	-1.48744	0.555292		-6.0397	-4.71493
-25	-1.57379	0.452046	-2.75667	0.820384			
-20	-2.71168	0.661949	-3.41264	0.629595			
-15	-3.48799	0.634526	-4.32973	0.744629			
-10	-3.9408	0.547582	-4.61915	0.689026			
-5	-4.37512	0.630643	-4.52353	0.589885			
0	-4.38672	0.59425	-4.40844	0.612022			
5	-4.19952	0.610776	-4.06884	0.603667			
10	-3.66881	0.545304	-3.59125	0.567146			
15	-3.30643	0.518018	-3.01357	0.485107			
20	-2.66017	0.423026	-2.51534	0.415588			
25	-2.2823	0.418783	-2.09372	0.344693			
30	-1.81224	0.358237	-1.64719	0.281293			
35	-1.32775	0.25899	-1.26592	0.223378			
40	-1.00722	0.196182	-0.96665	0.187982			
45	-0.72048	0.1341	-0.72735	0.156303			
				No of Cells		5	
				No of Animals		3	
		**Statistical test on No of cells**					
		**Wilcoxon matched-pairs signed rank test**					
		P value				0.625	
		Exact or approximate P value?				Exact	
		P value summary				ns	
		Significantly different? (P < 0.05)				No	
		One- or two-tailed P value?				Two-tailed	
		Sum of positive, negative ranks				5.000 , -10.00	
		Sum of signed ranks (W)				-5	
		Median of differences					
		Median				-0.1938	
		How effective was the pairing?					
		rs (Spearman)				0.7	
		P value (one tailed)				0.1167	
		P value summary				ns	
		Significant correlation? (P > 0.05)				Yes	

FIGURE 3B

FIGURE 3B

I/V Curve					Inset KN93 Vs ISO at -5 mV		
mV	KN93	SEM	KN93+ISO	SEM		KN93	KN93+ISO
-50	-0.28673	0.056642	-0.28048	0.017737		-2.32017	-3.72732
-45	-0.16277	0.032611	-0.18298	0.008619		-2.10009	-2.35065
-40	-0.08921	0.012506	-0.1045	0.012647		-1.23211	-5.74996
-35	-0.21147	0.050377	-0.28528	0.070086		-2.72906	-3.47757
-30	-0.57071	0.178225	-0.80925	0.185216		-3.30387	-3.49738
-25	-1.11102	0.290321	-1.71394	0.183115			
-20	-1.6799	0.304309	-2.69298	0.130087			
-15	-2.07064	0.299713	-3.39899	0.321227			
-10	-2.30013	0.318898	-3.70783	0.479931			
-5	-2.33706	0.34393	-3.76058	0.552088			
0	-2.23223	0.358656	-3.52023	0.587777			
5	-2.0154	0.358879	-3.23106	0.593534			
10	-1.75642	0.356663	-2.80783	0.567455			
15	-1.47932	0.334499	-2.39851	0.539028			
20	-1.19459	0.291617	-2.00624	0.490577			
25	-0.94721	0.253723	-1.63138	0.432957			
30	-0.72382	0.224212	-1.29823	0.368247			
35	-0.57023	0.198877	-1.03099	0.309774			
40	-0.45229	0.182624	-0.79419	0.251157			
45	-0.30074	0.114941	-0.58561	0.199971			

No of Cells	5
No of Animals	2
**Statistical test on No of cells**	
**Wilcoxon matched-pairs signed rank test**	
P value	0.0625
Exact or approximate P value?	Exact
P value summary	ns
Significantly different? (P < 0.05)	No
One- or two-tailed P value?	Two-tailed
Sum of positive, negative ranks	0.0 , -15.00
Sum of signed ranks (W)	-15
Median of differences	
Median	-0.7485
How effective was the pairing?	
rs (Spearman)	-0.3
P value (one tailed)	0.3417
P value summary	ns
Significant correlation? (P > 0.05)	Yes

FIGURE 3D

	**FIGURE 3D**					
	C TT	C TT B2	C TT B1	C Crest	C Crest B2	C Crest B1
	p (open)	p (open)	p (open)	p (open)	p (open)	p (open)
	0.034	0.053	0.052	0.039	0.049	0.012
	0.039	0.033	0.032	0.039	0.03	0.035
	0.064	0.099	0.032	0.031	0.03	0.035
	0.064	0.056	0.048	0.031	0.041	0.088
	0.019	0.07	0.048	0.021	0.046	0.088
	0.029	0.07	0.048	0.021	0.0505	0.088
	0.088	0.095	0.05	0.071	0.05	0.088
	0.088	0.095	0.057	0.053	0.05	0.088
	0.088	0.051		0.053	0.035	0.024
	0.023	0.051		0.047	0.035	0.024
	0.044	0.15		0.016	0.103	0.051
	0.044	0.15		0.0197	0.103	0.051
	0.044	0.15		0.0197	0.143	0.051
	0.0525	0.137		0.0197	0.143	
	0.0315	0.137		0.0879	0.143	
	0.075	0.137		0.119	0.04	
	0.075	0.137		0.119	0.072	
	0.075	0.137		0.119	0.065	
	0.075	0.087		0.119	0.07	
	0.037	0.087		0.119	0.07	
	0.037	0.033		0.119		
	0.037	0.0868				
	0.022	0.0868				
	0.022	0.053				
	0.023	0.032				
	0.019	0.032				
	0.021	0.136				
	0.026	0.136				
	0.021					
	0.061					
	0.061					
	0.077					
	0.077					
	0.077					
	0.077					
	0.077					
	0.064					
	0.064					
	0.064					
	0.042					
	0.042					
	0.016					
No of channels	42	28	8	21	20	13
No of Cells	21	15	5	10	13	5
No of Animals	12	9	4	8	7	2

Statistical test on No of channels

Kruskal-Wallis test

P value	0.0018
Exact or approximate P value?	Approximate
P value summary	**
Do the medians vary signif. (P < 0.05)	Yes
Number of groups	6
Kruskal-Wallis statistic	19.14

Data summary

Number of treatments (columns)	6
Number of values (total)	132

Dunn's multiple comparisons test	Mean rank diff.	Significant?	P value
C TT vs. C TT B2	-37.46	Yes	0.0004
C TT vs. C TT B1	3.929	No	>0.9999
C Crest vs. C Crest B2	-11.54	No	>0.9999
C Crest vs. C Crest B1	-3.267	No	>0.9999
C TT vs. C Crest	-4.381	No	>0.9999
C TT B2 vs. C TT B1	41.39	Yes	0.0415
C Crest B2 vs. C Crest B1	8.273	No	>0.9999

Test details	Mean rank 1	Mean rank 2	Mean rank diff.	n1	n2
C TT vs. C TT B2	54.93	92.39	-37.46	42	28
C TT vs. C TT B1	54.93	51	3.929	42	8
C Crest vs. C Crest B2	59.31	70.85	-11.54	21	20
C Crest vs. C Crest B1	59.31	62.58	-3.267	21	13
C TT vs. C Crest	54.93	59.31	-4.381	42	21
C TT B2 vs. C TT B1	92.39	51	41.39	28	8
C Crest B2 vs. C Crest B1	70.85	62.58	8.273	20	13

FIGURE 3E

FIGURE 3E

	C TT	C TT B2	C TT B1	C Crest	C Crest B2	C Crest B1
Occurrence (%)	23.8	28.6	25	12.5	33.3	25
Total No of recordings	105	63	20	80	36	20
Recordings with channels	25	18	5	10	12	5
Recordings wihout channels	80	45	15	70	24	15

Table Analyzed	TT Co vs B2		
**Fisher's exact test**			
P value	0.5843		
P value summary	ns		
One- or two-sided	Two-sided		
Statistically significant? (alph	No		
Data analyzed	Channel	No Channel	Total
Control TT	25	80	105
B2 TT	18	45	63
Total	43	125	168

Table Analyzed	TT Co vs B1		
**Fisher's exact test**			
P value	1		
P value summary	ns		
One- or two-sided	Two-sided		
Statistically significant? (alph	No		
Data analyzed	Channel	No Channel	Total
Control TT	25	80	105
B1 TT	5	15	20
Total	30	95	125

Table Analyzed	Crest Co vs B2		
**Fisher's exact test**			
P value	0.0112		
P value summary	*		
One- or two-sided	Two-sided		
Statistically significant? (alph	Yes		
Data analyzed	Channel	No Channel	Total
Control Crest	10	70	80
B2 Crest	12	24	36
Total	22	94	116

Table Analyzed	Crest Co vs B1		
**Fisher's exact test**			
P value	0.1731		
P value summary	ns		
One- or two-sided	Two-sided		
Statistically significant? (alph	No		
Data analyzed	Channel	No Channel	Total
Control Crest	10	70	80
B1 Crest	5	15	20
Total	15	85	100

Table Analyzed	TT Co vs Crest Co		
**Fisher's exact test**			
P value	0.0594		
P value summary	ns		
One- or two-sided	Two-sided		
Statistically significant? (alph	No		
Data analyzed	Channel	No Channel	Total
Control TT	25	80	105
Control Crest	10	70	80
Total	35	150	185

FIGURE 3F

FIGURE 3F

	MI TT	MI TT B2	MI TT B1	MI Crest	MI Crest B2	MI Crest B1
	0.05	0.083	0.052	0.06	0.083	0.026
	0.067	0.049	0.022	0.06	0.083	0.029
	0.067	0.049	0.06	0.068	0.083	0.046
	0.027	0.056	0.06	0.0374	0.089	0.013
	0.075	0.056	0.04	0.138	0.101	0.04
	0.039	0.017		0.138	0.101	0.04
	0.039			0.068	0.101	0.034
	0.114			0.085	0.163	0.03
	0.011			0.085	0.163	0.023
	0.011			0.132	0.104	
				0.132	0.104	
				0.132	0.031	
				0.132	0.064	
				0.132	0.048	
				0.127	0.078	
				0.098	0.078	
				0.098		
				0.098		
No of channels	10	6	5	18	16	9
No of Cells	7	4	4	9	9	8
No of Animals	5	3	3	7	4	3

Statistical test on No of channels

Kruskal-Wallis test

P value	< 0.0001
Exact or approximate P value?	Approximate
P value summary	****
Do the medians vary signif. (P < 0.05)	Yes
Number of groups	6
Kruskal-Wallis statistic	33.76
Data summary	
Number of treatments (columns)	6
Number of values (total)	64
Number of families	1
Number of comparisons per family	7
Alpha	0.05

Dunn's multiple comparisons test	Mean rank diff.	Significant?	P value
MI TT vs. MI TT B2	-1.283	No	>0.9999
MI TT vs. MI TT B1	1.2	No	>0.9999
MI TT B2 vs. MI TT B1	2.483	No	>0.9999
MI Crest vs. MI Crest B2	3.545	No	>0.9999
MI Crest vs. MI Crest B1	35.28	Yes	<0.0001
MI Crest B2 vs. MI Crest B1	31.73	Yes	0.0003
MI TT vs. MI Crest	-24.59	Yes	0.0056

Test details	Mean rank 1	Mean rank 2	Mean rank diff.	n1	n2
MI TT vs. MI TT B2	21.8	23.08	-1.283	10	6
MI TT vs. MI TT B1	21.8	20.6	1.2	10	5
MI TT B2 vs. MI TT B1	23.08	20.6	2.483	6	5
MI Crest vs. MI Crest B2	46.39	42.84	3.545	18	16
MI Crest vs. MI Crest B1	46.39	11.11	35.28	18	9
MI Crest B2 vs. MI Crest B1	42.84	11.11	31.73	16	9
MI TT vs. MI Crest	21.8	46.39	-24.59	10	18

FIGURE 4B

FIGURE 4B

	C TT	C TT B2
	0.034	0.053
	0.039	0.033
	0.064	0.099
	0.064	0.056
	0.019	0.07
	0.029	0.07
	0.088	0.095
	0.088	0.095
	0.088	0.051
	0.023	0.051
	0.044	0.15
	0.044	0.15
	0.044	0.15
	0.0525	0.137
	0.0315	0.137
	0.075	0.137
	0.075	0.137
	0.075	0.137
	0.075	0.087
	0.037	0.087
	0.037	0.033
	0.037	0.0868
	0.022	0.0868
	0.022	0.053
	0.023	0.032
	0.019	0.032
	0.021	0.136
	0.026	0.136
	0.021	
	0.061	
	0.061	
	0.077	
	0.077	
	0.077	
	0.077	
	0.077	
	0.064	
	0.064	
	0.064	
	0.042	
	0.042	
	0.016	
No of channels	42	28
No of cells	21	15
No of animals	12	9

	C TT H89	C TT H89 B2	C TT PKAi	C TT PKAi B2	C TT KN93	C TT KN93 B2	C TT AIP	C TT AIP B2	C TT MBC	C TT MBC B2
	0.045	0.106	0.01	0.049	0.077	0.072	0.085	0.0597	0.07	0.024
	0.033	0.078	0.037	0.052	0.077	0.016	0.085	0.0556	0.07	0.022
	0.055	0.078	0.051	0.027	0.033	0.02	0.039	0.0556	0.037	0.013
	0.054	0.053	0.051	0.042	0.033	0.034	0.039	0.141	0.025	0.013
	0.054	0.044	0.024	0.042	0.081	0.031	0.039	0.036	0.025	0.079
	0.055	0.076	0.053	0.029	0.018	0.034	0.065	0.048	0.05	0.079
	0.022	0.076	0.068	0.029	0.012		0.042	0.048	0.05	0.079
	0.022		0.068		0.012		0.042	0.021		
	0.035						0.042	0.021		
	0.035						0.095			
No of channels	10	7	8	7	8	6	10	9	7	7
No of cells	7	5	6	5	5	6	5	6	4	4
No of animals	4	3	4	4	5	4	2	3	2	2

**Statistical test on No of channels**					
**Mann Whitney test**					
P value	0.0084	0.3789	0.9237	0.7038	0.6935
Exact or approximate P value?	Exact	Exact	Exact	Exact	Exact
P value summary	**	ns	ns	ns	ns
Significantly different? (P < 0.05)	Yes	No	No	No	No
One- or two-tailed P value?	Two-tailed	Two-tailed	Two-tailed	Two-tailed	Two-tailed
Sum of ranks in column C,D	64.00 , 89.00	72.00 , 48.00	61.00 , 44.00	105.0 , 85.00	56.00 , 49.00
Mann-Whitney U	9	20	23	40	21
Difference between medians					
Median of column C	0.04	0.051	0.033	0.042	0.05
Median of column D	0.076	0.042	0.0325	0.048	0.024
Difference: Actual	0.036	-0.009	-0.0005	0.006	-0.026
Difference: Hodges-Lehmann	0.031	-0.009	-0.002	-0.006	-0.003

**Statistical test on No of channels**	
**Mann Whitney test**	
P value	< 0.0001
Exact or approximate P value?	Exact
P value summary	****
Significantly different? (P < 0.05)	Yes
One- or two-tailed P value?	Two-tailed
Sum of ranks in column A,B	1156 , 1329
Mann-Whitney U	253
Difference between medians	
Median of column A	0.044
Median of column B	0.087
Difference: Actual	0.043
Difference: Hodges-Lehmann	0.043

FIGURE 4D

	FIGURE 4D						
	Control	MßCD			MßCD	MßCD + ß2-stim.	
	1	0.06957			1	7.926264	
	1	1.258874			1	19.8232	
	1	3.714582			1	2.665444	
	1	0.610328			1	1.236033	
No of animals	4				4		
	**Mann Whitney test**				**Mann Whitney test**		
	P value			> 0.9999	P value		0.0286
	Exact or approximate P value?			Exact	Exact or approximate P value?		Exact
	P value summary			ns	P value summary		*
	Significantly different? (P < 0.05)			No	Significantly different? (P < 0.05)		Yes
	One- or two-tailed P value?			Two-tailed	One- or two-tailed P value?		Two-tailed
	Sum of ranks in column A,B			18.00 , 18.00	Sum of ranks in column D,E		10.00 , 26.00
	Mann-Whitney U			8	Mann-Whitney U		0
	Difference between medians				Difference between medians		
	Median of column A			1	Median of column D		1
	Median of column B			0.9346	Median of column E		5.296
	Difference: Actual			-0.0654	Difference: Actual		4.296
	Difference: Hodges-Lehmann			-0.0654	Difference: Hodges-Lehmann		4.296

	FIGURE 4E						
	Control	MBCD			MBCD	MBCD + ß2-stim.	
	1	0.980365			1	0.933787	
	1	1.078917			1	0.840045	
	1	1.181279			1	0.759213	
	1	0.66651			1	0.803018	
	1	0.403394			1	1.834556	
No of animals	5				5		
	**Mann Whitney test**				**Mann Whitney test**		
	P value			0.6825	P value		0.127
	Exact or approximate P value?			Exact	Exact or approximate P value?		Exact
	P value summary			ns	P value summary		ns
	Significantly different? (P < 0.05)			No	Significantly different? (P < 0.05)		No
	One- or two-tailed P value?			Two-tailed	One- or two-tailed P value?		Two-tailed
	Sum of ranks in column A,B			30.00 , 25.00	Sum of ranks in column D,E		35.00 , 20.00
	Mann-Whitney U			10	Mann-Whitney U		5
	Difference between medians				Difference between medians		
	Median of column A			1	Median of column D		1
	Median of column B			0.9804	Median of column E		0.84
	Difference: Actual			-0.01964	Difference: Actual		-0.16
	Difference: Hodges-Lehmann			-0.01964	Difference: Hodges-Lehmann		-0.16

FIGURE 5B

FIGURE 5B

	C TT	C TT B2	C TT 48H	C TT B2 48H	C TT CavREM	C TT CavREM B2	C Crest	C Crest B2	C Crest 48H	C Crest B2 48H	C Crest CavREM	Co Crest B2 CavREM
	0.034	0.053	0.146	0.041	0.063	0.029	0.039	0.049	0.249	0.202	0.04	0.019
	0.039	0.033	0.0564	0.041	0.019	0.0465	0.039	0.03	0.032	0.202	0.033	0.0576
	0.064	0.099	0.07	0.033	0.09	0.05	0.031	0.03	0.022	0.202	0.033	0.0291
	0.064	0.056	0.07	0.118	0.09		0.031	0.041	0.045	0.186	0.077	
	0.019	0.07	0.096	0.118			0.021	0.046	0.018	0.186	0.011	
	0.029	0.07	0.049	0.05			0.021	0.0505	0.063	0.321		
	0.088	0.095	0.049				0.071	0.05	0.063	0.094		
	0.088	0.095	0.035				0.053	0.05		0.094		
	0.088	0.051	0.048				0.053	0.035				
	0.023	0.051	0.07				0.047	0.035				
	0.044	0.15	0.07				0.016	0.103				
	0.044	0.15					0.0197	0.103				
	0.044	0.15					0.0197	0.143				
	0.0525	0.137					0.0197	0.143				
	0.0315	0.137					0.0879	0.143				
	0.075	0.137					0.119	0.04				
	0.075	0.137					0.119	0.072				
	0.075	0.137					0.119	0.065				
	0.075	0.087					0.119	0.07				
	0.037	0.087					0.119	0.07				
	0.037	0.033					0.119					
	0.037	0.0868										
	0.022	0.0868										
	0.022	0.053										
	0.023	0.032										
	0.019	0.032										
	0.021	0.136										
	0.026	0.136										
	0.021											
	0.061											
	0.061											
	0.077											
	0.077											
	0.077											
	0.077											
	0.077											
	0.064											
	0.064											
	0.064											
	0.042											
	0.042											
	0.016											
No of channels	42	28	11	6	4	3	21	20	7	8	5	3
No of Cells	21	15	8	4	3	3	10	13	6	4	4	3
No of Animals	12	9	4	3	3	3	8	7	3	3	4	1

Statistical test on No of channels

Kruskal-Wallis test

P value	< 0.0001				
Exact or approximate P value?	Approximate				
P value summary	****				
Do the medians vary signif. (P < 0.05)	Yes				
Number of groups	12				
Kruskal-Wallis statistic	40.92				
Data summary					
Number of treatments (columns)	12				
Number of values (total)	158				
Number of families	1				
Number of comparisons per family	6				
Alpha	0.05				
Dunn's multiple comparisons test	Mean rank diff.	Significant?	P value		
C TT vs. C TT B2	-42.39	Yes	0.0009		
C TT 48H vs. C TT B2 48H	10.14	No	>0.9999		
C Crest vs. C Crest B2	-13.18	No	>0.9999		
C Crest 48H vs. C Crest B2 48H	-84.48	Yes	0.0022		
C TT CavREM vs. C TT CavREM B2	32.88	No	>0.9999		
C Crest CavREM vs. Co Crest B2 CavREM	8.767	No	>0.9999		
Test details	Mean rank 1	Mean rank 2	Mean rank diff.	n1	n2
C TT vs. C TT B2	63.39	105.8	-42.39	42	28
C TT 48H vs. C TT B2 48H	87.73	77.58	10.14	11	6
C Crest vs. C Crest B2	68.64	81.83	-13.18	21	20
C Crest 48H vs. C Crest B2 48H	62.14	146.6	-84.48	7	8
C TT CavREM vs. C TT CavREM B2	82.88	50	32.88	4	3
C Crest CavREM vs. Co Crest B2 CavREM	45.6	36.83	8.767	5	3

FIGURE 5C

	FIGURE 5C											
	C TT	C TT B2	C TT 48H	C TT B2 48H	C TT REM	C TT REM B2	C Crest	C Crest B2	C Crest 48H	C Crest B2 48H	C Crest REM	C Crest REM B2
	23.8	28.6	25.8	27.8	18.8	12	12.5	33.3	27.3	16.6	16.6	20
Total No of recordings	105	63	31	18	16	25	80	36	22	30	24	20
Recordings with channels	25	18	8	5	3	3	10	12	6	5	4	4
Recordings wihout channels	80	45	23	13	13	22	70	24	16	25	20	16

Table Analyzed	TT vs TT B2		
**Fisher's exact test**			
P value	0.5843		
P value summary	ns		
One- or two-sided	Two-sided		
Statistically significant? (alpha<0.05)	No		
Data analyzed	Channel	No Channel	Total
Row 1	25	80	105
Row 2	18	45	63
Total	43	125	168

Table Analyzed	TT 48H vs TT 48H B2		
**Fisher's exact test**			
P value	1		
P value summary	ns		
One- or two-sided	Two-sided		
Statistically significant? (alpha<0.05)	No		
Data analyzed	Channel	No Channel	Total
Row 1	8	23	31
Row 2	5	13	18
Total	13	36	49

Table Analyzed	TT REM vs TT REM B2		
**Fisher's exact test**			
P value	0.6624		
P value summary	ns		
One- or two-sided	Two-sided		
Statistically significant? (alpha<0.05)	No		
Data analyzed	Channel	No Channel	Total
Row 1	3	13	16
Row 2	3	22	25
Total	6	35	41

Table Analyzed	Crest vs Crest B2		
**Fisher's exact test**			
P value	0.0112		
P value summary	*		
One- or two-sided	Two-sided		
Statistically significant? (alpha<0.05)	Yes		
Data analyzed	Channel	No Channel	Total
Row 1	10	70	80
Row 2	12	24	36
Total	22	94	116

Table Analyzed	Crest 48H vs Crest 48H B2		
**Fisher's exact test**			
P value	0.4946		
P value summary	ns		
One- or two-sided	Two-sided		
Statistically significant? (alpha<0.05)	No		
Data analyzed	Channel	No Channel	Total
Row 1	6	16	22
Row 2	5	25	30
Total	11	41	52

Table Analyzed	Crest REM vs Crest REM B2		
**Fisher's exact test**			
P value	1		
P value summary	ns		
One- or two-sided	Two-sided		
Statistically significant? (alpha<0.05)	No		
Data analyzed	Channel	No Channel	Total
Row 1	4	20	24
Row 2	4	16	20
Total	8	36	44

FIGURE 6A

FIGURE 6A			
	Control	Cav3KO	
	21.22346	18.67983	
	97.00047	0	
	187.0059	20.36758	
	104.6768	28.00481	
	96.41114	19.60524	
	93.68228	20.75621	
No of animals	6	6	
**Mann Whitney test**			
P value			0.0043
Exact or approximate P value?			Exact
P value summary			**
Significantly different? (P < 0.05)			Yes
One- or two-tailed P value?			Two-tailed
Sum of ranks in column A,B			56.00 , 22.00
Mann-Whitney U			1
Difference between medians			
Median of column A			96.71
Median of column B			19.99
Difference: Actual			-76.72
Difference: Hodges-Lehmann			-76.74

FIGURE 6B

FIGURE 6B

Control	Cav3KO
59.70891	93.34528
127.0936	94.26332
63.39901	80.63144
91.14644	88.56695
147.0757	169.8791
53.72145	115.5889
87.90416	53.34975
63.93193	124.1603
57.96238	67.94447
78.54904	22.53471
144.9261	40.78818
131.9033	111.76
92.02418	80.927
120.4613	64.71115
93.07658	46.42633
83.46619	12.7631
118.661	61.5047
186.2785	48.75504
151.9077	122.3601
78.11017	41.10614
81.88088	53.51545
139.7358	41.84505
100.8867	85.55307
145.0515	72.65562
75.49485	143.9946
117.9579	37.68921
107.8639	65.60681
99.0506	50.55531
115.6516	59.68652
153.6588	71.58979
41.10166	105.4232
158.7192	44.42454
173.0139	86.87864
125.2844	27.88177
145.0828	86.39498
160.2821	85.48142
28.61173	104.0752
50.18809	99.485
84.51411	33.70802
170.9539	97.98477
172.8303	71.08374
74.84998	90.31796
86.64577	141.8406
100.8957	96.63233
100.7613	114.0931
124.0081	79.34169
89.90148	84.54098
58.17734	70.54635
122.3197	66.85177
33.74384	92.77206
120.5867	30.58218
113.6319	34.85446
146.6413	
90.29109	
109.1178	
56.80251	
180.5105	
99.5253	
122.5929	
73.39006	
92.74071	
71.33005	
41.20914	
37.4026	
25.49933	
154.223	
37.23242	
61.64801	
97.72951	
39.15808	
53.25571	
175.1232	
124.1066	
125.5038	
96.29198	
87.8773	
107.7429	
192.7004	
42.57949	
56.16659	
56.2069	
178.6207	
185.2172	
54.65741	
92.70936	
70.94044	
85.74563	
102.8571	
75.1142	
157.6668	
87.6086	
127.9758	
56.82042	
71.41066	
104.1021	
60.22839	
70.5777	
86.93238	
71.84953	

	Control	Cav3KO	
No of cells	99	52	
No of Animals	6	6	
**Statistical test on No of cells**			
**Mann Whitney test**			
P value			0.0009
Exact or approximate P value?			Exact
P value summary			***
Significantly different? (P < 0.05)			Yes
One- or two-tailed P value?			Two-tailed
Sum of ranks in column A,B			8362 , 3114
Mann-Whitney U			1736
Difference between medians			
Median of column A			92.74
Median of column B			76
Difference: Actual			-16.74
Difference: Hodges-Lehmann			-21.93

FIGURE 6D

	FIGURE 6D								
	mV	Control	SEM		Cav3KO	SEM	Inset Control Vs Cav3KO at 10 mV		
	-50	-0.19521	0.046535		-0.14741	0.017118			
	-45	-0.15013	0.023528		-0.09922	0.01139		Control	Cav3KO
	-40	-0.09252	0.011262		-0.1145	0.01474		-2.22803	-4.079627
	-35	-0.13674	0.014387		-0.15885	0.011496		-3.210899	-2.239852
	-30	-0.20805	0.026698		-0.26583	0.03143		-3.29942	-3.41062
	-25	-0.35449	0.056083		-0.49511	0.084932		-1.504711	-3.281129
	-20	-0.62642	0.101007		-0.73602	0.131337		-3.799643	-3.404857
	-15	-1.14339	0.186169		-1.08145	0.151962		-4.54122	-2.192691
	-10	-2.04935	0.371681		-1.52334	0.154734		-4.78519	-4.767418
	-5	-3.04854	0.537093		-2.1966	0.134944		-6.26543	-4.550494
	0	-3.76702	0.608452		-2.95472	0.172526		-3.888477	-4.042557
	5	-4.15317	0.617661		-3.48167	0.217373		-10.79278	-2.363806
	10	-4.22227	0.60253		-3.70237	0.241904		-5.109229	-4.492747
	15	-3.99683	0.577694		-3.64903	0.249643		-2.435249	-5.840243
	20	-3.62122	0.533358		-3.37485	0.245128		-3.838329	-3.989843
	25	-3.12251	0.476692		-2.92637	0.22418		-3.413131	-3.496436
	30	-2.58345	0.412938		-2.42263	0.196071			-3.556945
	35	-2.06902	0.346596		-1.91777	0.164946			-3.528642
	40	-1.58626	0.284537		-1.42822	0.131226			
	45	-1.25491	0.234627		-1.03498	0.100498			
No of cells		14			16			14	16
No of animals		4			4			4	4

**Statistical test on No of cells**	
**Mann Whitney test**	
P value	0.779
Exact or approximate P value?	Exact
P value summary	ns
Significantly different? (P < 0.05)	No
One- or two-tailed P value?	Two-tailed
Sum of ranks in column A,B	210.0 , 255.0
Mann-Whitney U	105
Difference between medians	
Median of column A	-3.819
Median of column B	-3.543
Difference: Actual	-0.2762
Difference: Hodges-Lehmann	-0.1017

FIGURE 6G

	FIGURE 6G								
	TT Co	TT B2	Crest Co	Crest B2		TT KO	TT KO B2	Crest KO	Crest KO B2
	0.0276	0.1275	0.0258	0.18		0.1102	0.0885	0.1486	0.0258
	0.0276	0.1275	0.0258	0.18		0.0302	0.0885	0.1486	0.0878
	0.054	0.1275	0.0608	0.18		0.0737	0.0885	0.014	0.0193
	0.0855	0.1275	0.0608	0.18		0.1199	0.0329	0.014	0.0326
	0.0191	0.044	0.0451	0.18		0.0769	0.0403	0.0417	0.109
	0.0191	0.044	0.0957	0.1163		0.0769	0.0362	0.0821	0.109
	0.0797	0.0348	0.0957	0.1163		0.0769	0.042	0.0844	0.109
	0.0797	0.1245	0.0957	0.0622		0.0466		0.0844	0.0656
	0.0498	0.1245	0.0957	0.0622				0.0844	0.0656
	0.0498	0.1245	0.0274	0.0356				0.0441	0.0656
	0.0498	0.0845	0.0163	0.019				0.0441	
		0.0845	0.0163					0.0289	
		0.0543						0.0289	
								0.0581	
								0.0437	
No of channels	11	13	12	11		8	7	15	10
No of cells	6	6	6	5		6	5	9	6
No of animals	4	3	2	3		3	3	4	3
	**Statistical test on No of channels**								
	**Kruskal-Wallis test**								
	P value				0.0223				
	Exact or approximate P value?				Approximate				
	P value summary				*				
	Do the medians vary signif. (P < 0.05)				Yes				
	Number of groups				8				
	Kruskal-Wallis statistic				16.32				
	Data summary								
	Number of treatments (columns)				8				
	Number of values (total)				87				
	Number of families				1				
	Number of comparisons per family				4				
	Alpha				0.05				
	Dunn's multiple comparisons test				Mean rank diff.	Significant?	P value		
	TT Co vs. TT B2				-28.13	Yes	0.0261		
	Crest Co vs. Crest B2				-27.63	Yes	0.035		
	TT KO vs. TT KO B2				8.893	No	>0.9999		
	Crest KO vs. Crest KO B2				-5.9	No	>0.9999		
	Test details			Mean rank 1	Mean rank 2	Mean rank diff.	n1	n2	
	TT Co vs. TT B2			30.64	58.77	-28.13	11	13	
	Crest Co vs. Crest B2			33.92	61.55	-27.63	12	11	
	TT KO vs. TT KO B2			47.75	38.86	8.893	8	7	
	Crest KO vs. Crest KO B2			37.2	43.1	-5.9	15	10	

FIGURE 7B

	**FIGURE 7B**							
	Co TT	B2 TT	Co Crest	B2 Crest				
	0.0375	0.158	0.085	0.403				
	0.0375	0.158	0.085	0.155				
	0.06	0.158	0.085	0.155				
	0.06	0.257	0.0597	0.155				
	0.06	0.386	0.0597	0.155				
	0.06		0.0597	0.155				
	0.06		0.0597	0.155				
	0.114							
	0.066							
No of channels	9	5	7	7				
No of cells	4	3	2	2				
No of patients	3	1	1	1				
	**Statistical test on No of channels**							
	**Kruskal-Wallis test**							
	P value			0.0001				
	Exact or approximate P value?			Approximate				
	P value summary			***				
	Do the medians vary signif. (P < 0.			Yes				
	Number of groups			4				
	Kruskal-Wallis statistic			21.08				
	Data summary							
	Number of treatments (columns)			4				
	Number of values (total)			28				
	Number of families			1				
	Number of comparisons per family			3				
	Alpha			0.05				
	Dunn's multiple comparisons test			Mean rank diff.	Significant?	P value		
	Co TT vs. B2 TT			-16.56	Yes	0.0008		
	Co Crest vs. B2 Crest			-12.14	Yes	0.0158		
	Co TT vs. Co Crest			-0.127	No	>0.9999		
	Test details			Mean rank 1	Mean rank 2	Mean rank diff.	n1	n2
	Co TT vs. B2 TT			8.444	25	-16.56	9	5
	Co Crest vs. B2 Crest			8.571	20.71	-12.14	7	7
	Co TT vs. Co Crest			8.444	8.571	-0.127	9	7

FIGURE 7C

	FIGURE 7C			
	Co TT	B2 TT	Co Crest	B2 Crest
	18.18	33.3	10	22.2
Total No of recordings	22	9	20	9
Recordings with channels	4	3	2	2
Recordings wihout channels	18	6	18	7

Table Analyzed	TT vs TT B2			
**Fisher's exact test**				
P value		0.3841		
P value summary		ns		
One- or two-sided		Two-sided		
Statistically significant? (alpha<0.05)		No		
Data analyzed		Channel	No Channel	Total
Row 1		4	18	22
Row 2		3	6	9
Total		7	24	31

Table Analyzed	Crest vs Crest B2			
**Fisher's exact test**				
P value		0.568		
P value summary		ns		
One- or two-sided		Two-sided		
Statistically significant? (alpha<0.05)		No		
Data analyzed		Channel	No Channel	Total
Row 1		2	18	20
Row 2		2	7	9
Total		4	25	29

Table Analyzed	TT vs Crest			
**Fisher's exact test**				
P value		0.6653		
P value summary		ns		
One- or two-sided		Two-sided		
Statistically significant? (alpha<0.05)		No		
Data analyzed		Channel	No Channel	Total
Row 1		4	18	22
Row 2		2	18	20
Total		6	36	42

FIGURE 7D

FIGURE 7D			
DCM TT	DCM TT B2	DCM Crest	DCM Crest B2
0.023	0.023	0.191	0.034
0.019	0.023	0.095	0.034
0.019	0.084	0.057	0.063
0.044	0.084	0.057	0.044
0.028	0.053	0.254	0.053
0.012	0.053	0.108	0.173
0.012	0.035	0.127	
0.087	0.0756	0.127	
0.087	0.0756	0.114	
0.101	0.0756	0.114	
0.101		0.114	
0.037		0.038	
0.0055		0.068	
0.0055			
0.025			
0.057			
0.016			
0.016			
0.048			
0.048			

	DCM TT	DCM TT B2	DCM Crest	DCM Crest B2
No of channels	20	10	13	6
No of cells	13	5	9	5
No of patients	7	2	7	2

Statistical test on No of channels

Kruskal-Wallis test

P value		0.0004			
Exact or approximate P value?		Approximate			
P value summary		***			
Do the medians vary signif. (P < 0.05)		Yes			
Number of groups		4			
Kruskal-Wallis statistic		18.43			
Data summary					
Number of treatments (columns)		4			
Number of values (total)		49			
Number of families	1				
Number of comparisons per family	3				
Alpha	0.05				
Dunn's multiple comparisons test	Mean rank diff.	Significant?	P value		
DCM TT Co vs. DCM TT B2	-8.425	No	0.3828		
DCM Crest Co vs. DCM Crest B2	13.56	No	0.1631		
DCM Crest Co vs. DCM TT Co	21.83	Yes	<0.0001		
Test details	Mean rank 1	Mean rank 2	Mean rank diff.	n1	n2
DCM TT Co vs. DCM TT B2	16.48	24.9	-8.425	20	10
DCM Crest Co vs. DCM Crest B2	38.31	24.75	13.56	13	6
DCM Crest Co vs. DCM TT Co	38.31	16.48	21.83	13	20

FIGURE 7E

	**FIGURE 7E**			
	DCM TT	DCM TT B2	DCM Crest	DCM Crest B2
	21	26.3	27.5	21.21
Total No of recordings	62	19	40	33
Recordings with channels	13	5	11	7
Recordings wihout channels	49	14	29	26

Table Analyzed	TT vs TT B2			
Fisher's exact test				
P value		0.7532		
P value summary		ns		
One- or two-sided		Two-sided		
Statistically significant? (alpha<0.05)		No		
Data analyzed		Channel	No Channel	Total
Row 1		13	49	62
Row 2		5	14	19
Total		18	63	81

Table Analyzed	Crest vs Crest B2			
Fisher's exact test				
P value		0.5942		
P value summary		ns		
One- or two-sided		Two-sided		
Statistically significant? (alpha<0.05)		No		
Data analyzed		Channel	No Channel	Total
Row 1		11	29	40
Row 2		7	26	33
Total		18	55	73

Table Analyzed	TT vs Crest			
Fisher's exact test				
P value		0.4803		
P value summary		ns		
One- or two-sided		Two-sided		
Statistically significant? (alpha<0.05)		No		
Data analyzed		Channel	No Channel	Total
Row 1		13	49	62
Row 2		11	29	40
Total		24	78	102

FIGURE 7G

	**FIGURE 7G**							
	C TT	C TT B2	C Crest	C Crest B2				
	21.3	22.65	12.4	19.7				
	19.3	22.65	12.4	23.5				
	13.52	22.65	12.4	23.5				
	11.6	22.1	14.8	23.5				
	13.52	21.4	14.8	23.5				
	11.6		14.8	23.5				
	11.6		14.8	23.5				
	11.6							
	11.6							
No of channels	9	5	7	7				
No of cells	6	3	2	2				
No of patients	4	1	1	1				
	**Statistical test on No of channels**							
	**Kruskal-Wallis test**							
	P value				0.0001			
	Exact or approximate P value?				Approximate			
	P value summary				***			
	Do the medians vary signif. (P < 0.05)				Yes			
	Number of groups				4			
	Kruskal-Wallis statistic				21.08			
	Data summary							
	Number of treatments (columns)				4			
	Number of values (total)				28			
	Number of families				1			
	Number of comparisons per family				2			
	Alpha				0.05			
	Dunn's multiple comparisons test			Mean rank diff.	Significant?	P value		
	C TT vs. C TT B2			-12.67	Yes	0.0106		
	C Crest vs. C Crest B2			-14	Yes	0.0026		
	Test details			Mean rank 1	Mean rank 2	Mean rank diff.	n1	n2
	C TT vs. C TT B2			7.333	20	-12.67	9	5
	C Crest vs. C Crest B2			10.14	24.14	-14	7	7

FIGURE 7I

	FIGURE 7I						
	C TT	C TT B2	C Crest	C Crest B2			
	12.9	9.2	8.27	11.3			
	12.9	5.45	8.27	11.3			
	5.2	5.45	16	14.2			
	5.2	6.05	13.6	15.8			
	15.3	6.05	11.3	15.8			
	15.3	7.18	20.1	19.95			
	11.9	7.18	20.1	4.85			
	11.9		20.1	4.85			
No of channels	8	7	8	6			
No of cells	4	4	5	5			
No of patients	4	1	3	2			

Statistical test on No of channels

Kruskal-Wallis test

P value	0.025
Exact or approximate P value?	Approximate
P value summary	*
Do the medians vary signif. (P < 0.05)	Yes
Number of groups	4
Kruskal-Wallis statistic	9.35
Data summary	
Number of treatments (columns)	4
Number of values (total)	29
Number of families	1
Number of comparisons per family	3
Alpha	0.05

Dunn's multiple comparisons test	Mean rank diff.	Significant?	P value
C TT vs. C TT B2	7.714	No	0.3015
C Crest vs. C Crest B2	3.833	No	0.7419
C TT vs. C Crest	-5.5	No	0.5583

Test details	Mean rank 1	Mean rank 2	Mean rank diff.	n1	n2
C TT vs. C TT B2	15	7.286	7.714	8	7
C Crest vs. C Crest B2	20.5	16.67	3.833	8	6
C TT vs. C Crest	15	20.5	-5.5	8	8

SUPP. FIGURE 1A

	**Supp.Fig.1A**			
	Co TT	Co Crest	Failing TT	Failing Crest
	0	84.61539	46	14.97326
	21.875	84.61539	25.37313	1.449275
	21.875	103.2258	25.37313	1.449275
	35.13514	103.2258	-51.2821	57.35294
	35.13514	90.14085	-51.2821	-1.176471
	35.13514	15.12605	-22.807	-1.176471
	6.666667	15.12605	45.45455	44.69697
	6.666667	15.12605	45.45455	44.69697
	6.666667	15.12605		44.69697
	6.666667	15.12605		44.69697
	71.42857	15.12605		44.69697
	-32.7869	41.50943		-24.40945
	-32.7869	41.50943		13.26531
	74.02597	80.71066		13.26531
	74.02597	80.71066		13.26531
	74.02597	80.71066		
	74.02597	4.664392		
	74.02597			
	73.4375			
	73.4375			
	73.4375			
No of channels	21	17	8	15
No of cells	8	7	5	7
No of animals	6	5	4	7
**Statistical test on No of channels**				
**Wilcoxon Signed Rank Test**				
Theoretical median	0	0	0	0
Actual median	35.14	41.51	25.37	13.27
Discrepancy	-35.14	-41.51	-25.37	-13.27
Sum of signed ranks (W)	180	153	4	96
Sum of positive ranks	195	153	20	108
Sum of negative ranks	-15	0	-16	-12
P value (two tailed)	0.0002	< 0.0001	0.8281	0.0039
Exact or estimate?	Exact	Exact	Exact	Exact
Significant (alpha=0.05)?	Yes	Yes	No	Yes
Sum	772.1	886.4	62.28	311.7

SUPP. FIGURE 1B

	**Supp.Fig.1B**							
	Control TT	ISO TT	Control Crest	ISO CREST	Failing TT	Failing TT ISO	Failing CREST	Failing CREST
	0.767	0.769	0.71	0.722	0.655	0.657	0.691	0.687
	0.745	0.853	0.71	0.722	0.631	0.676	0.727	0.767
	0.745	0.853	0.703	0.718	0.631	0.676	0.727	0.767
	0.674	0.692	0.703	0.718	0.693	0.714	0.5593	0.597
	0.674	0.692	0.751	0.739	0.693	0.714	0.726	0.732
	0.674	0.692	0.727	0.769	0.549	0.572	0.726	0.732
	0.69	0.7	0.727	0.769	0.518	0.542	0.862	0.875
	0.69	0.7	0.727	0.769	0.518	0.542	0.862	0.875
	0.69	0.7	0.727	0.769			0.862	0.875
	0.69	0.7	0.727	0.769			0.862	0.875
	0.598	0.625	0.727	0.769			0.862	0.875
	0.6	0.595	0.652	0.655			0.693	0.714
	0.6	0.595	0.652	0.655			0.66	0.671
	0.677	0.697	0.609	0.56			0.66	0.671
	0.677	0.697	0.609	0.56			0.66	0.671
	0.677	0.697	0.609	0.56				
	0.677	0.697	0.876	0.85				
	0.677	0.697						
	0.535	0.549						
	0.535	0.549						
	0.535	0.549						
No of Channels	21		17		8		15	
No of Cells	8		7		5		7	
No of Animals	6		5		4		7	
**Statistical test on No of channels**								
**Wilcoxon matched-pairs signed rank test**								
P value	< 0.0001		0.4169		0.0078		0.0001	
Exact or approximate P value?	Exact		Exact		Exact		Exact	
P value summary	****		ns		**		***	
Significantly different? (P < 0.05)	Yes		No		Yes		Yes	
One- or two-tailed P value?	Two-tailed		Two-tailed		Two-tailed		Two-tailed	
Sum of positive, negative ranks	226.0 , -5.000		94.00 , -59.00		36.00 , 0.0		119.0 , -1.000	
Sum of signed ranks (W)	221		35		36		118	
Median of differences								
Median	0.018		0.012		0.0235		0.013	
How effective was the pairing?								
rs (Spearman)	0.992		0.9457		0.9259		1	
P value (one tailed)	< 0.0001		< 0.0001		0.0018		< 0.0001	
P value summary	****		****		**		****	
Significant correlation? (P > 0.05)	No		No		No		No	

SUPP. FIGURE 1C

	Supp.Fig.1C			
	Co TT	Co Crest	Failing TT	Failing Crest
	0.260756	1.690141	0.305344	-0.5788712
	14.49664	1.690141	7.131537	5.502063
	14.49664	2.133713	7.131537	5.502063
	2.670623	2.133713	3.030303	6.740569
	2.670623	-1.59787	3.030303	0.8264463
	2.670623	5.777166	4.189435	0.8264463
	1.449275	5.777166	4.633205	1.508121
	1.449275	5.777166	4.633205	1.508121
	1.449275	5.777166		1.508121
	1.449275	5.777166		1.508121
	4.51505	5.777166		1.508121
	-0.83333	0.460123		3.030303
	-0.83333	0.460123		1.666667
	2.95421	-8.04598		1.666667
	2.95421	-8.04598		1.666667
	2.95421	-8.04598		
	2.95421	-2.96804		
	2.95421			
	2.616822			
	2.616822			
	2.616822			
No of channels	21	17	8	15
No of cells	8	7	5	7
No of animals	6	5	4	7
**Statistical test on No of channels**				
**Wilcoxon Signed Rank Test**				
Theoretical median	0	0	0	0
Actual median	2.671	1.69	4.411	1.508
Discrepancy	-2.671	-1.69	-4.411	-1.508
Sum of signed ranks (W)	221	35	36	118
Sum of positive ranks	226	94	36	119
Sum of negative ranks	-5	-59	0	-1
P value (two tailed)	< 0.0001	0.4169	0.0078	0.0001
Exact or estimate?	Exact	Exact	Exact	Exact
Significant (alpha=0.05)?	Yes	No	Yes	Yes
Sum	68.53	14.53	34.08	34.39

SUPP. FIGURE 1E & 1F

	Supp.Fig.1E	
	Control	Failing
	14.6143	13.0912
	10.9765	11.0897
	11.4969	8.25575
	15.5159	11.7659
	8.19112	10.9329
	5.91284	12.0225
	12.3577	9.68326
	7.3018	11.4956
	15.623	13.9247
	10.3974	10.1026
	5.98964	9.93845
	13.6775	7.76818
	8.37157	8.5842
	12.0726	19.0983
	6.67844	17.5899
	8.60404	14.3083
	9.81911	12.3063
	10.7153	12.6922
	10.0631	11.9306
	9.13974	12.9926
	8.38917	
	7.39163	
	7.82272	
	8.24006	
	6.76495	
	8.49991	
	10.277	
	8.30394	
	11.506	
	13.0639	
	14.0426	
	9.3006	
	7.40296	
	8.76447	
	6.881	
	11.1927	
	8.83747	
	7.68826	
No of cells	38	20
No of animals	11	4
**Statistical test on No of cells**		
**Mann Whitney test**		
P value	0.0057	
Exact or approximate P value?	Exact	
P value summary	**	
Significantly different? (P < 0.05)	Yes	
One- or two-tailed P value?	Two-tailed	
Sum of ranks in column A,B	954.0 , 757.0	
Mann-Whitney U	213	
Difference between medians		
Median of column A	8.989	
Median of column B	11.85	
Difference: Actual	2.86	
Difference: Hodges-Lehmann	2.272	

	Supp.Fig.1F	
	Control	Failing
	113.662	200.174
	124.052	127.772
	71.9327	101.832
	103.455	196.736
	149.124	119.41
	10.3715	194.653
	163.532	163.441
	137.48	107.557
	76.8568	96.1646
	52.3188	134.116
	97.9842	125.643
	14.1425	149.395
	154.324	67.9351
	156.26	20.4568
	42.0802	126.489
	151.911	278.123
	36.1556	64.4949
	53.3197	295.72
	16.8984	175.103
	11.7567	181.802
	41.1459	
	120.577	
	128.688	
	189.443	
	76.1478	
	99.0092	
	54.8882	
	34.7166	
	140.467	
	206.44	
	91.6246	
	181.008	
	297.858	
	186.292	
	128.997	
	269.872	
	152.477	
	67.9429	
No of cells	38	20
No of animals	11	4
**Statistical test on No of cells**		
**Mann Whitney test**		
P value	0.0703	
Exact or approximate P value?	Exact	
P value summary	ns	
Significantly different? (P < 0.05)	No	
One- or two-tailed P value?	Two-tailed	
Sum of ranks in column A,B	1010 , 701.0	
Mann-Whitney U	269	
Difference between medians		
Median of column A	108.6	
Median of column B	130.9	
Difference: Actual	22.39	
Difference: Hodges-Lehmann	35.37	

SUPP. FIGURE 2B, 2D & 2F

Supp. Fig. 2B

	Control	ISO	Failing	ISO
	1	3.102692	1	0.605381
	1	4.017831	1	0.754435
	1	1.182417	1	2.446207
	1	1.418454	1	2.615628
	1	1.555491		
	1	1.019919		
	1	0.946152		
	1	1.197877		
	1	0.833894		
No of animals	9		4	
**Mann Whitney test**				
P value	0.0361		> 0.9999	
Exact or approximate P value?	Exact		Exact	
P value summary	*		ns	
Significantly different? (P < 0.05)	Yes		No	
One- or two-tailed P value?	Two-tailed		Two-tailed	
Sum of ranks in column A,B	63.00 , 108.0		18.00 , 18.00	
Mann-Whitney U	18		8	
Difference between medians				
Median of column A	1		1	
Median of column B	1.198		1.6	
Difference: Actual	0.1979		0.6003	
Difference: Hodges-Lehmann	0.1979		0.6003	

Supp. Fig. 2D

	Control	ß1 stim.	ß2 stim.		
	1	0.832873	0.658815		
	1	1.156501	1.300859		
	1	0.499278	0.681421		
	1	1.475365	1.376142		
	1	1.013772	1.193066		
	1	0.919648	1.557508		
No of animals	6	6	6		
**Kruskal-Wallis test**					
P value	0.5976				
Exact or approximate P value?	Exact				
P value summary	ns				
Do the medians vary signif. (P < 0.05)	No				
Number of groups	3				
Kruskal-Wallis statistic	1.104				
Data summary					
Number of treatments (columns)	3				
Number of values (total)	18				
Number of families	1				
Number of comparisons per family	2				
Alpha	0.05				
Dunn's multiple comparisons test	Mean rank diff.	Significant?	P value		
Control vs. ß1 stim.	-0.1667	No	>0.9999		
Control vs. ß2 stim.	-2.833	No	0.6982		
Test details	Mean rank 1	Mean rank 2	Mean rank diff.	n1	n2
Control vs. ß1 stim.	8.5	8.667	-0.1667	6	6
Control vs. ß2 stim.	8.5	11.33	-2.833	6	6

Supp.Fig.2F

	Control	MBCD	MBCD + ß2-stim.	MBCD	MBCD + ß2-stim.
	1	0.707152	0.612563	1	0.86624
	1	3.174544	1.22421	1	0.385633
	1	0.839402	1.149215	1	1.369088
	1	4.206316	3.044475	1	0.723787
	1	1.369775	0.889367	1	0.889367
No of animals	5	5	5	5	5
**Wilcoxon matched-pairs signed rank test**					
P value	0.3125			0.4375	
Exact or approximate P value?	Exact			Exact	
P value summary	ns			ns	
Significantly different? (P < 0.05)	No			No	
One- or two-tailed P value?	Two-tailed			Two-tailed	
Sum of positive, negative ranks	12.00 , -3.000			4.000 , -11.00	
Sum of signed ranks (W)	9			-7	
Median of differences					
Median	0.3698			-0.1338	

SUPP. FIGURE 3E

	Supp. Figure 3E					
	C TT	C TT B2	C TT B1	C Crest	C Crest B2	C Crest B1
	7	11.02	4.2	6.05	9.9	7.6
	6.86	11.7	5.2	6.05	6.4	4.3
	6.86	11.09	5.9	4.8	6.4	4.3
	5.37	11.45	5.9	4.8	6.7	
	5.37	6.3	2.9	6.8	10.3	
	5.84	6.3	2.9	6.8	9.3	
	6.8	6.95	2.9		5.9	
	10.6	6.95	9.9		5.9	
	1.9	8.7			10.2	
	1.9				10.2	
	1.9				6.3	
	5.47					
	5.47					
	7.94					
No of channels	14	9	8	6	11	3
No of cells	9	7	5	3	9	2
No of animals	7	6	3	3	5	2

Statistical test on No of channels

Kruskal-Wallis test

P value	0.0029
Exact or approximate P value?	Approximate
P value summary	**
Do the medians vary signif. (P < 0.05)	Yes
Number of groups	6
Kruskal-Wallis statistic	18.02
Data summary	
Number of treatments (columns)	6
Number of values (total)	51
Number of families	1
Number of comparisons per family	6
Alpha	0.05

Dunn's multiple comparisons test	Mean rank diff.	Significant?	P value
C TT vs. C TT B2	-18.32	Yes	0.0234
C TT vs. C TT B1	7.134	No	>0.9999
C TT B2 vs. C TT B1	25.45	Yes	0.0025
C Crest vs. C Crest B2	-11.98	No	0.6713
C Crest vs. C Crest B1	3	No	>0.9999
C Crest B2 vs. C Crest B1	14.98	No	0.7285

Test details	Mean rank 1	Mean rank 2	Mean rank	n1	n2
C TT vs. C TT B2	21.57	39.89	-18.32	14	9
C TT vs. C TT B1	21.57	14.44	7.134	14	8
C TT B2 vs. C TT B1	39.89	14.44	25.45	9	8
C Crest vs. C Crest B2	21.33	33.32	-11.98	6	11
C Crest vs. C Crest B1	21.33	18.33	3	6	3
C Crest B2 vs. C Crest B1	33.32	18.33	14.98	11	3

SUPP. FIGURE 3F

	Supp. Figure 3F					
	F TT	F TT B2	F TT B1	F Crest	F Crest B2	F Crest B1
	4.2	4.75	4.5	11.3	4.67	5.9
	4.89	7.7	2.2	8.6	4.67	4.8
	4.89	4.89	4.2	8.6	9.9	7.6
	2.94	4.89	4.2	5.4	10.1	4.7
	5.8	3.3	8.7	6.46	10.1	4.7
	4.3	3.3		6.46	10.1	10
		3.2		4.8	4.5	8.4
		3.2		4.8	4.5	4.2
					8.9	9.4
					8.9	
					10.2	
					6.7	
					6.7	
					9.1	
					9.1	
No of channels	6	8	5	9	18	9
No of cells	5	5	4	6	8	8
No of animals	3	4	2	3	5	3

Statistical test on No of channels

Kruskal-Wallis test

P value		0.0045
Exact or approximate P value?		Approximate
P value summary		**
Do the medians vary signif. (P < 0.05)		Yes
Number of groups		6
Kruskal-Wallis statistic		16.99
Data summary		
Number of treatments (columns)		6
Number of values (total)		53
Number of	1	
Number of	9	
Alpha	0.05	

Dunn's multiple comparisons test	Mean rank diff.	Significant?	P value
F TT vs. F TT B2	1.292	No	>0.9999
F TT vs. F TT B1	2.717	No	>0.9999
F TT B2 vs. F TT B1	1.425	No	>0.9999
F Crest vs. F Crest B2	-0.4444	No	>0.9999
F Crest vs. F Crest B1	5.389	No	>0.9999
F Crest B2 vs. F Crest B1	5.833	No	>0.9999
F TT vs. F Crest	-17.64	No	0.4684
F TT B2 vs. F Crest B2	-19.38	Yes	0.0188
F TT B1 vs. F Crest B1	-14.97	No	0.7853

Test details	Mean rank 1	Mean rank 2	Mean rank	n1	n2
F TT vs. F TT B2	16.92	15.63	1.292	6	8
F TT vs. F TT B1	16.92	14.2	2.717	6	5
F TT B2 vs. F TT B1	15.63	14.2	1.425	8	5
F Crest vs. F Crest B2	34.56	35	-0.4444	9	16
F Crest vs. F Crest B1	34.56	29.17	5.389	9	9
F Crest B2 vs. F Crest B1	35	29.17	5.833	16	9
F TT vs. F Crest	16.92	34.56	-17.64	6	9
F TT B2 vs. F Crest B2	15.63	35	-19.38	8	16
F TT B1 vs. F Crest B1	14.2	29.17	-14.97	5	9

SUPP. FIGURE 4B

	**Supp.Fig.4B**					
	F Crest	F Crest B2	F Crest KN93	F Crest KN93 B2	F ICI	F Crest B1
	0.06	0.083	0.033	0.036	0.034	0.026
	0.06	0.083	0.026	0.036	0.034	0.029
	0.068	0.083	0.026	0.012	0.023	0.046
	0.0374	0.089	0.038	0.015	0.021	0.013
	0.138	0.101	0.022	0.015	0.037	0.04
	0.138	0.101		0.023	0.015	0.04
	0.068	0.101				0.034
	0.085	0.163				0.03
	0.085	0.163				0.023
	0.132	0.104				
	0.132	0.104				
	0.132	0.031				
	0.132	0.064				
	0.132	0.048				
	0.127	0.078				
	0.098	0.078				
	0.098					
	0.098					
No of channels	18	16	5	6	6	9
No of cells	9	9	4	4	5	8
No of animals	7	4	2	2	2	3

Statistical test on No of channels

Kruskal-Wallis test

P value	< 0.0001
Exact or approximate P value?	Approximate
P value summary	****
Do the medians vary signif. (P < 0.05)	Yes
Number of groups	6
Kruskal-Wallis statistic	41.45

Data summary	
Number of treatments (columns)	6
Number of values (total)	60

Dunn's multiple comparisons test	Mean rank diff.	Significant?	P value
F Crest vs. F Crest B2	3.063	No	>0.9999
F Crest vs. F Crest KN93	29.9	Yes	0.0049
F Crest vs. F Crest B1	27.61	Yes	0.0007
F Crest vs. F ICI	31.17	Yes	0.0011
F Crest B2 vs. F Crest KN93 B2	31.27	Yes	0.0013
F Crest KN93 vs. F Crest KN93 B2	4.433	No	>0.9999
F Crest B1 vs. F ICI	3.556	No	>0.9999

Test details	Mean rank 1	Mean rank	Mean rank (	n1	n2
F Crest vs. F Crest B2	44.5	41.44	3.063	18	16
F Crest vs. F Crest KN93	44.5	14.6	29.9	18	5
F Crest vs. F Crest B1	44.5	16.89	27.61	18	9
F Crest vs. F ICI	44.5	13.33	31.17	18	6
F Crest B2 vs. F Crest KN93 B2	41.44	10.17	31.27	16	6
F Crest KN93 vs. F Crest KN93 B2	14.6	10.17	4.433	5	6
F Crest B1 vs. F ICI	16.89	13.33	3.556	9	6

SUPP. FIGURE 5B

	Supp.Fig.5B							
	Co Beta1 M1	Co Beta 2 M1	F Beta 1 M1	F Beta 2 M1				
	0.27575	0.506	0.417	0.476				
	0.3975	0.3985	0.498667	0.611333				
	0.3256667	0.5113333	0.326	0.597				
	0.29225	0.4386667	0.327667	0.557667				
	0.35	0.5376667	0.43425	0.52875				
	0.404	0.555	0.396333	0.55				
	0.30775	0.518	0.369667	0.472667				
	0.417	0.57	0.40275	0.55225				
	0.381	0.4545	0.365	0.628667				
	0.39325	0.43725	0.38175	0.48325				
	0.3895	0.361	0.342	0.37575				
	0.383	0.357	0.483	0.32225				
	0.3115	0.364	0.698333	0.30675				
	0.287667	0.403333	0.64	0.303				
	0.31075	0.411667	0.639667	0.4523333				
	0.3505	0.385667	0.383	0.34975				
	0.28675	0.421667	0.70525	0.3596667				
	0.282333	0.4415	0.63125	0.4606667				
	0.342	0.581	0.496667	0.38375				
	0.2915	0.53125	0.592	0.3786667				
	0.4915	0.52975	0.494333	0.6655				
	0.543333	0.5436667	0.39725	0.52575				
	0.406667	0.4666667	0.504333	0.613667				
	0.48125	0.4335	0.458	0.551				
	0.486	0.42925	0.521333	0.676				
	0.499	0.507	0.58525	0.527333				
	0.5055	0.46075	0.512667	0.708				
	0.46275		0.534333	0.736				
	0.40275		0.468667	0.7285				
	0.654		0.481333	0.715				
			0.586333					
No of cells	30	27	31	30				
No of animals	3	3	3	3				
	**Statistical test on No of cells**							
	**Kruskal-Wallis test**							
	P value			0.0002				
	Exact or approximate P value?			Approximate				
	P value summary			***				
	Do the medians vary signif. (P < 0.05)			Yes				
	Number of groups			4				
	Kruskal-Wallis statistic			20.12				
	Data summary							
	Number of treatments (columns)			4				
	Number of values (total)			118				
	Number of families			1				
	Number of comparisons per family			4				
	Alpha			0.05				
	Dunn's multiple comparisons test			Mean rank diff.	Significant?	P value		
	Co Beta1 M1 vs. Co Beta 2 M1			-26.33	Yes	0.0149		
	F Beta 1 M1 vs. F Beta 2 M1			-8.085	No	>0.9999		
	Co Beta1 M1 vs. F Beta 1 M1			-29.25	Yes	0.0034		
	Co Beta 2 M1 vs. F Beta 2 M1			-11	No	0.9011		
	Test details			Mean rank 1	Mean rank 2	Mean rank diff.	n1	n2
	Co Beta1 M1 vs. Co Beta 2 M1			36.3	62.63	-26.33	30	27
	F Beta 1 M1 vs. F Beta 2 M1			65.55	73.63	-8.085	31	30
	Co Beta1 M1 vs. F Beta 1 M1			36.3	65.55	-29.25	30	31
	Co Beta 2 M1 vs. F Beta 2 M1			62.63	73.63	-11	27	30

SUPP. FIGURE 5C

	Supp.Fig.5C							
	Co Beta 1 M2	o Beta 2 M	F Beta 1 M2	F Beta 2 M2				
	0.308	0.534	0.88	0.685				
	0.6245	0.59975	0.869333	0.447667				
	0.8063333	0.451667	0.834667	0.347				
	0.1665	0.450667	0.751333	0.434333				
	0.8403333	0.386	0.8475	0.50425				
	0.68475	0.328333	0.936667	0.557333				
	0.54525	0.193	0.910333	0.729333				
	0.48725	0.282	0.8315	0.59075				
	0.4695	0.67475	0.892	0.397				
	0.69075	0.74825	0.89475	0.59275				
	0.84775	0.741	0.8715	0.825				
	0.8735	0.7355	0.62375	0.816				
	0.868	0.5925	0.763	0.753				
	0.823	0.675333	0.585	0.8026667				
	0.86425	0.719	0.656	0.8376667				
	0.84675	0.703	0.615667	0.74325				
	0.88775	0.48	0.5035	0.728				
	0.864	0.668	0.39475	0.767				
	0.897333	0.454	0.529	0.8215				
	0.87375	0.659	0.67325	0.797				
	0.35	0.613	0.723	0.39625				
	0.409333	0.458667	0.88025	0.578				
	0.562333	0.585	0.838	0.536				
	0.32625	0.62075	0.7075	0.4755				
	0.337	0.67925	0.848	0.463				
	0.368	0.567667	0.59325	0.489667				
	0.32625	0.5855	0.815333	0.4045				
	0.33275		0.645667	0.41225				
	0.4035		0.807333	0.355				
	0.34375		0.852	0.542				
			0.753667					
No of cells	30	27	31	30				
No of animals	3	3	3	3				
	**Statistical test on No of cells**							
	**Kruskal-Wallis test**							
	P value				0.0002			
	Exact or approximate P value?				Approximate			
	P value summary				***			
	Do the medians vary signif. (P < 0.05)				Yes			
	Number of groups				4			
	Kruskal-Wallis statistic				19.32			
	Data summary							
	Number of treatments (columns)				4			
	Number of values (total)				118			
	Number of families				1			
	Number of comparisons per family				4			
	Alpha				0.05			
	Dunn's multiple comparisons test			Mean rank diff.	Significant?	P value		
	Co Beta 1 M2 vs. Co Beta 2 M2			10.17	No	>0.9999		
	F Beta 1 M2 vs. F Beta 2 M2			30.42	Yes	0.0021		
	Co Beta 1 M2 vs. F Beta 1 M2			-25.59	Yes	0.014		
	Co Beta 2 M2 vs. F Beta 2 M2			-5.333	No	>0.9999		
	Test details			Mean rank 1	Mean rank 2	Mean rank diff.	n1	n2
	Co Beta 1 M2 vs. Co Beta 2 M2			56.33	46.17	10.17	30	27
	F Beta 1 M2 vs. F Beta 2 M2			81.92	51.5	30.42	31	30
	Co Beta 1 M2 vs. F Beta 1 M2			56.33	81.92	-25.59	30	31
	Co Beta 2 M2 vs. F Beta 2 M2			46.17	51.5	-5.333	27	30

SUPP. FIGURE 5D

	**Supp.Fig.5D**			
	Control	Failing		
	1800.416	3725.153		
	3279.152	2737.658		
	1854.35	2364.087		
	3456.74	3316.816		
	1724.166	3204.07		
	2751.547	3767.994		
	2350.725	2657.945		
	1864.878	3686.213		
	1721.378	2190.765		
	1721.968	4032.664		
	2646.991	3912.572		
	2917.699	2575.625		
	2252.582	7006.08		
	2331.087	5042.219		
	3349.098	2641.893		
	2787.597	4186.526		
	3733.958	5220.115		
	2582.514	2644.26		
	3283.346	6169.492		
	1506.373	5242.867		
	2366.75	6231.9		
	1188.063	1862.733		
	2963.867	2617.261		
	4189.334	4000.486		
	2848.722	4249.213		
	1395.929	2788.188		
	3481.556	2936.661		
	2466.953	3484.54		
	1196.561	1622.265		
	1559.181	2602.181		
	3181.665	1947.125		
	3161.47	2859.711		
	2913.301	1403.89		
	2121.939	2887.192		
	3082.263	1540.687		
	3074.317	5272.677		
	2130.323	1875.772		
	2934.364	3804.508		
	1889.167	4656.837		
	2012.055	3082.393		
	2213.565	3090.069		
	1966.421	7128.899		
	1565.691	4007.373		
	3778.959	5687.257		
	2069.296	2809.875		
	3273.746	4809.159		
	2092.687	4086.456		
	2182.055	5530.675		
	2957.763	4180.082		
	2308.011	2541.752		
	3051.072	2220.47		
	3089.032	3005.897		
	2448.554	2925.007		
	2367.248	3280.424		
	2814.116	3216.648		
	2129.681	1511.103		
	3304.366	4350.197		
	2567.984	3510.908		
		3627.561		
		3957.899		
		1826.063		
No of cells	58	61		
No of animals	3	3		
	**Statistical test on No of cells**			
	**Mann Whitney test**			
	P value			< 0.0001
	Exact or approximate P value?			Exact
	P value summary			****
	Significantly different? (P < 0.05)			Yes
	One- or two-tailed P value?			Two-tailed
	Sum of ranks in column A,B			2652 , 448[illegible]
	Mann-Whitney U			941
	Difference between medians			
	Median of column A			2458
	Median of column B			3280
	Difference: Actual			822.7
	Difference: Hodges-Lehmann			854

SUPP. FIGURE 5E

Supp.Fig.5E

	Beta1 Co	Beta 1 F	Beta 2 Co	Beta 2 F	Cav3 Co	Cav3 F
	6047.847	10947.65	6848.995	8031.133	7909.843	13398.34
	7360.552	12787.23	7304.016	8089.012	6629.544	11280.33
	5895.52	9968.032	9210.92	8420.424	8196.026	9293.087
	5598.548	11336.23	7692.716	8602.11	5887.481	9238.197
	10044.52	13344.37	7221.239	9598.739	6642.391	10320
	7856.306	12067.12	6162.492	8956.575	6447.94	11982.65
	5921.279	11230.68	9256.757	6005.324	7637.232	11291.52
	5799.009	11034.12	7845.628	7717.478	7938.22	11246.41
	5501.279	10231.01	6563.51	9432.556	11291.57	9417.6
	4642.089	10268.42	7528.527	9746.794	12164.16	9597.604
	7301.749	11683.95	6726.864	4702.4	10666.25	13597.05
	8098.064	8679.155	7359.66	4775.166	11269.98	13639.41
	6910.496	18934.12	6698.071	3711.048	11067.99	11203.86
	5932.899	14595.27	6630.643	4906.532	10373.46	11273.41
	6573.363	13613.21	6431.718	4993.26	9646.747	13499.59
	4890.087	7138.601	7987.708	4420.952	10013.59	13572.97
	7049.194	14938.33	8236.842	3598.882	10626.31	13833.23
	6196.39	16059.69	7093.902	4001.383	9787.825	11302.75
	5273.094	10674.74	5657.488	3852.717	7446.488	12042.03
	5421.382	14579.23	5172.929	3821.813	9338.989	13089.62
	9939.805	11207.45	6254.84	8845.222	8867.559	13987.87
	13927.2	15742.34	5394.666	7463.198	7594.547	9524.236
	12114.77	13666.85	5565.285	7868.293	10562.81	8507.946
	12648.86	15302.34	5722.744	8307.697	9402.41	6762.935
	10087.8	13700.8	5843.511	7455.195	6934.416	8386.354
	11625.73	14160.84	5783.352	9584.258	8808.479	8428.835
	12187.57	17268.05	5092.268	8175.595	7246.495	7570.947
	9065.355	13275.15	5084.229	9671.865	7895.43	6193.588
	9003.505	10988.55		11290.31	11709.41	8118.269
	12312.53	12741.12		12838.99	12201.36	7502.496
		18906.21			11083.64	7693.995
					11114.29	7472.46
					10908.92	11041.51
					8981.959	10448.27
					11494.67	8761.901
					10788.19	6342.759
					8420.337	12112.92
					9173.834	10989.16
					9441.98	9629.385
					8567.555	10249.7
					9657.203	9823.379
					10029.47	11065.81
					8730.903	10093.88
					9793.674	10411.1
					8971.903	9071.85
					8123.688	10611.83
					8829.337	10632.85
					8595.257	10372.11
					7835.896	8224.978
					12184.73	9062.986
					11801.83	11316.38
					9398.887	7712.505
					10253.68	7388.977
					11368.53	8518.657
					10439.27	8399.853
					7855.33	8193.753
					8047.083	8750.289
					9306.263	6793.46
						7414.556
						7966.157
						9771.353
No of cells	30	31	28	30	58	61
No of animals	3	3	3	3	3	3

Statistical test on No of cells

Kruskal-Wallis test

P value	< 0.0001
Exact or approximate P value?	Approximate
P value summary	****
Do the medians vary signif. (P < 0.05)	Yes
Number of groups	6
Kruskal-Wallis statistic	94.53
Data summary	
Number of treatments (columns)	6
Number of values (total)	238
Number of families	1
Number of comparisons per family	5
Alpha	0.05

Dunn's multiple comparisons test	Mean rank diff.	Significant?	P value
Beta1 Co vs. Beta 1 F	-109.9	Yes	<0.0001
Beta 2 Co vs. Beta 2 F	-26.55	No	0.7108
Cav3 Co vs. Cav3 F	-12.32	No	>0.9999
Beta1 Co vs. Beta 2 Co	39.12	No	0.1529
Beta 1 F vs. Beta 2 F	122.4	Yes	<0.0001

Test details	Mean rank 1	Mean rank 2	Mean rank diff.	n1	n2
Beta1 Co vs. Beta 1 F	88.3	198.2	-109.9	30	31
Beta 2 Co vs. Beta 2 F	49.18	75.73	-26.55	28	30
Cav3 Co vs. Cav3 F	128.1	140.5	-12.32	58	61
Beta1 Co vs. Beta 2 Co	88.3	49.18	39.12	30	28
Beta 1 F vs. Beta 2 F	198.2	75.73	122.4	31	30

SUPP. FIGURE 5F

	Supp.Fig.5F							
	Ratio B1 Co	Ratio B1 F	Ratio B2 Co	Ratio B2 F				
	0.812174	0.805149	0.865883	0.599413				
	0.788153	0.937521	1.101737	0.71709				
	0.664841	0.889696	1.123828	0.906095				
	0.73718	1.005572	1.306623	0.931146				
	0.950933	0.988502	1.087145	0.93011				
	0.835563	0.889055	0.95573	0.747462				
	0.853897	0.811862	1.212057	0.531844				
	0.658344	0.976233	0.988336	0.686217				
	0.759164	0.849608	0.581275	1.001588				
	0.587946	0.78447	0.618911	1.015545				
	0.62358	0.835292	0.630668	0.49373				
	0.663702	1.161486	0.653032	0.56126				
	0.623486	1.714813	0.605175	0.548733				
	0.533808	1.396908	0.639193	0.585061				
	0.602568	1.553682	0.666724	0.592402				
	0.544434	1.125473	0.797687	0.583936				
	0.613258	1.233256	0.775137	0.581066				
	0.574368	1.461412	0.724768	0.492886				
	0.626233	1.108559	0.599184	0.513525				
	0.590961	1.422406	0.603781	0.496727				
	1.268496	1.140896	0.647686	1.146868				
	1.143004	1.422611	0.537881	1.010045				
	1.026516	1.353974	0.637424	0.923654				
	1.345783	1.46981	0.584331	0.989029				
	0.983822	1.510254	0.651312	0.909863				
	1.022624	1.334439	0.711912	1.095308				
	1.167473	1.624028	0.576744	1.203451				
	1.154039	1.279889	0.591516	1.304443				
	1.118853	1.335997		1.417284				
	1.323037	1.405841		1.313942				
		1.670694						
No of cells	30	31	28	30				
No of animals	3	3	3	3				
	**Statistical test on No of cells**							
	**Kruskal-Wallis test**							
	P value			< 0.0001				
	Exact or approximate P value?			Approximate				
	P value summary			****				
	Do the medians vary signif. (P < 0.05)			Yes				
	Number of groups			4				
	Kruskal-Wallis statistic			33.49				
	Data summary							
	Number of treatments (columns)			4				
	Number of values (total)			119				
	Number of families			1				
	Number of comparisons per family			4				
	Alpha			0.05				
	Dunn's multiple comparisons test			Mean rank diff.	Significant?	P value		
	Ratio B1 Co vs. Ratio B1 F			-36.85	Yes	0.0001		
	Ratio B2 Co vs. Ratio B2 F			-4	No	>0.9999		
	Ratio B1 Co vs. Ratio B2 Co			8.567	No	>0.9999		
	Ratio B1 F vs. Ratio B2 F			41.42	Yes	<0.0001		
	Test details			Mean rank 1	Mean rank 2	Mean rank diff.	n1	n2
	Ratio B1 Co vs. Ratio B1 F			53.57	90.42	-36.85	30	31
	Ratio B2 Co vs. Ratio B2 F			45	49	-4	28	30
	Ratio B1 Co vs. Ratio B2 Co			53.57	45	8.567	30	28
	Ratio B1 F vs. Ratio B2 F			90.42	49	41.42	31	30

SUPP. FIGURE 6A

Supp.Fig.6A

	F Crest	F Crest 48I	F Crest REM
Occurrence (%)	27.3	35.9	17.1
Total No of recordings	55	39	41
Recordings with channels	15	14	7
Recordings wihout channels	40	25	34

Table Analyzed	F Crest vs F Crest 48H		
**Fisher's exact test**			
P value	0.497		
P value summary	ns		
One- or two-sided	Two-sided		
Statistically significant? (alpha<0.05)	No		
Data analyzed	Channel	No Channe	Total
Row 1	15	40	55
Row 2	14	25	39
Total	29	65	94

Table Analyzed	F Crest 48H vs F Crest REM		
**Fisher's exact test**			
P value	0.0759		
P value summary	ns		
One- or two-sided	Two-sided		
Statistically significant? (alpha<0.05)	No		
Data analyzed	Channel	No Channe	Total
Row 1	14	25	39
Row 2	7	34	41
Total	21	59	80

Table Analyzed	F Crest 48H vs F Crest REM		
**Fisher's exact test**			
P value	0.0759		
P value summary	ns		
One- or two-sided	Two-sided		
Statistically significant? (alpha<0.05)	No		
Data analyzed	Channel	No Channe	Total
Row 1	14	25	39
Row 2	7	34	41
Total	21	59	80

SUPP. FIGURE 6B

	Supp.Fig.6B							
	F Crest	F Crest 48H	F Crest REM					
	0.06	0.129	0.073					
	0.06	0.129	0.073					
	0.068	0.129	0.033					
	0.0374	0.026	0.082					
	0.138	0.051	0.014					
	0.138	0.051	0.014					
	0.068	0.051	0.011					
	0.085	0.035	0.011					
	0.085	0.035	0.023					
	0.132	0.092	0.088					
	0.132	0.092						
	0.132	0.163						
	0.132	0.163						
	0.132	0.163						
	0.127	0.163						
	0.098	0.163						
	0.098	0.123						
	0.098	0.073						
		0.136						
		0.018						
		0.023						
		0.03						
		0.03						
		0.05						
		0.101						
		0.101						
No of channels	18	26	10					
No of cells	9	14	7					
No of animals	4	8	5					
	**Statistical test on No of channels**							
	**Kruskal-Wallis test**							
	P value			0.0034				
	Exact or approximate P value?			Approximate				
	P value summary			**				
	Do the medians vary signif. (P < 0.05)			Yes				
	Number of groups			3				
	Kruskal-Wallis statistic			11.37				
	Data summary							
	Number of treatments (columns)			3				
	Number of values (total)			54				
	Number of families			1				
	Number of comparisons per family			3				
	Alpha			0.05				
	Dunn's multiple comparisons test			Mean rank diff.	Significant?	P value		
	F Crest vs. F Crest 48H			4.784	No	0.9621		
	F Crest 48H vs. F Crest REM			15.78	Yes	0.0209		
	F Crest vs. F Crest REM			20.56	Yes	0.0027		
	Test details			Mean rank 1	Mean rank 2	Mean rank diff.	n1	n2
	F Crest vs. F Crest 48H			33.61	28.83	4.784	18	26
	F Crest 48H vs. F Crest REM			28.83	13.05	15.78	26	10
	F Crest vs. F Crest REM			33.61	13.05	20.56	18	10
