# Supplementary material for: Functional LTCC-β2AR Complex Needs Caveolin-3 and Is Disrupted in Heart Failure
Source: Circ Res. 2023 Jun 14;133(2):120–37. doi: 10.1161/CIRCRESAHA.123.322508 (PMC10321517; doi:10.1161/CIRCRESAHA.123.322508)
Supplement: Supplementary file 2 [file res-133-120-s002.pdf]

SUPPLEMENTAL MATERIAL

Functional LTCC-β_2AR complex needs caveolin-3 and is disrupted in heart failure

Jose L. Sanchez-Alonso, Laura Fedele, Jaël S. Copier, Carla Lucarelli, Catherine Mansfield, Aleksandra Judina, Steven R. Houser, Thomas Brand, and Julia Gorelik.

METHODS

Cell isolation

Rat cardiomyocytes

Cardiomyocytes were obtained from adult male Sprague-Dawley rats (250-300g). A rat model of 16-weeks post coronary artery ligation was used to represent the features of chronic heart failure, including ventricular dilatation, reduced ejection fraction, as well as several structural, molecular, and electrophysiological changes already characterized[61]. Female rats are excluded from this work as the heart failure model was developed and characterize in male rats. Briefly, rats were anesthetized with 2.5% isoflurane, intubated, and ventilated after preoperative buprenorphine (0.03 mg/kg subcutaneous) injection. The thorax was shaved and sterilized with 2% w/v chlorhexidine gluconate in 70% v/v isopropyl alcohol. A left thoracotomy was performed at the 4th intercostal space, the pericardium opened, and the left anterior descending coronary artery was ligated using a 7/0 prolene suture. Sixteen weeks later, left ventricle systolic and diastolic dimensions, and left ventricle ejection fraction were recorded.

Ventricular cardiomyocyte isolation was done as previously described[2]. Rats were anesthetized with 5% isoflurane and then killed by cervical dislocation. Hearts were fast extracted and placed in Tyrode solution containing: 140 mM NaCl, 6 mM KCl, 1mM MgCl2, 1mM CaCl2, 10mM glucose and 10mM HEPES, adjusted to pH 7.4 with 2 mmol/L NaOH. Hearts were placed in a Langendorff system after aortic cannulation, and perfused with Tyrode solution for 5 min, then with low Ca^{2+} oxygenated solution for 5 min: 120mM NaCl, 5.4mM KCl, 5mM MgSO4, 5mM sodium pyruvate, 20mM glucose, 20mM taurine, 10mM HEPES, 5mM nitrilotriacetic acid, and 0.04mM CaCl2, adjusted to pH 6.96 with 2 mmol/L NaOH, and finally for 10 min with enzyme solution: 120mM NaCl, 5.4mM KCl, 5mM MgSO4, 5mM sodium pyruvate, 20mM glucose, 20mM taurine, 10mM HEPES, and 0.2mM CaCl2, pH 7.4 with collagenase type 2 (1 mg/ml; Worthington) and hyaluronidase (0.6 mg/ml; Sigma-Aldrich).

Left ventricular cardiomyocytes were plated on dishes coated with laminin and allowed to attach to the dish/coverslip for at least 45 minutes before experiments. Cardiomyocytes were used on the same day of isolation. Cells were washed twice with the external recording solution and mounted on the microscope stage for recordings. Cells isolated from the 16-week post-coronary artery ligation model were

considered “failing” cells. These failing cells are isolated from the left ventricular wall of the infarcted heart after the infarcted area is removed.

Mouse cardiomyocytes

Cav3 conditional KO mice were generated by crossing a mouse line with loxP-flanked exon 2 of Cav3 with an alpha-myosin heavy chain proto-oncogene tyrosine-protein kinase MER Cre recombinase proto-oncogene tyrosine-protein kinase MER (αMHC-MERCreMER) mouse producing a tamoxifen-inducible, cardiac-specific Cav3 KO mouse[52]. The Cav3 conditional KO mice line was kindly provided by Professor Timothy Kamp. Mice were given an intraperitoneal injection of 20 mg/kg tamoxifen/day for 5 consecutive days and sacrificed 21 days post-tamoxifen treatment.

Adult male mice were anesthetized with 5% isoflurane and then killed by cervical dislocation. The thorax cavity was opened, the heart removed and placed in ice cold Krebs-Henseleit buffer: 119mM NaCl, 4.7mM KCl, 0.94mM MgSO4, 1mM CaCl2, 1.2mM KH2PO4, 25mM NaHCO3, and 11.5mM glucose, pH 7.4. The aorta was cannulated and attached to the Langendorff apparatus. The heart was retrogradely perfused at 37°C with Krebs-Henseleit buffer for 1 min. The perfusate was switched to low-Ca^{2+} oxygenated buffer: 120mM NaCl, 5mM KCl, 5mM MgSO4, 5mM Na pyruvate, 20mM glucose, 20mM taurine, 10mM HEPES, and 5mM nitrilotriacetic acid, 0.035mM $CaCl_2$, pH: 6.9, for 4 minutes. The heart was subsequently perfused for 1 min with enzyme buffer solution containing proteinase type XXIV (0.36 mg/ml, Sigma-Aldrich, P8038-1G) and then for 4-5 min with collagenase type V (1mg/ml, Sigma-Aldrich, C9263-1G). Heart was then removed from the cannula, and the left ventricle triturated in the enzyme buffer. The solution was passed through a gauze to remove any undigested tissue. The enzyme buffer was composed of 120mM NaCl, 5mM KCl, 5mM MgSO4, 5mM Na pyruvate, 20mM glucose, 20mM taurine, 10mM HEPES, and 0.035mM $CaCl_2$ (pH: 7.4). For whole-cell current experiments Ca^{2+} was gradually re-introduced.

Left ventricular cardiomyocytes were plated on dishes coated by laminin and allowed to attach to the dish/coverslip for at least 45 minutes before experiments. Cardiomyocytes were used on the same day of isolation. Cells were washed twice with the external recording solution and mounted on the microscope stage for recordings.

Human cardiomyocytes

Ventricular cardiomyocytes from failing and donor hearts were isolated as previously described from the apical section of the posterior-lateral left ventricle free wall by enzymatic digestion[40]. End-stage heart failure was caused by dilated cardiomyopathy (n=13 patients, average age 48 ± 10 years, 8 males and 5 females). Clinical characteristics of the heart failure patients used in this work can be found in Table S1. Tissue samples from the same anatomical region from donor hearts (n=4, average age 36 ± 13 years, 2 males and 2 females) not suitable for transplantation were used

as control. None of the donors had a history of cardiothoracic disease. The reasons for these hearts being rejected for transplantations were degeneration on the organ care system prior to transplantation; heart size/logistics; and increased lactate and K+.

Dissected samples were kept in ice-cold calcium free Krebs-Ringer saline solution (in g/L): 7.012 NaCl, 0.402 KCl, 1.332 MgSO4, 0.55 Pyruvate, 3.603 Glucose, 2.502 Taurine, 2.383 HEPES, 1.286 Nitrilotriacetic Acid; pH = 6.96. Approximately 0.5g of myocardial wall was taken, connective and adipose tissue was removed, and the cardiac muscle was cut with razor blades in small cubes of similar size (1-2 mm^3). The cubes were transferred to fresh calcium free Krebs-Ringer solution at 37^oC and washed 3 times for 3 min each. After this, the tissue samples were incubated in 10 ml of Krebs-Ringer solution (in g/L): NaCl 7.012, KCl 0.402, MgSO4 1.332, Pyruvate 0.55, Glucose 3.603, Taurine 2.502, HEPES 2.383; pH = 7.4, adding 200nM CaCl2 and Proteinase type XXIV (0.36mg/ml; Sigma-Aldrich) under mechanical agitation. After 25 min, the partially digested tissue was transferred to 10 ml of fresh Krebs-Ringer in which proteinase was substituted by collagenase type V (1mg/ml; Sigma-Aldrich) continuing at 37^oC under agitation. Every 10-15 min the tissue was transferred to a fresh solution while the remaining solution contained single isolated cardiomyocytes. Supernatants were centrifuged for 3 min at 600 rpm after each incubation step, and the cell pellets were re-suspended in 2-3 mL of Krebs-Ringer solution without enzymes.

All cardiomyocytes were plated on laminin coated dishes and allowed to attach for at least 45 minutes before experiments. Cardiomyocytes were used on the same day of isolation.

Electrophysiology

Super-resolution scanning patch-clamp of LTCC

Topographical surface images and single L-type calcium channels recordings were obtained by SICM and cell-attached patch-clamp as previously described[2]. Briefly, after a surface image is obtained with a high resistance pipette, a clipping of the tip is performed to increase the area[34], the pipette is then move to a specific microdomain of interest, T-tubule or crest, and a recording is made under cell-attach configuration.

Experiments were performed at room temperature using the following solutions: external solution containing: 120mM K-gluconate, 25mM KCl, 2mM $MgCl^2$, 1mM $CaCl^2$, 2mM EGTA, 10mM Glucose, 10mM HEPES, pH 7.4 with NaOH; internal recording solution containing: 90mM $BaCl^2$, 10mM HEPES, 10mM Sucrose, pH 7.4 with TEA-OH. The pipette used for cell attached recordings after clipping had an average resistance of 28.19±0.22 MΩ. Currents were recorded using Axopatch 200A amplifier (Axon Instruments, Foster City, CA, USA), controlled and monitored using pClamp software version 10 (Axon Instruments). Single channels were sampled at 10 kHz and filtered at 2 kHz (-3 dB, 8-pole Bessel). Analysis was performed using Clampfit version 10.2. Liquid junction potential was calculated as -16.7 mV and corrected for all the data shown in this work. Single LTCCs were identified and

characterized by their voltage dependent properties. The holding membrane potential was held at -96.7 mV. A protocol of incremental step of 10mV with pulses from -36.7 to +23.3mV was applied 3 times to generate the current-voltage (I-V) relationship when a channel was present. For the study of the open probability 150 pulses at the same -6.7mV step voltage were recorded. All the sweeps were checked for the presence of LTCCs. Occurrence of LTCCs was calculated as the percentage of recording with LTCC activity versus the total number of recordings.

The Po was averaged from a minimum of 20 sweeps at -6.7 mV for each cell. Each cell was recorded only once. The total number of channels in the recording was input into pClamp software to calculate the single Po of one single channel. To estimate the number of channels per seal a carefully examination of all the recordings per cell was done. The level of the spikes determined the number of channels present in the seal. When only a single level of spikes was observed that seal was considered to contain only one channel.

The conductance was determined by plotting the average amplitude of all the openings in the recording against the test potential for every single experiment. The slopes from the linear relationships of these I/V plots were calculated as the conductance for that single channel.

The human samples contribution to each subset of data is represented on the Tables S2 to S5.

$I_{Ca,L}$ Whole cell current recordings

L-type Ca^{2+} currents ($I_{Ca,L}$) were recorded using the whole-cell patch clamp technique using a Multiclamp amplifier (Molecular Devices, Sunnyvale, CA, USA) and a Digidata 1322A A/D converter (Axon instruments). Cardiomyocytes were super fused at room temperature in external solution containing: 140mM NaCl, 6mM KCl, 10mM Glucose, 10mM HEPES, 1mM $MgCl^2$, 2mM $CaCl^2$ (for mouse cells) or 1mM $CaCl^2$ (for rat cells) adjusted to pH 7.4 with NaOH. Patch pipettes (3-5 MΩ) were filled with internal solution containing: 110mM Cs methanesulfonate,10mM NMDG, 10mM HEPES, 1mM $MgCl_2$, 2.5mM Mg^-ATP, 0.05mM Tris-GTP, 0.5mM $CaCl_2$, 20mM TEA-Cl, 5mM EGTA, adjusted to pH 7.2.

Cell capacitance, series resistance and input resistance were assessed from transient current changes induced by a 10mV step from a holding potential of -50 mV (100 ms). Series resistances was compensated to 70% for each cell and monitored throughout each experiment; deviations >20 % resulted in the data being excluded from further analysis.

The protocol employed to elicit the $I_{Ca,L}$ was: from a holding potential of -80mV, cells were depolarised to -40mV for (900 ms) followed by 500 ms steps from -50mV to +45mV in 5mV increments. Steady-state inactivation of I_{CaL} was assessed with double-pulse protocols in which the conditioning pulses of 500ms duration were followed by a 10ms step to -40 mV and a 200ms step to +5 mV (Fig. S1D).

The amplitude of $I_{Ca,L}$ was measured as the difference between the peak inward current and the current at the end of the depolarizing pulse, normalized to cell

capacitance and expressed as current density (pA/pF). The activation curve was plotted using the test voltage and the corresponding normalized conductance (G/G_{max}). Conductance (G) was calculated using the following equation: $G = I/(V - V_{rev})$, where V was the test voltage, I was the peak current at the test voltage, and V_{rev} was the reversal potential obtained by extrapolating the tail part of the *I*-*V* curve to its intersection with the voltage axis.

The decay time constants (tau_{slow} and tau_{fast}) were determined by fitting the decay phase of the $I_{Ca,L}$ current resulting from -40mV to a 0mV voltage step with a double exponential equation. The two decay constants were calculated from the equation: tau_{fast} and the tau_{slow}, which represent, respectively, the fast and slow phase of inactivation.

Pharmacological treatments

The following drugs were used for the treatment of cardiomyocytes: Isoproteranol (ISO, I6504, Sigma) 0.1μM for western blot experiments and 1μM for whole-cell $I_{Ca,L}$ and single LTCC recordings; ICI 118.551 0.05μM (ICI, 0821/10, Tocris); CGP-20712A 0.3μM (CGP, C231, Sigma); H89 dihydrochloride 10μM (H-89, 2910, Tocris); PKA inhibitory peptide 3μM (PKAi, 12-151, Merck); KN 93 phosphate 10μM (KN-93, 5215/1, Tocris); Autocamtide 2 related inhibitory peptide 5μM (AIP, ALX-151-030-MC05, Enzo); Methyl-beta-cyclodextrin 1mM (MβCD, 332615, Sigma).

Western Blot

Cardiomyocytes isolated on the day were plated in 60mm coated laminin dishes by duplicates or triplicates and let to attach for 45 minutes. Afterwards, medium was washed, and new medium was added with the specific drug (treated) or without (control), and dishes were incubated at 37ºC for 15 minutes. Cells were then scraped, centrifuged at 4ºC and washed 3 times. Finally, supernatant was removed, and cell pellets were snap-frozen. All the samples were lysed with RIPA buffer (Sigma) containing 1mg/ml protease inhibitor and 1mM dithiothreitol (DTT). The samples were then sonicated for 1 min, at 4 °C and left for 15 min gentle agitation. Lastly, the samples were spun down and the supernatant was stored at -80°C for further use. The protein concentration was determined using a Pierce BCA Protein Assay Kit (Thermofisher Scientific). Samples containing 30 micrograms of protein were loaded with LDS loading buffer, denatured at 70°C for 10 min and resolved on 10% SDS polyacrylamide gels. Proteins were separated by SDS-PAGE and then transferred onto a PVDF membrane and blocked with 5 % BSA in Tris-Buffered saline with 5% Tween for 1-hour at room temperature. The membranes were incubated with the corresponding primary antibodies and left in a rocker at 4°C overnight, rinsed 3 times with tris-buffered saline plus 0.5% Tween 20 (TBST) and probed with the secondary antibody for 1-hour, at room temperature followed by three TBST washes. The following primary antibodies were used: Phospholamban (pThr17) (1:1000, anti-rabbit, A010-13, Badrilla), Phospholamban (pSer16) (1:1000, anti-rabbit, A010-12, Badrilla), pCaMKII

(1:1000, anti-mouse, MA1-047, Invitrogen), Cav3 (1:500, anti-mouse, 610421, BD Bioscience), GAPDH (1:1000, anti-rabbit, CST2118, Cell Signalling Technology), and α-actinin (1:1000, anti-mouse; Sigma-Aldrich #A7811). On the next day, the membranes were incubated at room temperature with the corresponding secondary antibodies for 2-hours. Alexa Fluor 488 donkey anti-mouse (1:1000, A21202, Thermo Fisher Scientific), Alexa Fluor 546 donkey anti-mouse (1:1000, A10036, Thermo Fisher Scientific), Alexa Fluor 488 donkey anti-rabbit (1:1000, A11008, Thermo Fisher Scientific,), and Alexa Fluor 546 donkey anti-rabbit (1:1000, A10040, Thermo Fisher Scientific) were used as the secondary antibodies. Fluorescent bands were detected at 488 and 546nm wavelength by Chemi-Doc (Bio-Rad). To normalize pPLN expression to the total PLN, membranes were incubated in stripping buffer (15g glycine, 1g SDS, 10 ml Tween20 in 1L, adjusted to ph 2.2) for 2-3 hours, rinse 3 times in TBST, and reproved with Phospholamban primary antibody (1:1000, anti-mouse, A010-14, Badrilla). Bands were detected following the same protocol as before.

Protein band intensity was normalized to the corresponding α-actinin or GAPDH intensity, measured using ImageJ software. Data plotted for each treated group were normalize to their corresponding untreated group.

Immunostaining

For immunostaining, cardiomyocytes on coverslips were fixed with 4% formaldehyde for 10 min, permeabilized using 0.3% Triton X-100 for 15 min and blocked with 5% goat serum in PBS for 1 hour and 30 min at room temperature. Incubation with primary antibodies was done in blocking buffer overnight at 4°C. The following antibodies were used: β_1AR (1:100, anti-rabbit, AAR-023, Alomone), β_2AR (1:100, anti-rabbit, AAR-016, Alomone) Cav3 (1:100, anti-mouse, 610421, BD Bioscience). Subsequent incubation with secondary antibodies Alexa Fluor anti-rabbit 488 (1:1000, A11008, Thermo Fisher Scientific) and Alexa Fluor Donkey anti-mouse 546 (1:1000, A10036, Thermo Fisher Scientific) was performed in blocking buffer for 1-hour at room temperature. Images were taken with a Zeiss LSM-780 inverted confocal microscope. A lack of unspecific binding was confirmed using secondary antibodies without primary antibodies on control adult rat cardiomyocytes following the same procedures.

Staining mean intensity was calculated by normalising the area covered by the signal to the area of the cell. Each cell value presented is an average of 3 to 4 z-stack images. To accurately select the area of each cell, area was automatically calculated with the Trainable Weka Segmentation ImageJ plugin[62].

From the same images the Mander's coefficients were calculated using the JACoP ImageJ plugin[28]. Image A was assigned for the 546nm channel (Cav3) and image B for the 488nm channel (β_1AR or β_2AR). Images are automatically thresholded by the software, and the M1 and M2 coefficients were obtained.

Statistics

All statistical analysis and graphs were performed using GraphPad prism. To test for normality a Shapiro-Wilk test was used. The statistical test used for each set of data is indicated in each figure legend, and details of the tests and all the data can be found in the Supplemental Stats File. Most of the data presented in this work failed to pass the normality test and the corresponding non-parametric test was used. All data are expressed as mean ± standard error of the mean (SEM). $p<0.05$ was considered statistically significant. Representative images of mean/average values were selected to represent as best as possible the results.

Statistical limitations

The statistical analyses used in this work are restricted because of the nature of the experimental preparations that include the use of human tissue and the experimental questions being addressed.

While hierarchical statistical approaches are now becoming more widely implemented to deal with concerns about reproducibility and pseudoreplication, they require two main conditions to be applicable, a normal distribution of the values for each subgroup, and a similar SD between all subgroups.

Firstly, our data does not fulfil these requirements. Secondly, we are aiming to describe differences in channel activities in two different microdomains. In our single-channel recording study, one value is plotted per channel recorded. It is reasonable to consider that the activity of a single channel recorded from the t-tubule area of the membrane (TT) or from the crest area of the membrane (Crest) will not be representative of the activities of the total population of channels for that cell.

However, when one considers that the LTCC channels appear to behave differently in the t-tubule and crest areas, then by plotting one value per cell, (when we only record each cell once and at different microdomains (TT or Crest)) our ability to discriminate the behaviours will be lost when one of the intentions of the work is to test our hypothesis that there are differences in activities in the two microdomains

The important point is that we are not comparing between animals. Statistical tests have been performed between the same cell type with or without treatment. No hypotheses are tested that compare control versus failing cells because this is not the focus of the work.

We are confident in the reliability of the electrophysiological results because we have tested the effect of β2AR local stimulation on single LTCC activities in three different experimental preparations – rat, mouse, and human. The same result was obtained, supporting the lack of a false positive effect due to pseudoreplication.

SUPP. FIGURES

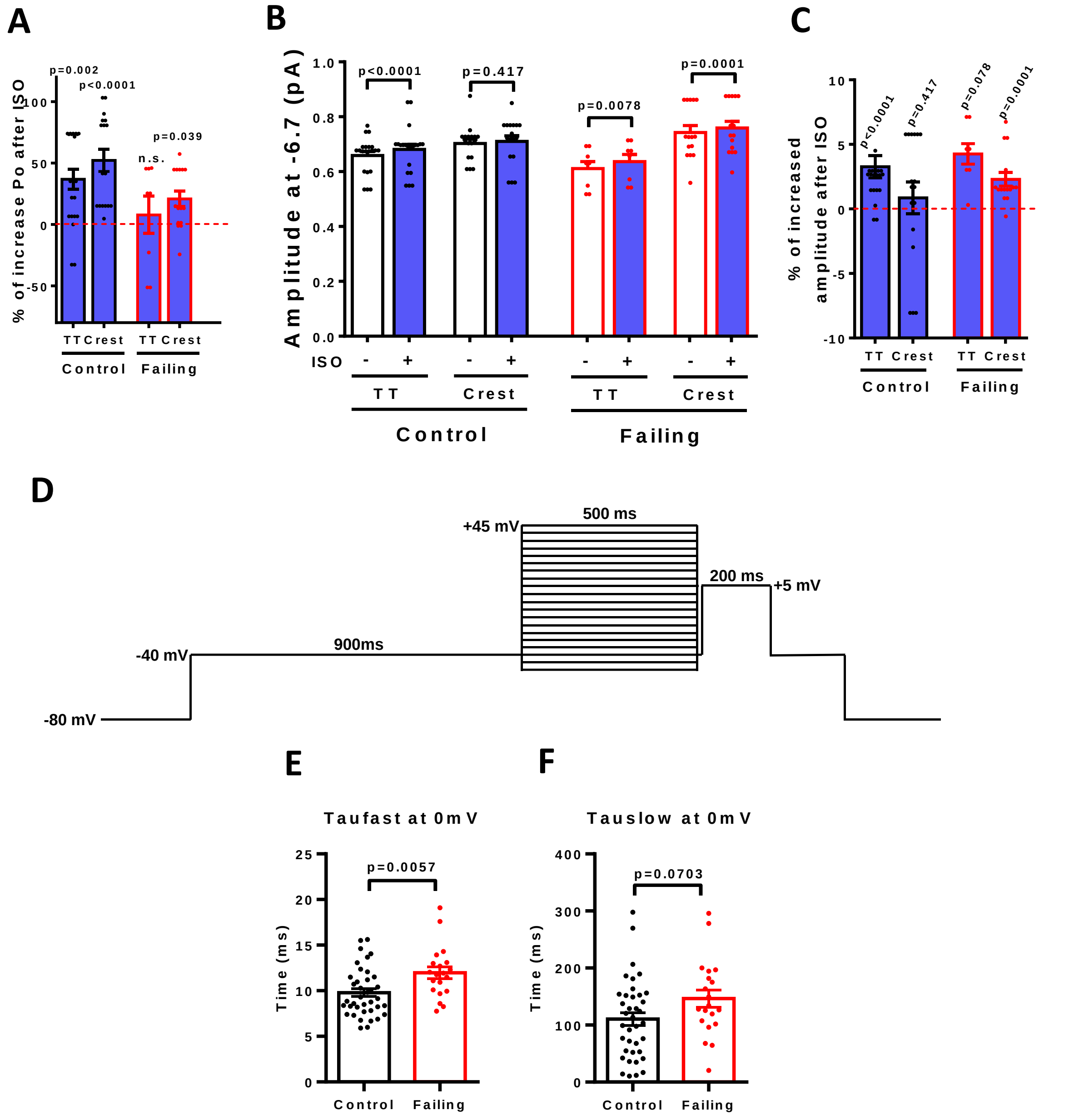

Figure S1. LTCCs response to isoproterenol stimulation on control and failing cardiomyocytes. A) Percentage of increased Po from control and failing cardiomyocytes in TT and Crest before and after 1µM ISO application (n=channels/cells/animals TT control 21/8/6, Crest control 17/7/5, TT failing 8/5/4, Crest failing 15/7/7, p-values by Wilcoxon signed rank test). **B)** Summary graph of the amplitude at -6.7mV step from control and failing cardiomyocytes in TT and Crest before and after 1µM ISO application (n=channels/cells/animals TT control 21/8/6, Crest control 17/7/5, TT failing 8/5/4, Crest failing 15/7/7, p-values by Wilcoxon matched-pairs signed rank test). **C)** Percentage of increased Amplitude from B (p-values by Wilcoxon signed rank test). **D)** Schematic of the whole-cell voltage protocol to elicit $I_{Ca,L}$. **E)** Tau$_{fast}$: Values: Control:9.539 ±0.48 (SEM); Failing: 11.98 ± 0.63; **F)**

Tau$_{slow}$: Ctrl:111.4 ± 10.91; Failing: 146.4 ± 15.09. (n=cells/animals, control: 38/11, failing: 20/4, p-values by Mann-Whitney test)

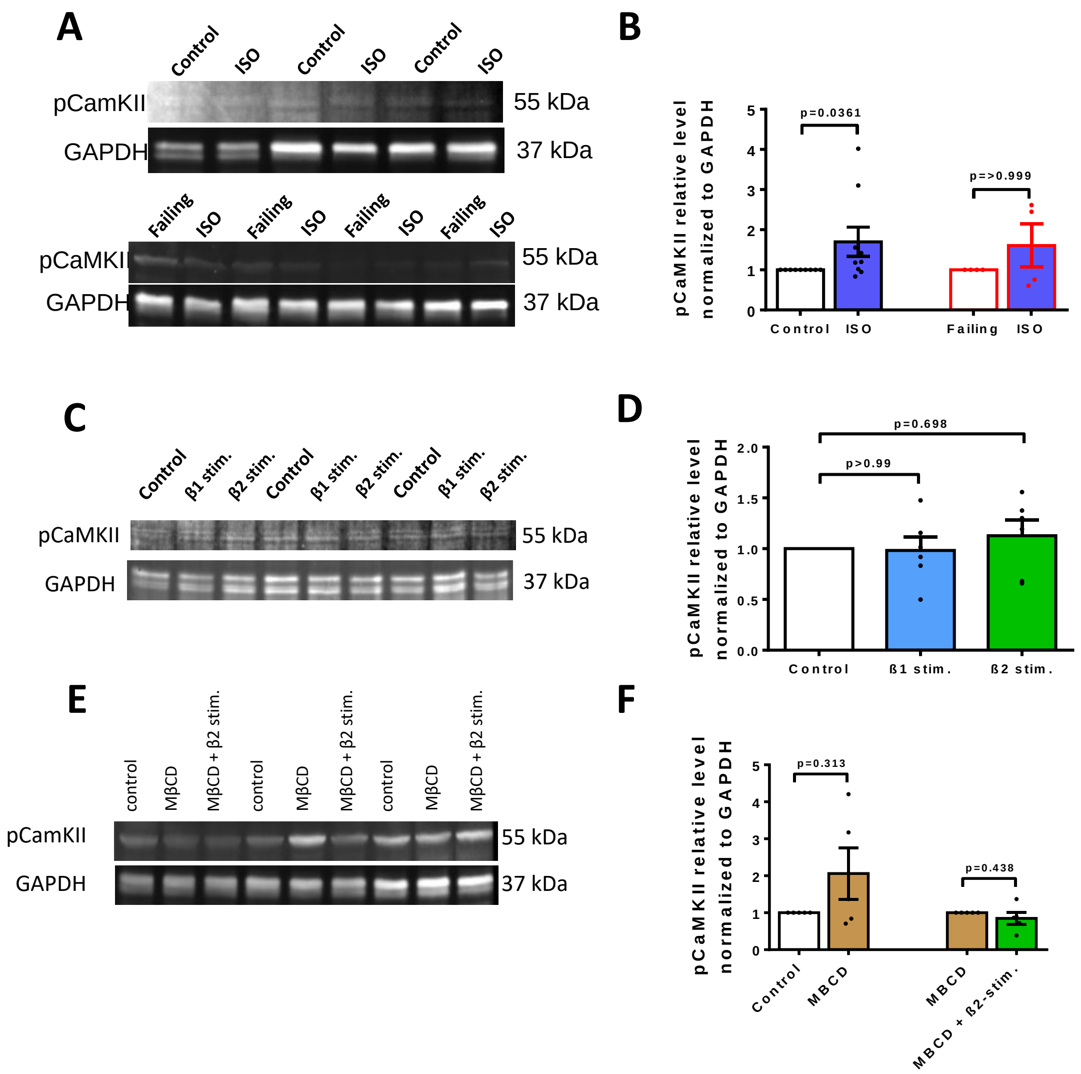

Figure S2. WB analisys of pCaMK expresion. A) Representative WBs of control and failing samples with or without ISO treatment. **B)** Summary graph of densitometry analysis of pCaMKII normalized to GAPDH from control (n=9) and failing (n=4) cardiomyocytes (p-values by Mann-Whitney test). **C)** Representative WBs of control cells under no stimulation, under β_1AR stimulation, or under β_2AR stimulation. **D)** Summary graph of densitometry analysis of pCaMKII normalized to GAPDH from control cardiomyocytes after β_1AR or β_2AR stimulation (n=5, p-values Kruskal-Wallis test followed by Dunn's multiple comparisons test). **E)** Representative WBs of control cardiomyocytes treated with MβCD with or without β_2AR stimulation. **F)** Summary graph of densitometry analysis of pCaMKII normalized to GAPDH from control cardiomyocytes after MβCD with or without β_2AR stimulation (n=6, p-values by Wilcoxon matched-pairs signed rank test).

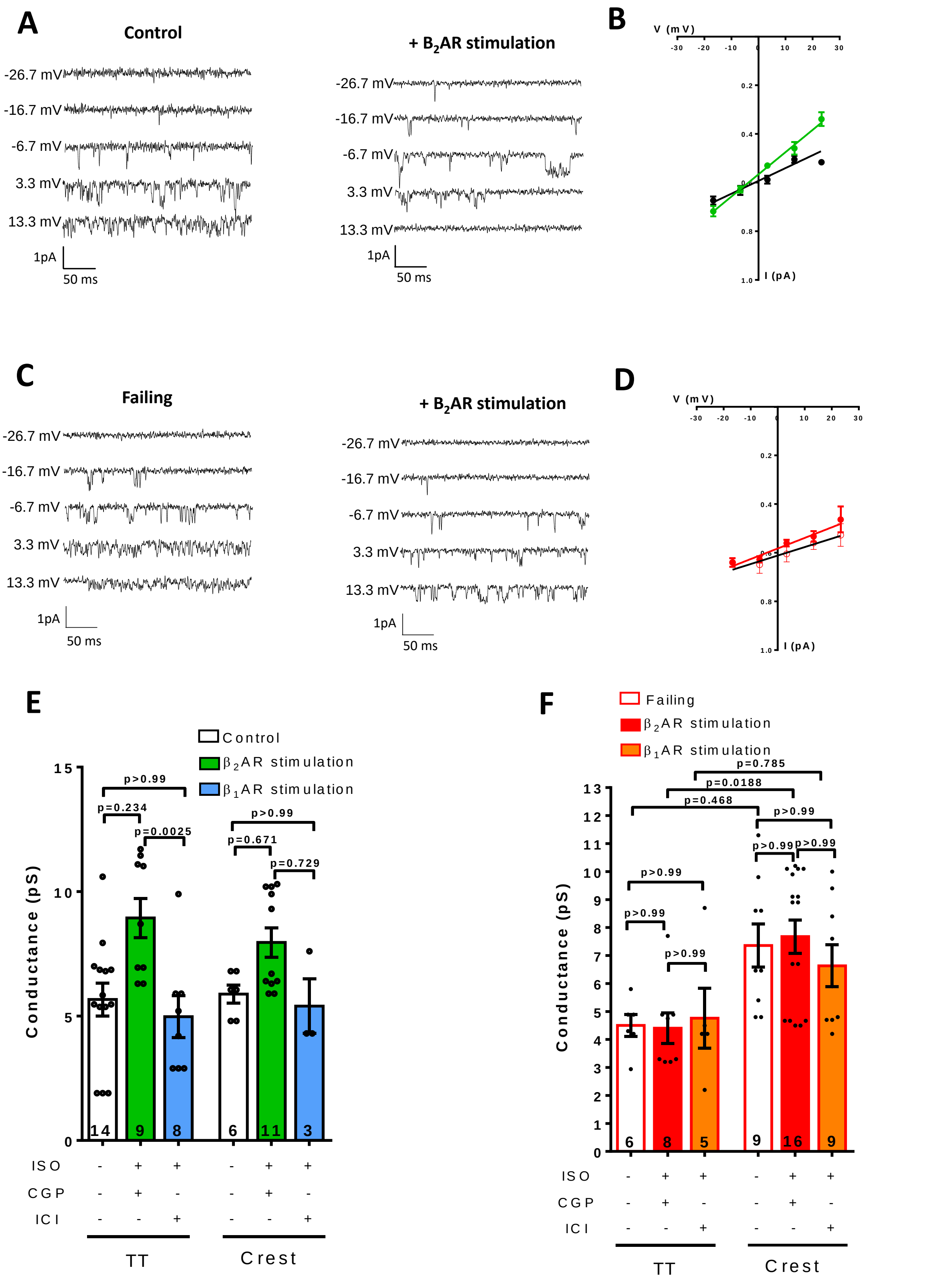

Figure S3. Conductance analysis of single LTCCs. A) Representative traces of LTCC from control cells with or without β2AR stimulation. **B)** I/V graph of LTCCs from control cells. **C)** Representative traces of LTCC from failing cells with or without β2AR stimulation. **D)** I/V graph of LTCCs from failing cells. **E)** Summary graph of the

conductance anlysis of control cells under β_1AR or β_2AR local stimulation (n=channels/cells/animals, TT: 14/9/7, 9/7/6, 8/5/3; Crest: 6/3/3, 11/9/5, 3/2/2, *p<0.05, **p<0.01, by Kruskal-Wallis followed by Dunn's multiple comparisons test). **F)** Summary graph of the conductance anlysis of failing cells under β_1AR or β_2AR local stimulation (n=channels/cells/animals, TT: 6/5/3, 8/5/4, 5/4/2; Crest: 9/6/3, 16/8/5, 9/8/3, *p<0.05, by Kruskal-Wallis followed by Dunn's multiple comparisons test).

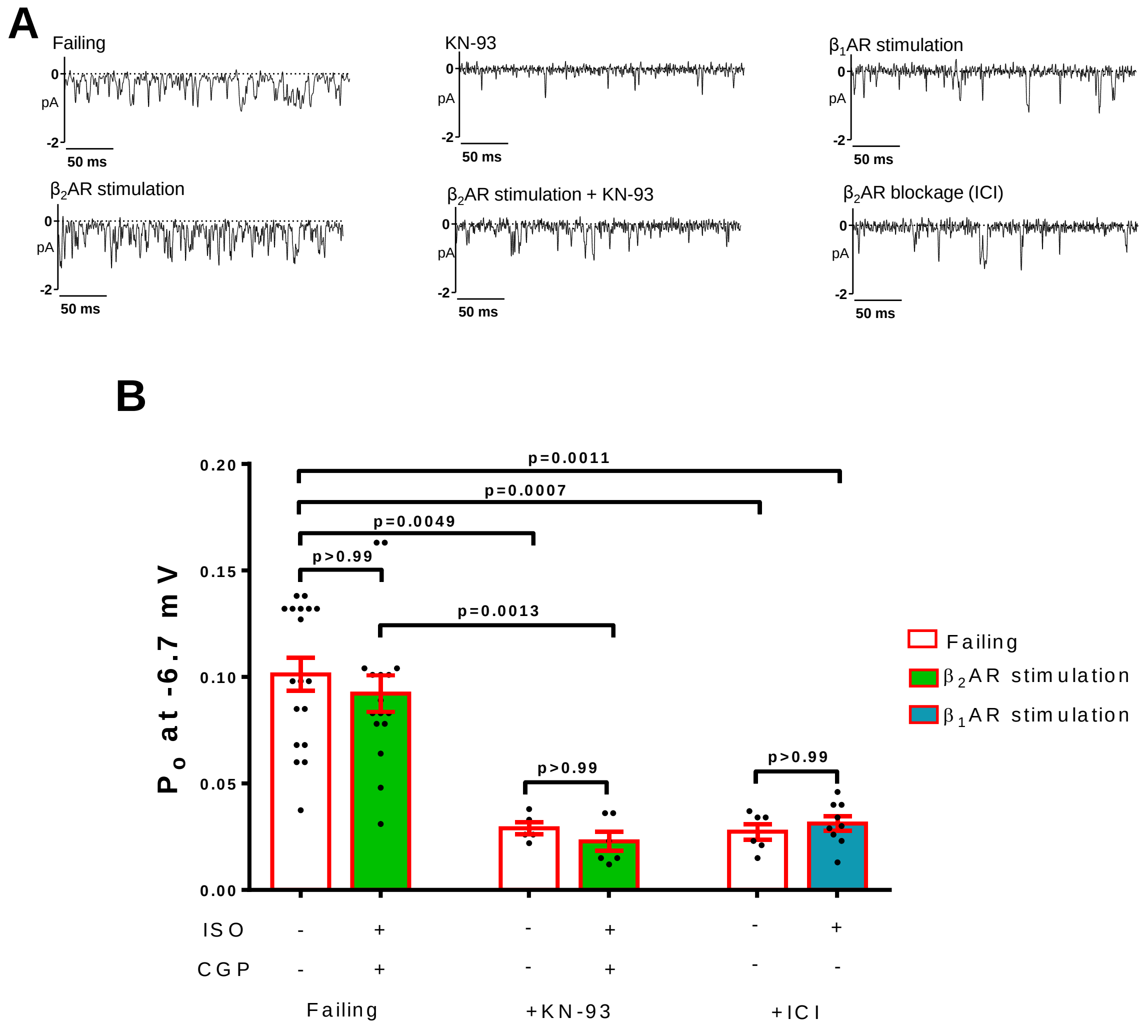

Figure S4. Pathological failing LTCC in the crest domain can be blocked by KN-93 or ICI. A) Representative traces of crest LTCCs at -6.7mV from failing cells under β_1AR stimulation, β_2AR stimulation, CaMKII inhibition (KN-93), and β_2AR inhibition (ICI). **B)** Summary graph of the Po (n=channels/cells/animals: 18/9/7, 16/9/4; 5/4/2, 6/4/2; 6/5/2, 9/8/3, **p<0.01, ***p<0.001, by Kruskal-Wallis followed by Dunn's multiple comparison).

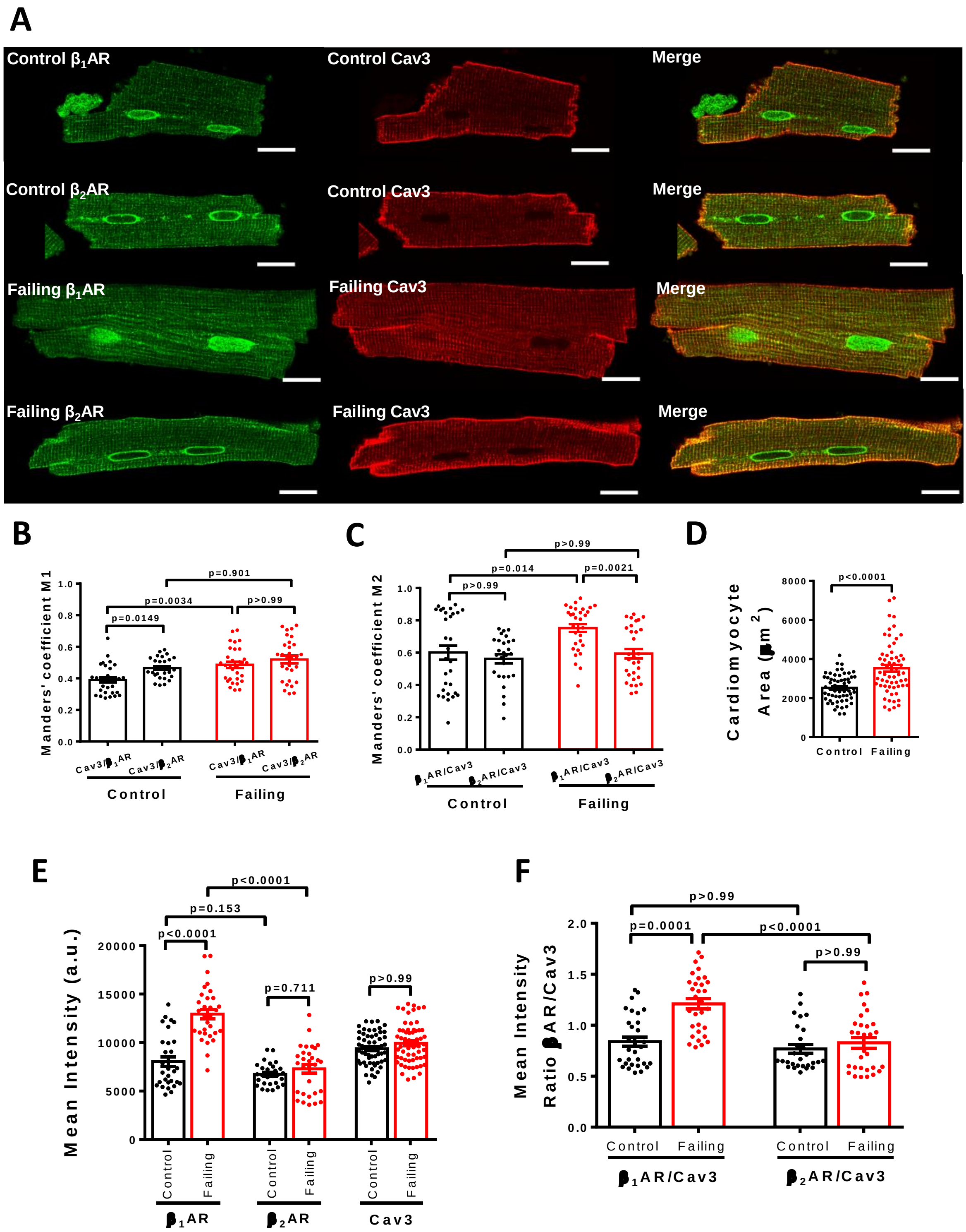

Figure S5. Immunostaining analysis of βAR and Cav3 on control and failing cells. A) Representative confocal images of control and failing cells. Scale bar 10µm. **B)** Mander's coefficient M1 representing the percentage of total Cav3 that co-occurrence with βARs (n=cells/animals: 30/3, 27/3, 31/3, 30/3, p-values by Kruskal-Wallis test followed by Dunn's multiple comparisons test). **C)** Mander's coefficient M2 representing the percentage of total βARs that co-occurrence with Cav3 n=cells/animals: 30/3, 27/3, 31/3, 30/3, p-values by Kruskal-Wallis test followed by

Dunn's multiple comparisons test. **D)** Cardiomyocyte average area (n=cells/animals: 58/3, 61/3, p-value by Mann Whitney test). **E)** Average mean intensity summary graph calculated from the total area of each cell (n=cells/animals: 30/3, 31/3, 28/3, 30/3, 58/3, 61/3, p-values by by Kruskal-Wallis test followed by Dunn's multiple comparisons test **F)** Ratio of β_1AR/Cav3 and β_2AR/Cav3 from the values in F.(p-values by Kruskal-Wallis followed by Dunn's multiple comparisons test)

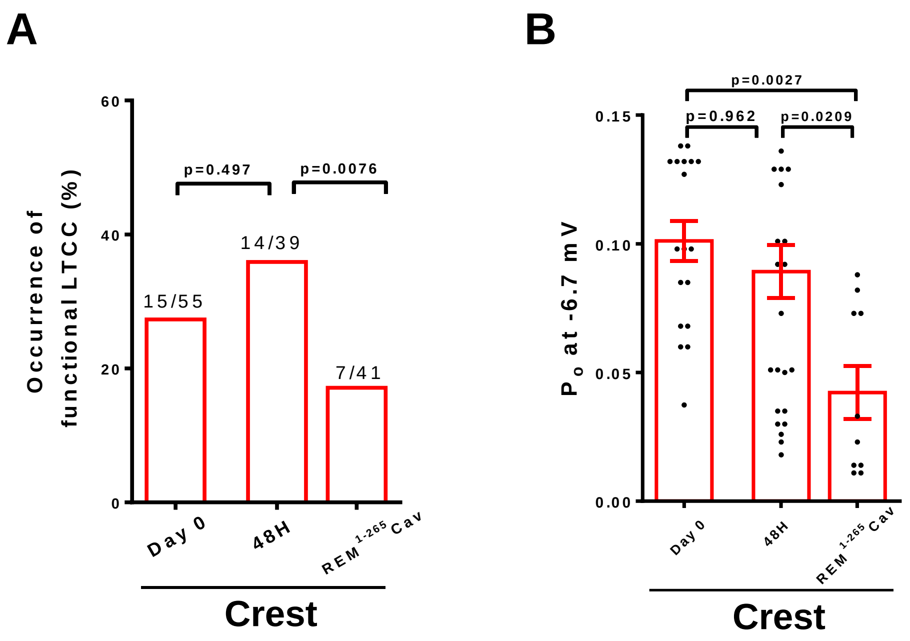

Figure S6. REM$^{1\text{-}265}$Cav reduces by half the available channels in the crest domain of failing cells and blocks the hyperactive LTCCs. A) Summary graph of the chance of obtaining a LTCC current (% of occurrence) (p-values by Fisher's exact test). **B)** Summary graph of the Po from failing cardiomyocytes in crest on fresh isolated cells (day 0), after 48hours of culture, or after 48hours of culture overexpressing REM$^{1\text{-}265}$Cav (n=channels/cells/animals: 18/9/7, 26/14/8, 10/7/5, *$p<0.05$, **$p<0.01$, by Kruskal-Wallis followed by Dunn's multiple comparison).

SUPP. TABLES

Characteristics of DCM patients	
Total number of patients	13
Age, year (mean ± SD)	48±10
Men, n (%)	8 (62)
Women, n (%)	5 (38)
Surgical procedure	
Coronary artery bypass surgery	0 (0)
Coronary artery bypass grafting	0 (0)
Percutaneous coronary intervention	0 (0)
Medical history	
Previous myocardial Infarction	0 (0)
Diabetes mellitus	1 (7.5)
Hypertension	2 (15)
Ex-smoker	7 (54)
Alcohol (more than 5 units per week)	3 (23)
Medications	
Antiplatelets	10 (77)
Diuretics	11 (85)
Aldosterone antagonist	11 (85)
β-blockers	11 (85)
Statins	1 (7.5)
Ca^{2+} channel blockers	0 (0)
Antiarrhythmics	6 (46)

Table S1. Clinical characteristics of DCM patients. Values are total numbers of patients with percentages in parentheses unless indicated otherwise.

	TT		Crest		
	-	+	-	+	ISO
	-	+	-	+	CGP
DN001	✓	✗	✗	✗	
DN002	✓	✗	✗	✗	
DN003	✓	✗	✓	✗	
DN004	✗	✓	✗	✓	

Table S2. Donor human samples contribution to each subset of data on Figure 7B&C.

	TT		Crest		
	-	+	-	+	ISO
	-	+	-	+	CGP
DCM 1	✓	✗	✗	✗	
DCM 2	✓	✗	✗	✗	
DCM 3	✓	✗	✗	✗	
DCM 4	✓	✗	✓	✗	
DCM 5	✓	✗	✓	✗	
DCM 6	✓	✗	✓	✗	
DCM 7	✓	✗	✓	✗	
DCM 8	✗	✗	✓	✗	
DCM 9	✗	✗	✓	✗	
DCM 10	✗	✗	✓	✗	
DCM 11	✗	✓	✗	✗	
DCM 12	✗	✗	✗	✓	
DCM 13	✗	✓	✗	✓	

Table S3. DCM human samples contribution to each subset of data on Figure 7E&D.

	TT		Crest		
	-	+	-	+	ISO
	-	+	-	+	CGP
DN001	✓	✗	✗	✗	
DN002	✓	✗	✗	✗	
DN003	✓	✗	✓	✗	
DN004	✗	✓	✗	✓	

Table S4. Donor human samples contribution to each subset of data on Figure 7G.

	TT		Crest		
	-	+	-	+	ISO
	-	+	-	+	CGP
DCM 1	✓	✗	✗	✗	
DCM 2	✓	✗	✗	✗	
DCM 4	✓	✗	✗	✗	
DCM 5	✓	✗	✓	✗	
DCM 8	✗	✗	✓	✗	
DCM 9	✗	✗	✓	✗	
DCM 11	✗	✓	✗	✗	
DCM 12	✗	✗	✗	✓	
DCM 13	✗	✗	✗	✓	

Table S5. DCM human samples contribution to each subset of data on Figure 7I.
