# Supplementary material for: Functional LTCC-β2AR Complex Needs Caveolin-3 and Is Disrupted in Heart Failure
Source: Circ Res. 2023 Jun 14;133(2):120–37. doi: 10.1161/CIRCRESAHA.123.322508 (PMC10321517; doi:10.1161/CIRCRESAHA.123.322508)
Supplement: Supplementary file 3 [file res-133-120-s003.pdf]

# Major Resources Table

In order to allow validation and replication of experiments, all essential research materials listed in the Methods should be included in the Major Resources Table below. Authors are encouraged to use public repositories for protocols, data, code, and other materials and provide persistent identifiers and/or links to repositories when available. Authors may add or delete rows as needed.

## Genetically Modified Animals

| | Species | Vendor or Source | Background Strain | Other Information | Persistent ID / URL |
|---|---|---|---|---|---|
| **Cav3KO** | Mice | University of Wisconsin Madison | C57BL/6j | Cardiac-specific conditional Caveolin-3 knock-out mice | |
| | | | | | |

## Antibodies

| Target antigen | Vendor or Source | Catalog # | Working concentration | Persistent ID / URL Persistent ID / URL |
|---|---|---|---|---|
| Phospholamban (pThr17) | Badrilla | A010-13 | 1:1000 | Badrilla A010-13 |
| Phospholamban (pSer16) | Badrilla | A010-12 | 1:1000 | Badrilla A010-12 |
| pCaMKII | Invitrogen | MA1-047 | 1:1000 | Invitrogen MA1-047 |
| Caveolin-3 | BD Bioscience | 610421 | 1:500 | BD Bioscience 610421 |
| GAPDH | Cell signalling | CST2118 | 1:1000 | CellSignalling CST2118 |
| α-actinin | Sigma-Aldrich | A7811 | 1:1000 | Sigma A7811 |
| Phospholamban | Badrilla | A010-14 | 1:1000 | Badrilla A010-14 |
| $\beta_1$-AR | Alomone | AAR-023 | 1:100 | Alomone AAR-023 |
| $B_2$-AR | Alomone | AAR-016 | 1:100 | Alomone AAR-016 |
| Alexa Fluor 488 donkey anti-mouse | Thermo Fisher Scientific | A21202 | 1:1000 | ThermoFisher A21202 |
| Alexa Fluor 546 donkey anti-mouse | Thermo Fisher Scientific | A10036 | 1:1000 | ThermoFisher A10036 |
| Alexa Fluor 488 donkey anti-rabbit | Thermo Fisher Scientific | A11008 | 1:1000 | ThermoFisher A11008 |
| Alexa Fluor 546 donkey anti-rabbit | Thermo Fisher Scientific | A10040 | 1:1000 | ThermoFisher A10040 |

DOI [to be added]

**Other**

| Description | Source / Repository | Persistent ID / URL |
|---|---|---|
| Collagenase type 2 | Worthington, LS004177 CLS-2 | Collagenase |
| Hyaluronidase | Sigma-Aldrich, H3506-1G | Hyaluronidase |
| Laminin | Trevigen, 3446-005-01 | Laminin |
| Collagenase type V | Sigma-Aldrich, C9263-1G | Collagenase type V |
| Proteinase type XXIV | Sigma-Aldrich, P8038-1G | Proteinase |
| Isoproteranol | Sigma-Aldrich, I6504 | Isoproteranol |
| ICI 118.551 | Tocris, 0821/10 | ICI |
| CGP-20712A | Sigma-Aldrich, C231 | CGP |
| H89 dihydrochloride | Tocris, 2910 | H89 |
| PKA inhibitory peptide | Merck, 12-151 | PKAi |
| KN-93 | Tocris, 5215/1 | KN-93 |
| Autocamtide 2 related inhibitory peptide (AIP) | Enzo, ALX-151-030-MC05 | AIP |
| Methyl-beta-cyclodextrin (MβCD) | Sigma-Aldrich, 332615 | MBCD |
| RIPA buffer | Sigma-Aldrich, R0278-50ML | RIPA |
| Dithiothreitol (DTT) | Roche, 10197777001 | DTT |
| Pierce BCA Protein Assay Kit | Thermofisher Scientific, 23227 | Protein BCA assay |

DOI [to be added]
