# Supplementary material for: Functional LTCC-β2AR Complex Needs Caveolin-3 and Is Disrupted in Heart Failure
Source: Circ Res. 2023 Jun 14;133(2):120–37. doi: 10.1161/CIRCRESAHA.123.322508 (PMC10321517; doi:10.1161/CIRCRESAHA.123.322508)

Full unedited gels for Figure 2A

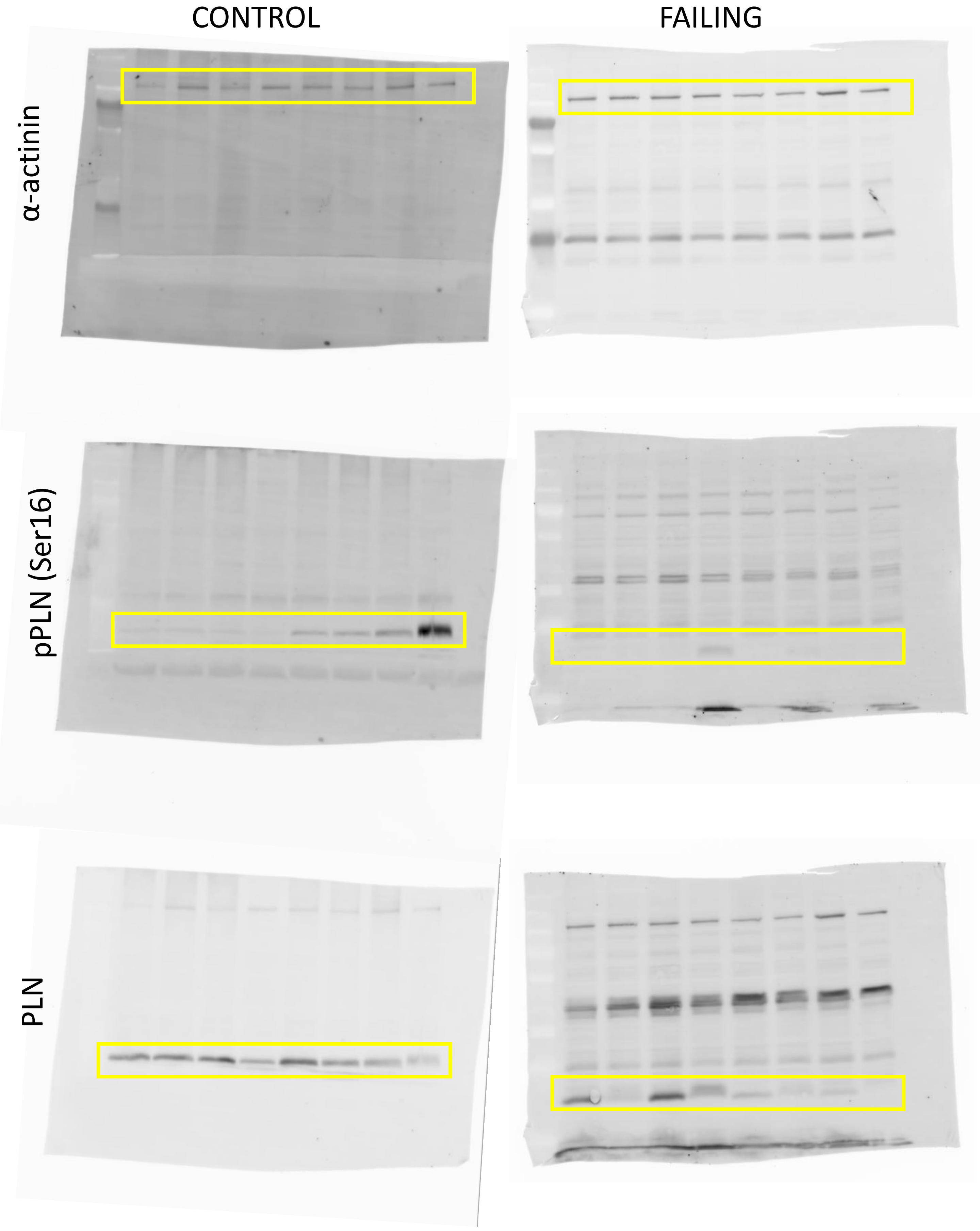

Full unedited gels for Figure 2A

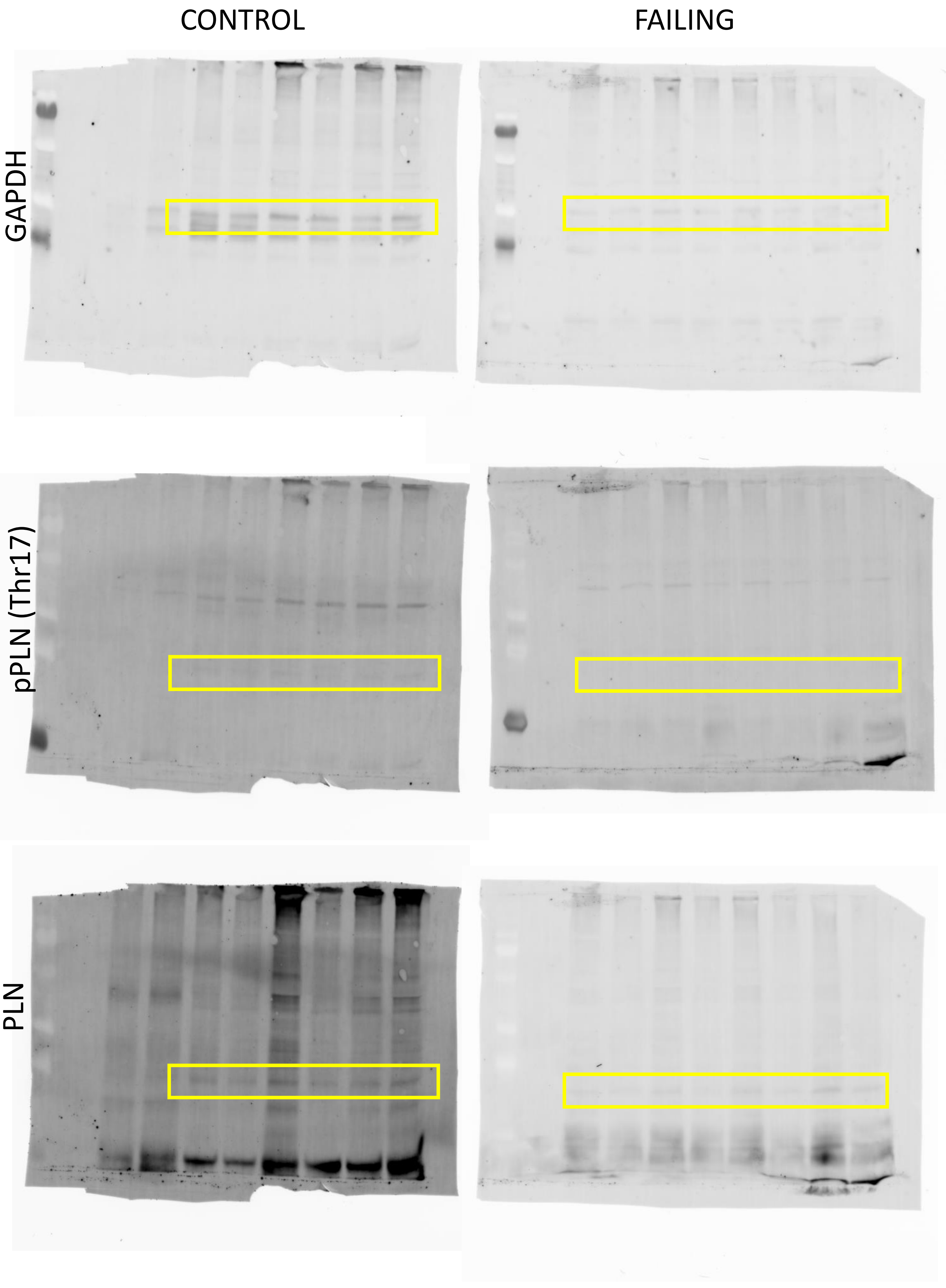

Full unedited gels for Figure 2D

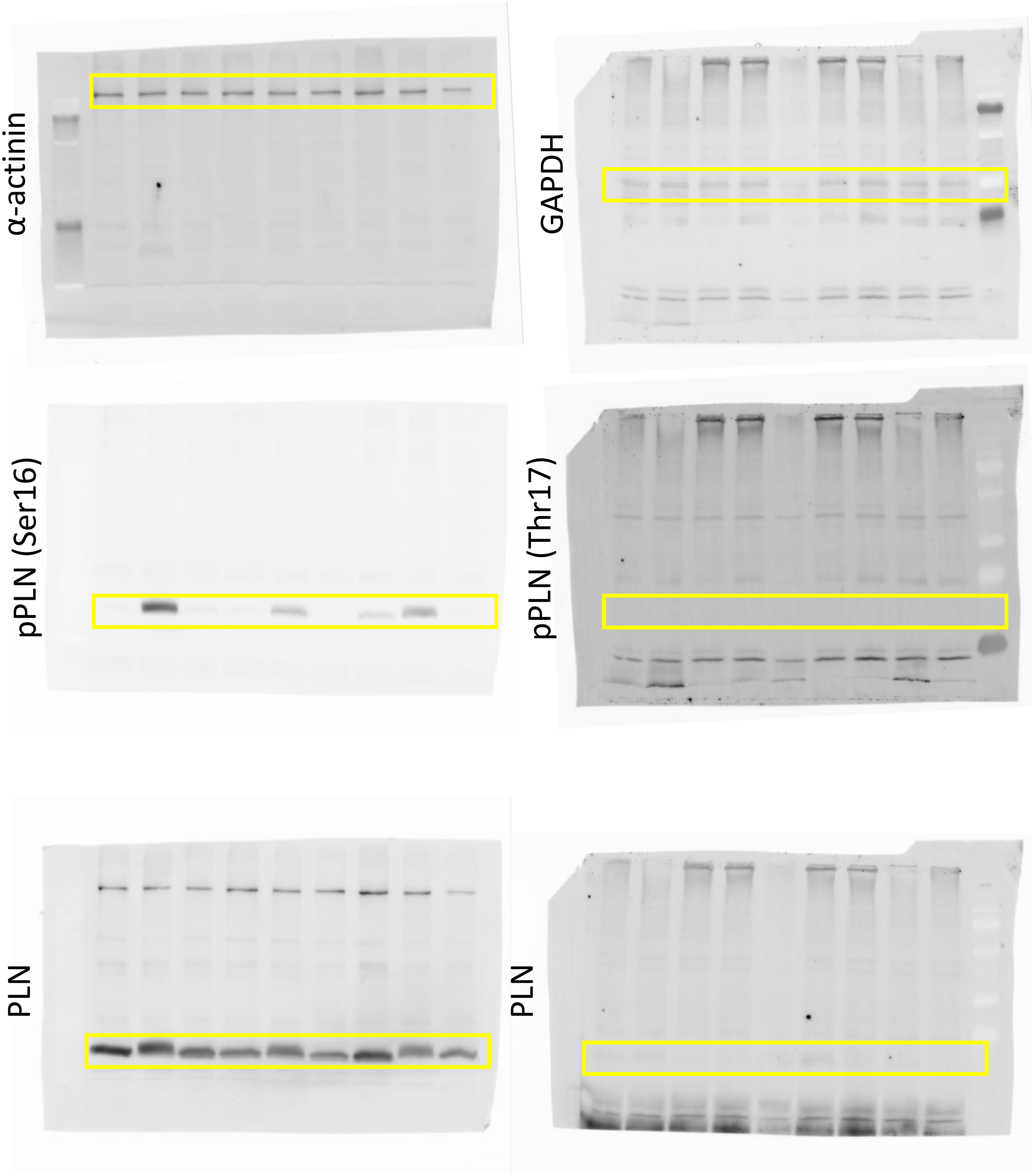

Full unedited gels for Figure 4C

α-actinin

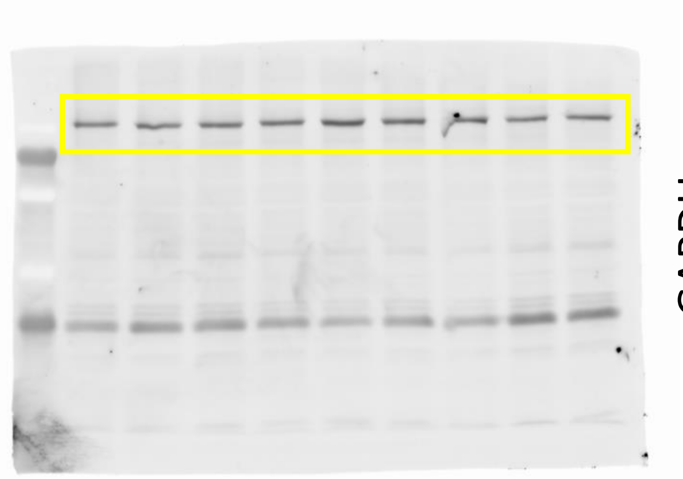

GAPDH

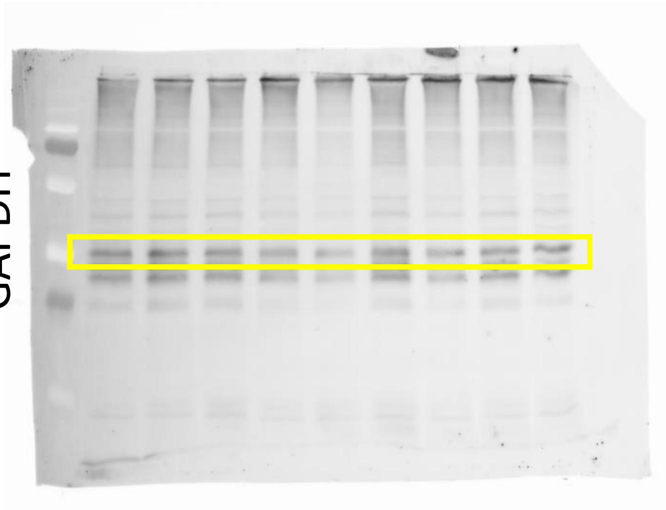

pPLN (Ser16)

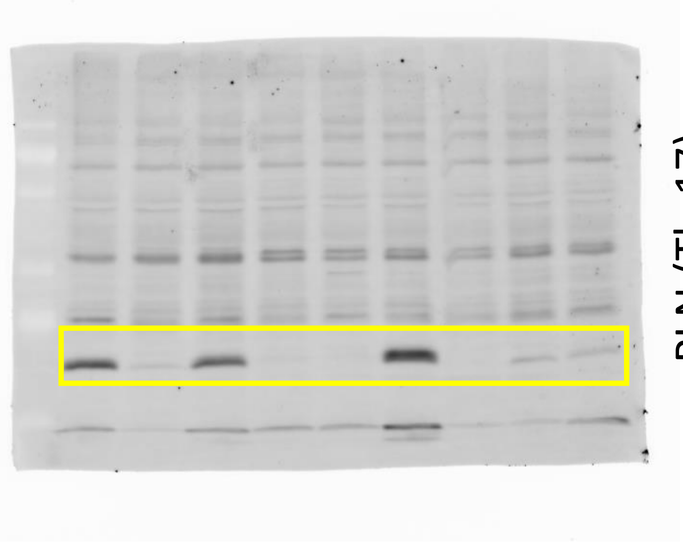

pPLN (Thr17)

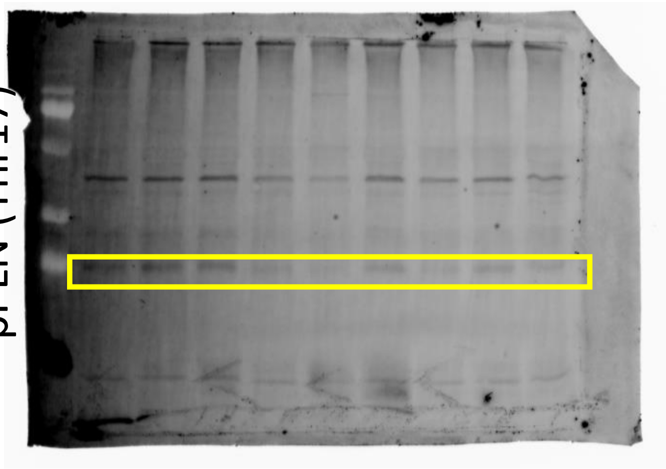

PLN

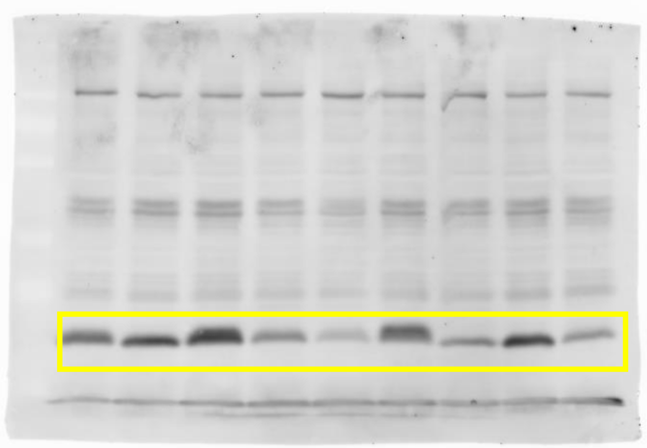

PLN

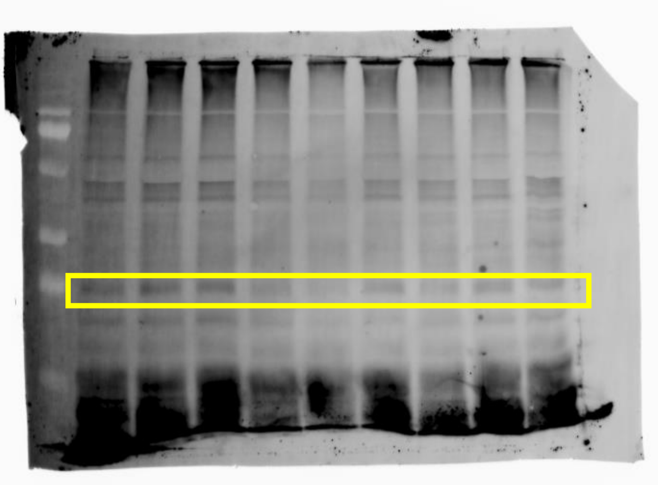

Full unedited gels for Figure 6A

Caveolin-3

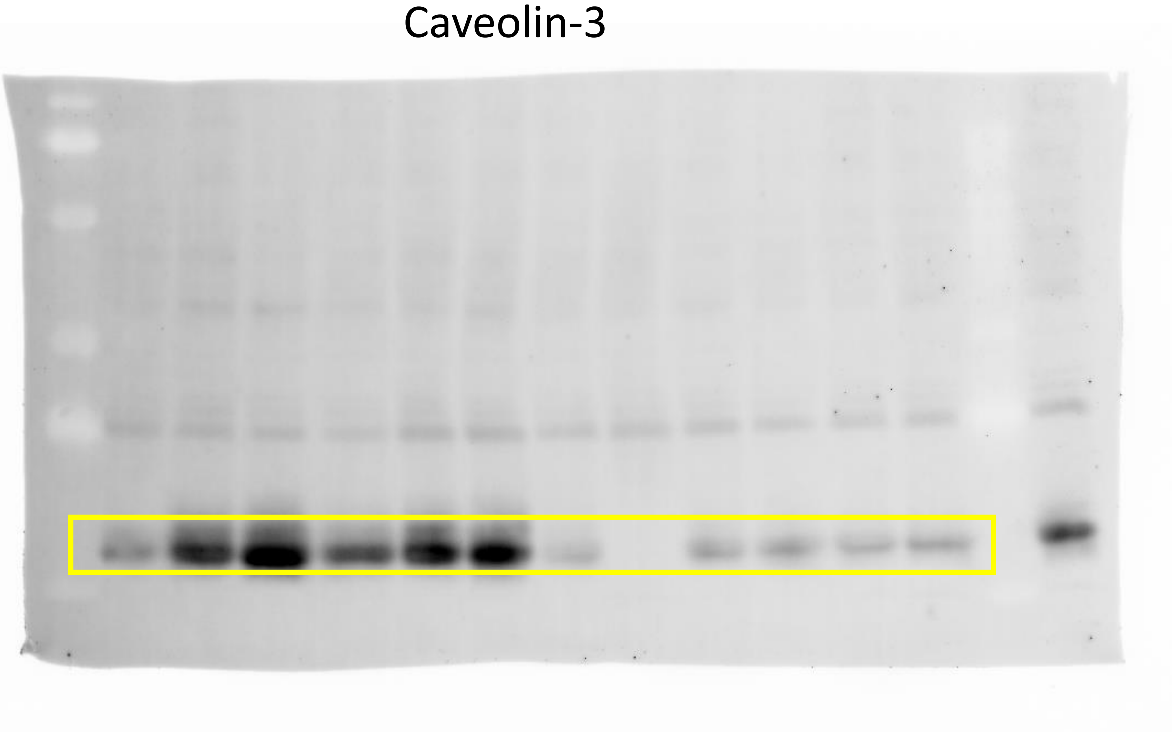

GAPDH

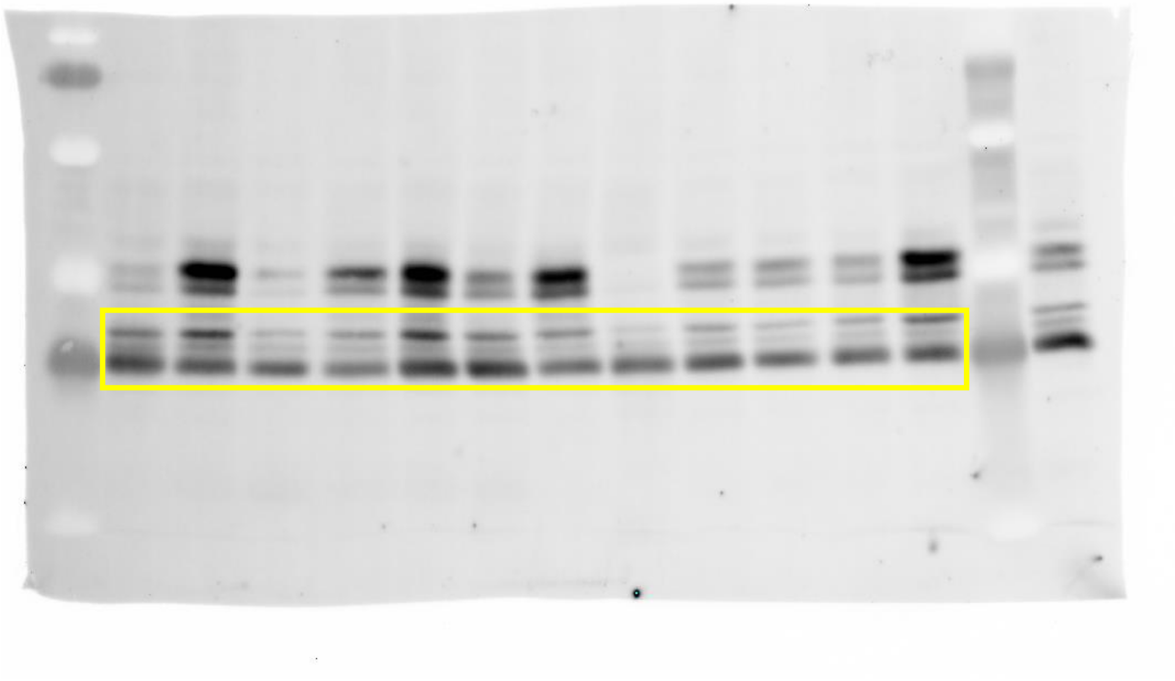

Full unedited gels for Supp. Figure 2A

pCaMKII

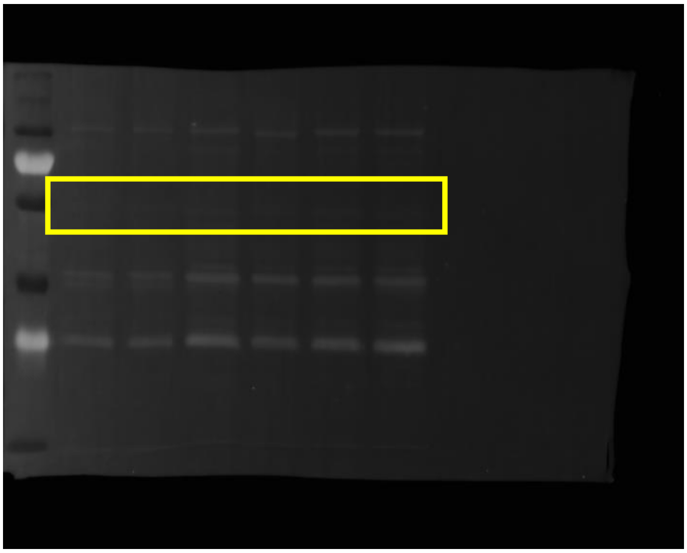

GAPDH

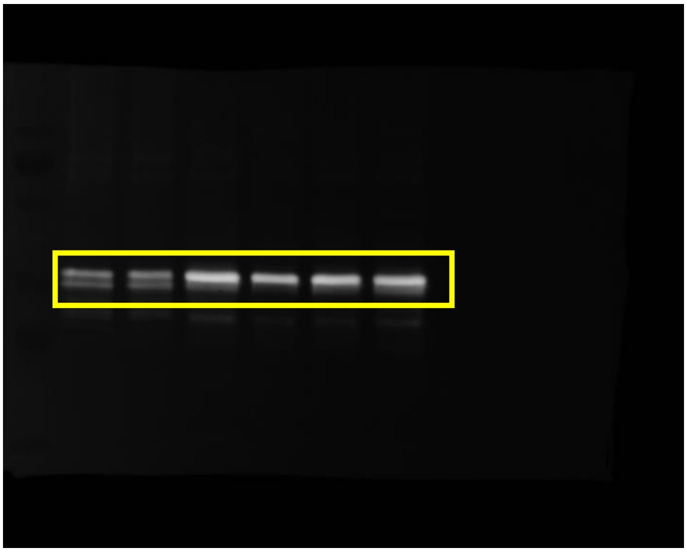

pCaMKII

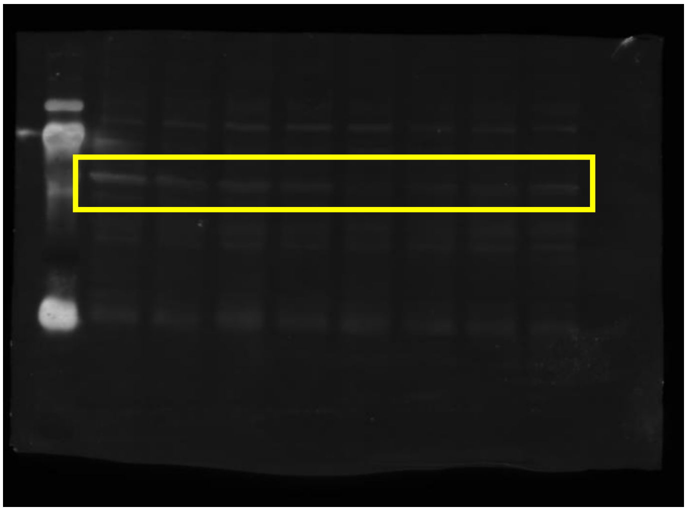

GAPDH

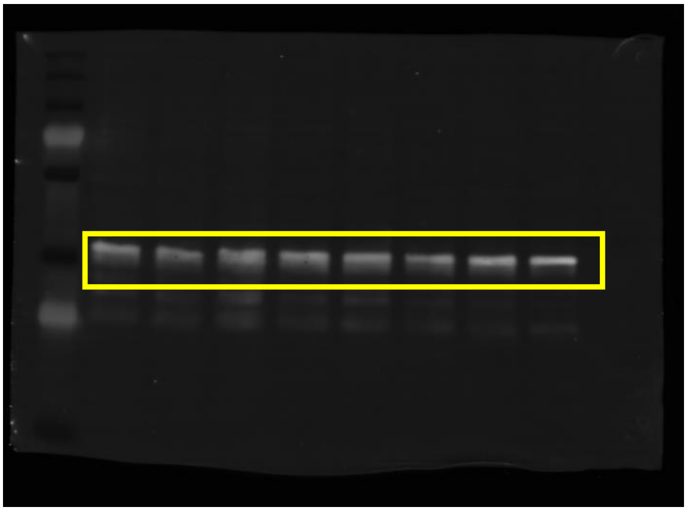

Full unedited gels for Supp. Figure 2C

pCaMKII

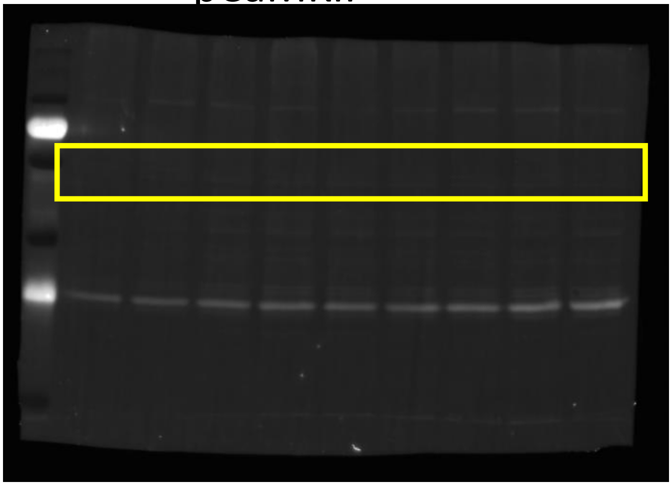

GAPDH

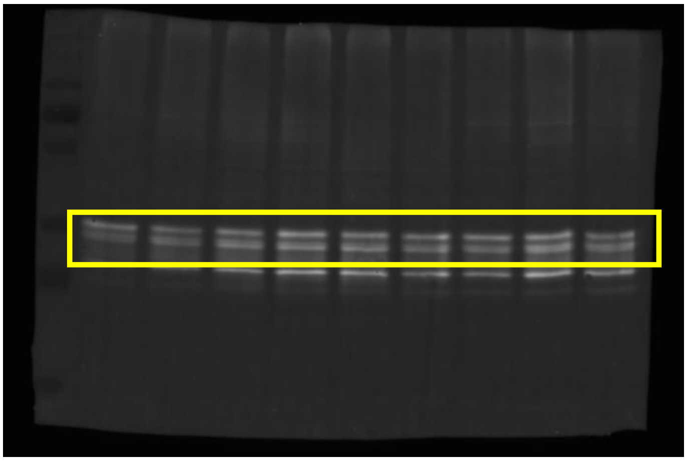

Full unedited gels for Supp. Figure 2E

pCaMKII

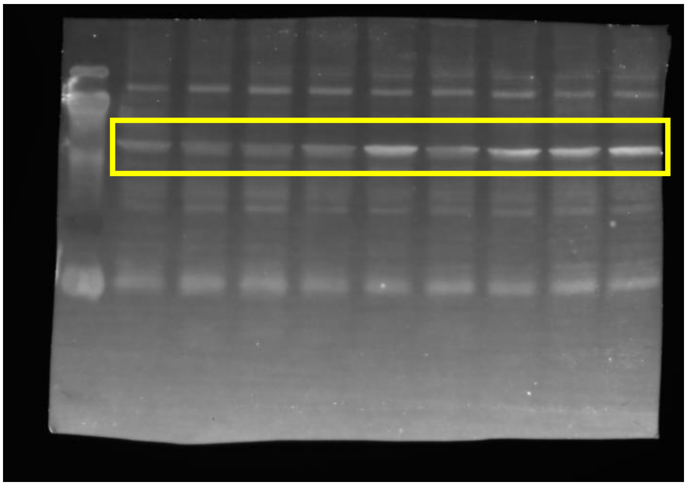

GAPDH

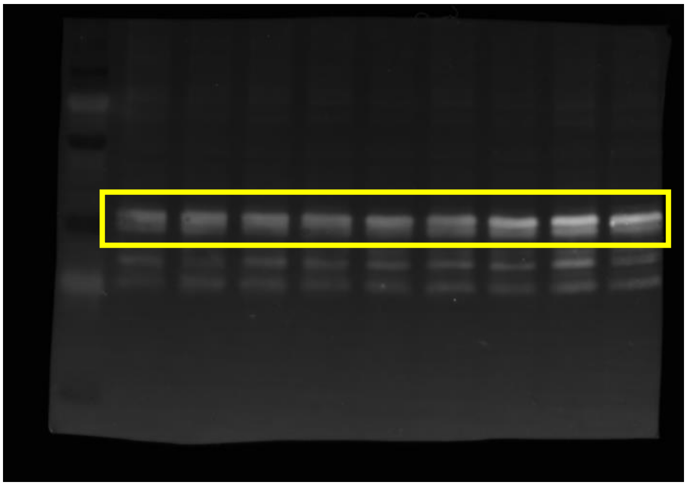

Supplement: Supplementary file 4 [file res-133-120-s004.pdf]
